# Side‐Chain Control of Topochemical Polymer Single Crystals with Tunable Elastic Modulus

**DOI:** 10.1002/anie.202213840

**Published:** 2022-10-26

**Authors:** Zitang Wei, Xiaokang Wang, Bumjoon Seo, Xuyi Luo, Qixuan Hu, Jack Jones, Matthias Zeller, Kang Wang, Brett M. Savoie, Kejie Zhao, Letian Dou

**Affiliations:** ^1^ Davidson School of Chemical Engineering Purdue University West Lafayette IN 47907 USA; ^2^ School of Mechanical Engineering Purdue University West Lafayette IN 47907 USA; ^3^ Department of Chemistry Purdue University West Lafayette IN 47907 USA; ^4^ Birck Nanotechnology Center Purdue University Purdue University West Lafayette, IN USA

**Keywords:** Elastic Modulus, Polymer Processing, Side-Chain Control, Single Crystalline Polymer, Topochemical Polymerization

## Abstract

Topochemical polymerizations hold the promise of producing high molecular weight and stereoregular single crystalline polymers by first aligning monomers before polymerization. However, monomer modifications often alter the crystal packing and result in non‐reactive polymorphs. Here, we report a systematic study on the side chain functionalization of the bis(indandione) derivative system that can be polymerized under visible light. Precisely engineered side chains help organize the monomer crystals in a one‐dimensional fashion to maintain the topochemical reactivity. By optimizing the side chain length and end group of monomers, the elastic modulus of the resulting polymer single crystals can also be greatly enhanced. Lastly, using ultrasonication, insoluble polymer single crystals can be processed into free‐standing and robust polymer thin films. This work provides new insights on the molecular design of topochemical reactions and paves the way for future applications of this fascinating family of materials.

## Graphical abstract

Topochemically reactive single crystals of bis(indandione) derivatives were carefully functionalized by controlling side chain length as well as introducing different end groups on the side chains. While maintaining topochemical reactivities, modifying side chains can significantly affect polymerization kinetics and enhance polymer elastic modulus. Furthermore, polymer single crystals could be processed into free‐standing and robust thin films.

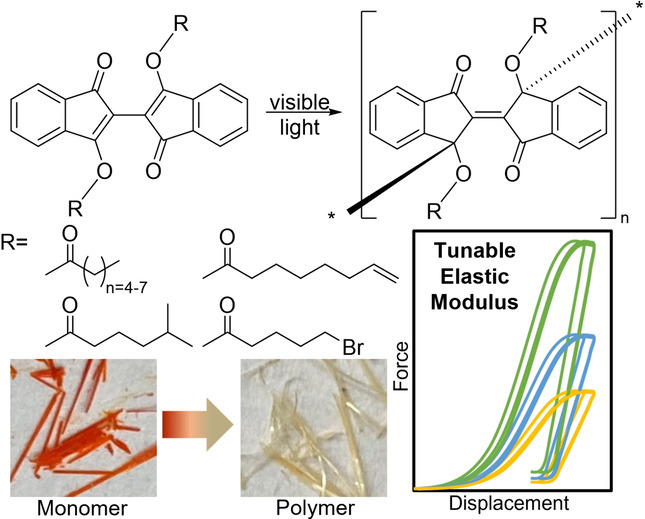

## Introduction

Topochemical polymerizations (TCPs) have attracted considerable attention over traditional solution‐phase polymerization because of the solvent‐ and catalyst‐free conditions, simultaneously offering highly stereoregular products in high yield.^[1]^ TCPs can produce high molecular weight polymers without tedious purifications. Various attempts have been made to investigate topochemical reactions, including cycloaddition reactions among polyolefins,^[2]^ oligo(aza)anthracene,^[3]^ alkynes/azide^[4]^ and additions reactions between dienes,^[5]^ diynes,^[6]^ trienes,^[7]^ triynes,^[8]^ and quinodimethanes.^[9]^ Yet, TCPs require specific packing of monomers in the crystalline state,^[10]^ where the monomers are arranged as a result of the sum of intermolecular interactions.^[11]^ From the molecular design point of view, modifying monomers in TCPs is quite challenging. As Schmit proposed, the initiation of topochemical reactions requires the reacting groups to maintain a near planar orientation within an optimum distance of 3.5–4.2 Å in the crystal lattice.^[12]^ Small modifications on molecules can alter the packing of crystal lattices, displacing the reactive sites from optimal orientation and separation.^[13]^ Competition between intermolecular interactions, including hydrogen bonding,^[14]^ π‐π interactions,^[15]^ halogen‐halogen interactions,^[16]^ and CH‐π interactions^[11]^ are recognized to strongly affect the final crystal structures.^[11]^ Therefore, the principal challenge of TCP is designing monomers that both form crystal structures conducive to polymerization,^[10a]^ and yield polymers with useful properties.

Beyond modifying crystal lattices of organic crystals, intermolecular non‐covalent interactions can also influence the mechanical properties. Inside organic crystals, intermolecular interactions can often form a closely packed network.^[16]^ It has been reported that interactions such as hydrogen bonding in organic materials can improve several mechanical properties including elastic modulus,^[17]^ hardness,^[18]^ and ductility.^[19]^ However, there exist few polymer materials under TCPs with tunable mechanical properties.[^10a^, ^17b^] Furthermore, the usage of topochemical single crystals is quite limited because there are very few processing methods to convert polymer single crystals into useful forms. Only a handful of examples use either soluble crystals to process into plastic films^[20]^ or organogels to process into fibers.^[21]^ But most topochemical single crystals are insoluble in common organic or inorganic solvents,^[10b]^ creating a major hurdle and preventing these polymer single crystals from potential usability in emerging applications.

In this study, we demonstrate the synthesis, single crystal preparation, and polymer processing of topochemical reactive bis(indendione) derivatives (BIT) by systematically varying functional groups on the side chains. We also studied the impact of side chains modifications on photochemical reactivities and polymer elastic modulus responses. We found that with the help of intermolecular interactions, all the photopolymerizable single crystals of BITs form a one‐dimensional (1D) conformational lock in the crystal lattices to maintain the reactive site distances (*d*_C−C_) in a range of 3.244–3.787 Å and backbone torsional angles (*θ*_t_) between 0° and 26.8°. Kinetic studies of photopolymerizable crystals revealed that modifying side chains also significantly affect the polymerization rate. Additionally, to understand the relationship between the side chains and mechanical properties of the resulting polymers, nanoindentation tests were performed to measure the elastic modulus of seven PBIT polymer single crystals. Introducing bromine atom on side chains in PBIT‐5‐Br leads to highest elastic modulus of 10.6 GPa. Finally, we utilized an ultrasonication treatment followed by vacuum filtration and heat pressing to process 1D polymer single crystals into 2D free‐standing polymer thin films. Among different PBIT polymer thin films, PBIT‐5‐Br thin films also exhibit the best performance with a tensile modulus of 625 MPa and a tensile strength of 6.8 MPa.

## Results and Discussion

**Figure 1:**
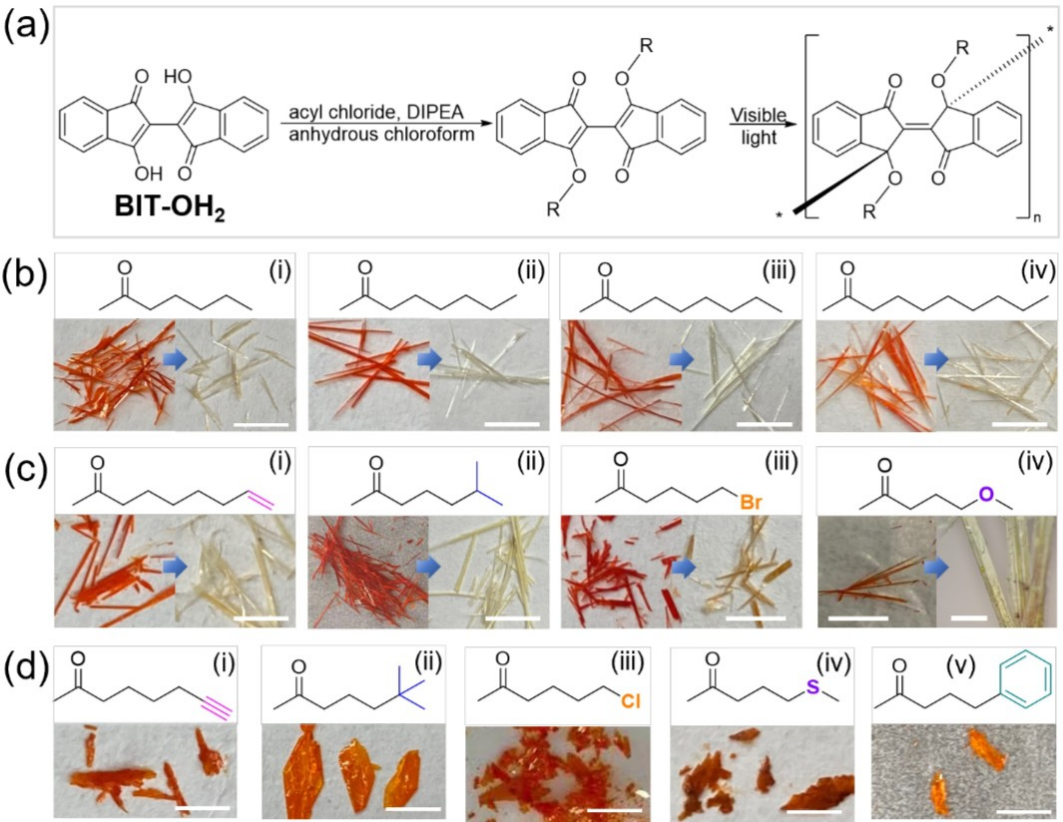
a) Synthetic pathway of BIT monomers and polymers. b) Structure of side chains (top) and images of BIT monomers (bottom left) and polymers (bottom right) with saturated linear alkylcarboxylate side chains. (i) BIT‐6 and PBIT‐6. (ii) BIT‐7 and PBIT‐7. (iii) BIT‐8 and PBIT‐8. (iv) BIT‐9 and PBIT‐9. c) Structures of side chains (top) and images of BIT monomers (bottom left) and polymers (bottom right) with modified side chains. (i) BIT‐8D and PBIT‐8D. (ii) BIT‐6‐Me and PBIT‐6‐Me. (iii) BIT‐5‐Br and PBIT‐5‐Br. (iv) BIT‐6‐O and PBIT‐6‐O. Blue circle labels unpolymerized crystals of BIT‐6‐O even under visible light irradiation over one week. d) Structures of side chains (top) and images of BIT monomers (bottom) with modified side chains that cannot be photopolymerized. (i) BIT‐7T. (ii) BIT‐6‐2Me. (iii) BIT‐5‐Cl. (iv) BIT‐6‐S. (v) BIT‐4‐Ph. All the scale bars are 5 mm, only PBIT‐6‐O image scale bar is 100 μm.

First synthesized and characterized by Gabriel and Leopold in 1898,^[22]^ the dye molecule [2,2′‐bi‐1H‐indene]‐3,3′‐dihydroxy‐1,1′‐dione (BIT‐OH_2_) has proven to be a reliable backbone to synthesize photopolymerizable single crystals. Several reports have investigated the topochemical reactivity of this dye by functionalizing BIT‐OH_2_ with saturated alkylcarboxylates on the hydroxyl groups.^[23]^ By decreasing the reaction temperature using salt ice bath and deprotonating BIT‐OH_2_ with a bulky base such as N,N‐diisopropylethylamine (DIPEA), reactions between BIT‐OH_2_ and corresponding acyl chlorides were improved with over 75 % yield (Figure 1a). Monomer single crystals were prepared by dissolving the materials into a dichloromethane‐methanol mixture followed by slow evaporation at room temperature in the dark. High quality single crystals with over 5 mm in length were obtained for all monomers (Figure 1).

Several approaches were considered for side chain design including altering the side chain length and bulkiness, as well as introducing unsaturated groups, heteroatoms, and phenyl rings.

As the side chain lengths were increased from six carbons (BIT‐6) to nine carbons (BIT‐9), all monomer crystals presented 1D crystals and were photopolymerized to corresponding poly‐bis(indandione) derivatives (PBIT) when irradiated with visible light (Figure 1b). As all other monomer crystals with different functional groups were prepared and irradiated with visible light, a very interesting correlation was observed: only needle‐like 1D crystals do undergo photopolymerization while two‐dimensional (2D) plate‐like crystals do not (Figure 1).

**Figure 2:**
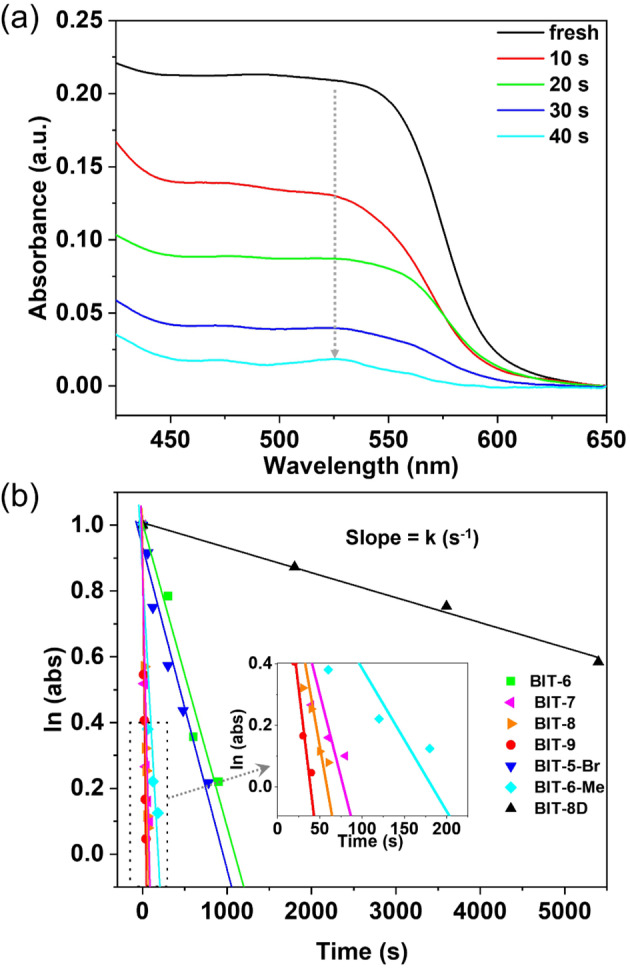
Photopolymerization kinetic analysis. a) Change in UV/Vis spectrum during the crystalline‐state polymerization of BIT‐9 under visible light irradiation at room temperature. Gray arrow represents the change of signature absorption peak around 525 nm. b) Semilogarithmic plots of monomer UV/Vis absorption versus visible light irradiation time during photopolymerization of all BIT monomer single crystals. Inset is the enlarged image of the rectangular area of the plot.

For photopolymerizable crystals, quantitative monomer‐polymer conversion was achieved with various irradiation times. To further study the polymerization kinetics, the polymerization rates were examined via tracking of the UV/Vis absorption change during polymerization. Figure 2a presents a survey of change in absorption with respect to visible light irradiation time of BIT‐9 crystals (data for other six crystals can be found in Figure S1–S6, Supporting Information). The change of the characteristic absorption peak around 525 nm (labeled in gray arrow in Figure 2a) with respect to irradiation time was used to determine the rate constant of polymerization. The polymerization reaction approximately follows first order reaction kinetics with regard to the absorbance of monomer crystals. Figure 2b shows the relationship between absorbance and the photopolymerization time of all polymerizable monomer crystals. The rate constants k determined from the slope of the lines are summarized in Table 1. It shows that the side chain length affects the rate constants: As the side chain length increases from BIT‐6 to BIT‐9, the polymerization rate constant increases from 1.78×10^−3^ s^−1^ to 7.33×10^−2^ s^−1^ (Table 1). In addition, introducing different functional groups does also significantly affect the polymerization rate: The rate constant of BIT‐8D, which has a terminal double bond on the side chain, is more than two order of magnitude smaller than that of BIT‐8.

**Table 1:** Summary of polymerization rate constants of BIT monomer crystals.

Monomer	Polymerization Rate Constant *k×*10^3^ [s^−1^]	Monomer	Polymerization Rate Constant *k×*10^3^ [s^−1^]
BIT‐9	73.3±11	BIT‐6‐Me	11.3±1.2
BIT‐8	43.4±3.6	BIT‐5‐Br	1.91±0.086
BIT‐7	28.8±1.4	BIT‐8D	0.0977±0.011
BIT‐6	1.78±0.24		

**Figure 3:**
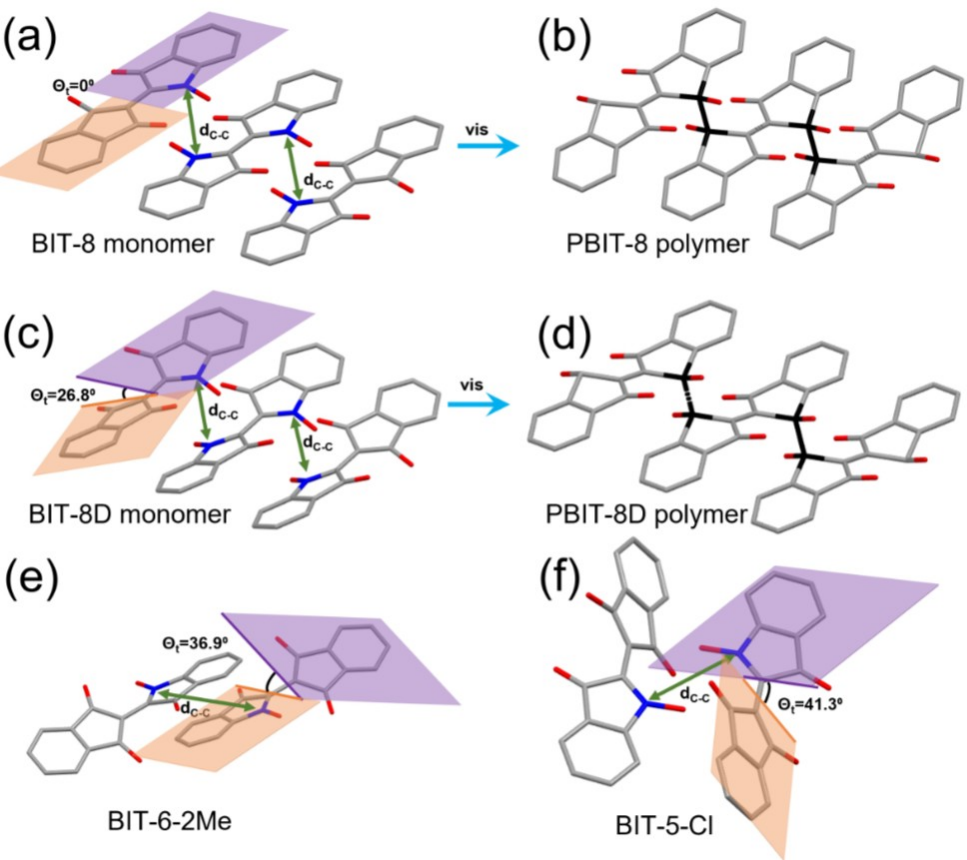
Crystal structure characterization of monomers and polymers. Reactive site carbons are labeled in blue in monomers. Newly formed carbon‐carbon single bond in polymers is labeled in black. All side chains and hydrogens are removed for clarity. a) Crystal structure of BIT‐8 monomer. b) Crystal structure of PBIT‐8 polymer. c) Crystal structure of BIT‐8D monomer. d) Crystal structure of PBIT‐8D polymer. e) Crystal structure of BIT‐6‐2Me. f) Crystal structure of BIT‐5‐Cl. C without specific label, grey; O, red.

To understand different polymerization behaviors among BIT monomer crystals, single crystal structures of all monomer and polymer crystals were resolved by single‐crystal X‐ray diffraction (XRD). The crystallographic data for the X‐ray single crystal structures and single crystal analysis are summarized in Figure S7–S27 and Table S1. It is accepted that the distance between two reactive carbons that form a new carbon‐carbon single bond after photopolymerization (*d*_C−C_) is a key parameter to determine the topochemical reactivity.[^7^, ^10b^, ^11^, ^12^] For the BIT system, we introduced another parameter, the torsional angle (*θ*_t_), to determine the reactivity. The torsional angle is defined as the angle between the two indene substituents on the backbone (Figure 3). Using *d*_C−C_ and *θ*_t_, 14 monomer crystals can be categorized into two groups: photopolymerizable monomers and photostable monomers. All photopolymerizable monomer crystals share similar *d*_C−C_ within 3.19 Å‐3.85 Å and *θ*_t_ smaller or up to 26°. Photostable monomer crystals have *d*_C−C_ larger than 3.85 Å and *θ*_t_ larger than 26° (Table 2).

**Table 2:** Selected geometry parameters and polymerization rate constants for BIT monomer crystals.

Monomer	Reactive Site Distance, *d*_C−C_ [Å]	Torsional Angle, *θ*_t_ [°]	Polymerization YES/NO
BIT‐9	3.273	0	YES
BIT‐8	3.244	0	YES
BIT‐7	3.263	0	YES
BIT‐6‐Me	3.264	2.26	YES
BIT‐5‐Br	3.189	0	YES
BIT‐6	3.245	0	YES
BIT‐8D	3.787	26.79	YES (slow)
BIT‐6‐O	3.854	25.06	YES (slow)
BIT‐4‐Ph	3.897	24.47	NO
BIT‐5 (one polymorph)	3.925	25	NO
BIT‐7T	5.441	32.1	NO
BIT‐6‐S	5.692	35.8	NO
BIT‐6‐2Me	5.767	36.9	NO
BIT‐5‐Cl	5.173	41.3	NO

First side chain design strategy was to tune the side chain length. For saturated linear alkylcarboxylate side chains (BIT‐6,7,8,9), all the *d*_C−C_ lengths are within a range of 3.244–3.273 Å, and all angles *θ*_t_ are 0°. However, when the side chain length was reduced to five carbon atoms (BIT‐5), two different polymorphs were obtained that became difficult to separate from each other. After shining visible light on the crystals, one polymorph is photopolymerizable and the other is photostable (Figure S28, Supporting Information). Only for the photostable polymorph were we able to determine the single crystal structure, which gave *d*_C−C_ of 3.925 Å and *θ*_t_ of 26° (Table 2), which correspond to the photostable monomer category. Without structural data for the polymerizable polymorph and to avoid the difficulties of separating multiple polymorphic structures, the side chain lengths were controlled to be 6–9 atoms for all comparative studies.

When unsaturated groups or bulky substituents were introduced to the side chains, substantial changes occurred in crystal packings. After incorporating a rigid double bond at the end of the eight‐carbon side chain (BIT‐8D), a 1D crystal form that can be photopolymerized was obtained. However, *d*_C−C_ (3.787 Å) and *θ*_t_ (26.79°) for BIT‐8D are significantly larger than those of BIT‐8 (3.244 Å, and 0°) (Figure 3a–d). The large reactive site distance and torsional angle make photopolymerization harder, resulting in a more than two order of magnitude slower polymerization rate constant for BIT‐8D than that of BIT‐8 with a saturated eight‐carbon side chain (Table 1). When a triple bond was added to the end of the seven‐carbon side chain (BIT‐7T), the crystal habit completely changed from 1D needle‐like crystals to 2D plate‐like and photostable crystals. The linear and rigid triple bond on the side chain takes up more space than BIT‐7, pushing the backbones away from each other with *d*_C−C_ of 5.441 Å and twisting the two indene derivatives with *θ*_t_ of 32.1° (Figure S29a, Supporting Information). Similarly, when an iso‐butyl group was introduced to the side chain (BIT‐6‐Me), the packing was disturbed by the two free‐rotating methyl groups with a slightly twisted torsional angle (*θ*_t_=2.26°) (Figure S29b, Supporting Information), but the 1D crystal form was maintained as well as photopolymerizability. In contrast, introducing tert‐butyl group massively changed the crystal packing and a plate‐like crystal with *d*_C−C_ of 5.767 Å and *θ*_t_ of 36.9° was obtained (Figure 3e).

Another approach of modifying the side chains is to substitute carbons with heteroatoms. Reports about other topochemical reaction systems have shown that including heteroatoms can influence the crystal packings by introducing different non‐covalent interactions such as hydrogen bonding and halogen bonding.[^11^, ^16^] Here we introduced chalcogens (oxygen for BIT‐6‐O and sulfur for BIT‐6‐S) and halogens (chlorine for BIT‐5‐Cl and bromine for BIT‐5‐Br) to the side chains. Among these monomer candidates, only BIT‐6‐O and BIT‐5‐Br adopted crystal packings that permitted photopolymerization. The *d*_C−C_ (3.854 Å) and *θ*_t_ (25.1°) for BIT‐6‐O places it at the limits of the photopolymerizable criteria and BIT‐6‐O monomers crystals could not be fully polymerized. An optical image of a partially polymerized BIT‐6‐O crystal shows some monomer left even after shining visible light for over one week (unpolymerized crystals are labeled by a blue circle in Figure 1c(iv)). Among the monomers with halogens, BIT‐5‐Br has the shortest reactive site distance (3.189 Å) and no twist (*θ*_t_=0°) (Table 2), while BIT‐5‐Cl has a large reactive site distance (5.173 Å) and torsional angle (41.3°) (Figure 3f).

Our final attempt of side chain modification was to introduce phenyl rings on side chains. The side chain with a phenyl ring and a four‐carbon linker (BIT‐4‐Ph) was used to match the length of six‐carbon side chain. BIT‐4‐Ph exhibits a 2D plate‐like crystal that is photostable (Figure 1d (v)). The single crystal structure of BIT‐4‐Ph shows a *d*_C−C_ of 3.897 Å and *θ*_t_ of 24.47° (Table 2), which are at the limit of the photopolymerizable criteria.

Different from many topochemical polymerization systems that only use reactive site distance as the parameter, using torsional angle as another parameter can efficiently distinguish topochemical reactivities between BIT monomer crystals. For example, the reactive site distances of BIT‐5 (3.897 Å) and BIT‐4‐Ph (3.925 Å) lie in the empirical range of topochemical polymerizations (3.5–4.2 Å),[^10b^, ^12^] but the relatively large torsional angles (≈25°, Table 2) keep the monomer crystals from being photopolymerized.

**Figure 4:**
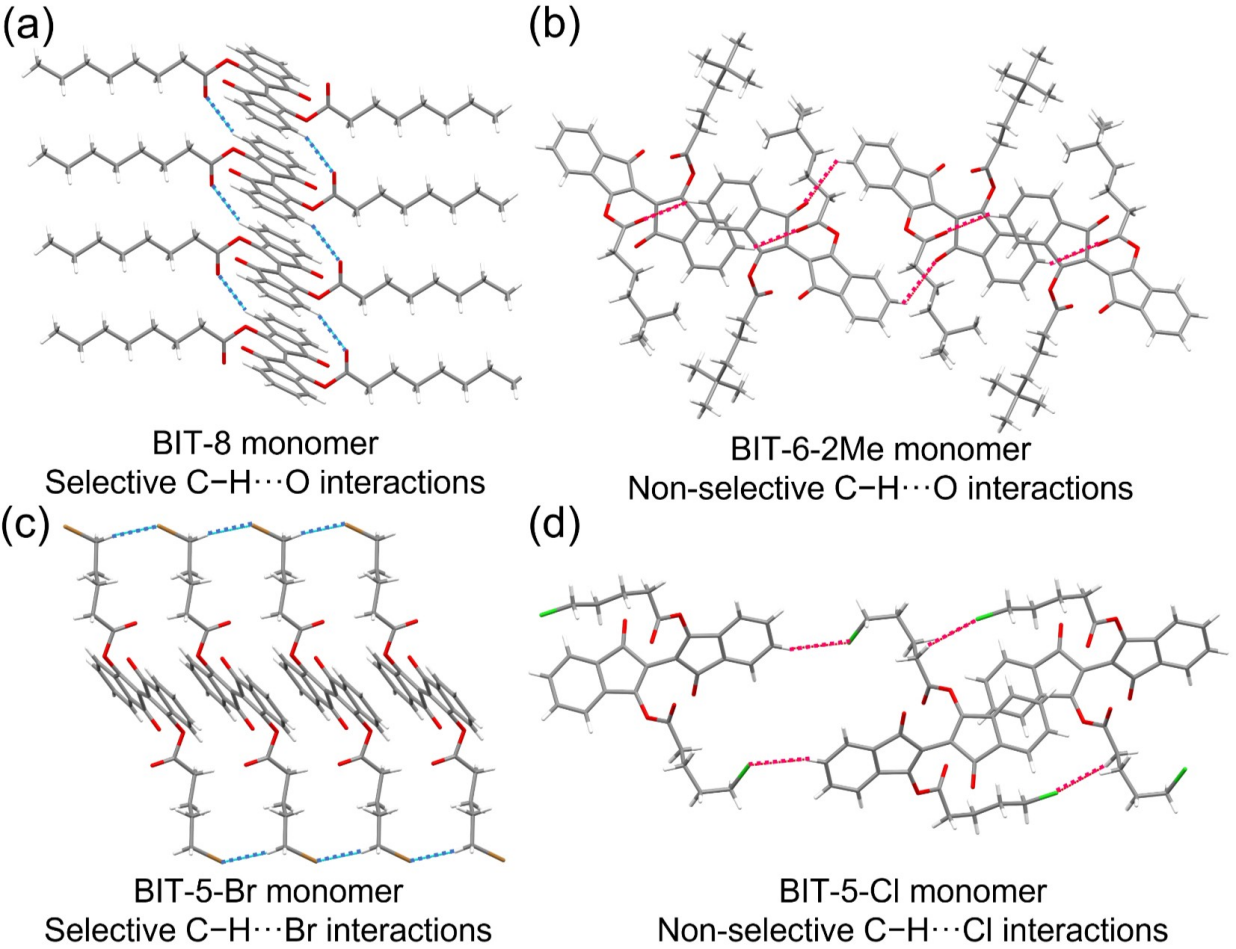
Analysis of intermolecular non‐bonding interactions. a) Single crystal structure of BIT‐8 with side chains. Selective C−H⋅⋅⋅O hydrogen bonding is labeled in blue dotted line. b) Single crystal structure of BIT‐6‐2Me with side chains. Non‐selective C−H⋅⋅⋅O hydrogen bonding is labeled in red dotted line. c) Single crystal structure of BIT‐5‐Br. Selective C−H⋅⋅⋅Br interactions are labeled in blue dotted line and recognized as tail‐to‐tail conformation to help maintain linear monomer chain. d) Crystal structure of BIT‐5‐Cl. Non‐selective C−H⋅⋅⋅Cl interactions is labeled in red dotted line and can be found between Cl and different hydrogens.

We next investigate the molecular origins determinants of the observed crystal packings, topochemical reactivities, and kinetics. In BIT crystals, π‐π interactions provide strong driving forces for molecular packing. With side chain modifications, π‐π stackings in some of the monomers are disturbed and *d*_C−C_ and *θ*_t_ are greatly influenced. In addition, hydrogen bonds and other non‐covalent interactions play important roles. For all 1D photopolymerizable crystals, they share a weak but selective C−H⋅⋅⋅O hydrogen bonding between the hydrogen atom on the backbone and the oxygen atom on the side chain (this C−H⋅⋅⋅O hydrogen bonding interaction is labeled by a blue dotted line in Figure 4a for BIT‐8). This type of hydrogen bonding provides a conformational lock to promote crystal packing into a 1D fashion.

These hydrogen bonds align nicely with the backbone stacking direction to maintain the backbones close enough for topochemical reactions to occur. In comparison, all 2D non‐polymerizable monomer crystals have non‐selective C−H⋅⋅⋅O hydrogen bonding that present themselves randomly in the monomer crystals (non‐selective C−H⋅⋅⋅O hydrogen bonding is labeled as a red dotted line in Figure 4b for BIT‐6‐2Me). After introducing extremely bulky groups such as tert‐butyl group (BIT‐6‐2Me, Figure 4b) or a very rigid unsaturated group such as triple bond (BIT‐7T, Figure S30, Supporting Information), free rotations of those terminal groups expanded the crystal lattices so that the roles of π‐π stacking and conformational lock were diminished. For some specific monomer crystals, C−H⋅⋅⋅O hydrogen bonding also presents itself among side chains, which can also influence polymerizability. BIT‐6 monomer crystals have C−H⋅⋅⋅O hydrogen bonds between hydrogen on the end of the side chain and carbonyl oxygen on the nearby side chain with a distance of 2.577 Å (Figure S31a, Supporting Information). After polymerization, the distance between the same two atoms becomes 2.855 Å, which is larger than the sum of their van der Waals radii (Figure S31b, Supporting Information). In other words, during the topochemical reaction of BIT‐6 crystals, this specific hydrogen bond becomes a barrier to overcome, leading to a much smaller polymerization rate constant of BIT‐6 (1.78×10^−3^ s^−1^) than that of other BIT monomers without this type of interaction (such as BIT‐8 with a polymerization rate constant of 43.3×10^−3^ s^−1^, Table 1, Figure S32).

Regarding other non‐covalent interactions, we introduced C−H⋅⋅⋅halide interactions by swapping the terminal methyl group on the side chain of BIT‐6 for chlorine or bromine atoms. Surprisingly and unexpectedly, the two monomer crystals with halides gave completely different and distinct crystal habits and topochemical reactivities. In the crystal structures, BIT‐5‐Br shows selective C−H⋅⋅⋅Br interactions in a tail‐to‐tail fashion between terminal bromine and hydrogen on the end of neighboring side chains (Figure 4c). This C−H⋅⋅⋅Br interaction propagates along the monomer backbone packing directions, helping the backbones to maintain their 1D stacking. In contrast, chlorine atoms in BIT‐5‐Cl formed non‐selective C−H⋅⋅⋅Cl interactions with hydrogens on the middle of the side chains as well as hydrogens on the backbones (Figure 4d).

**Figure 5:**
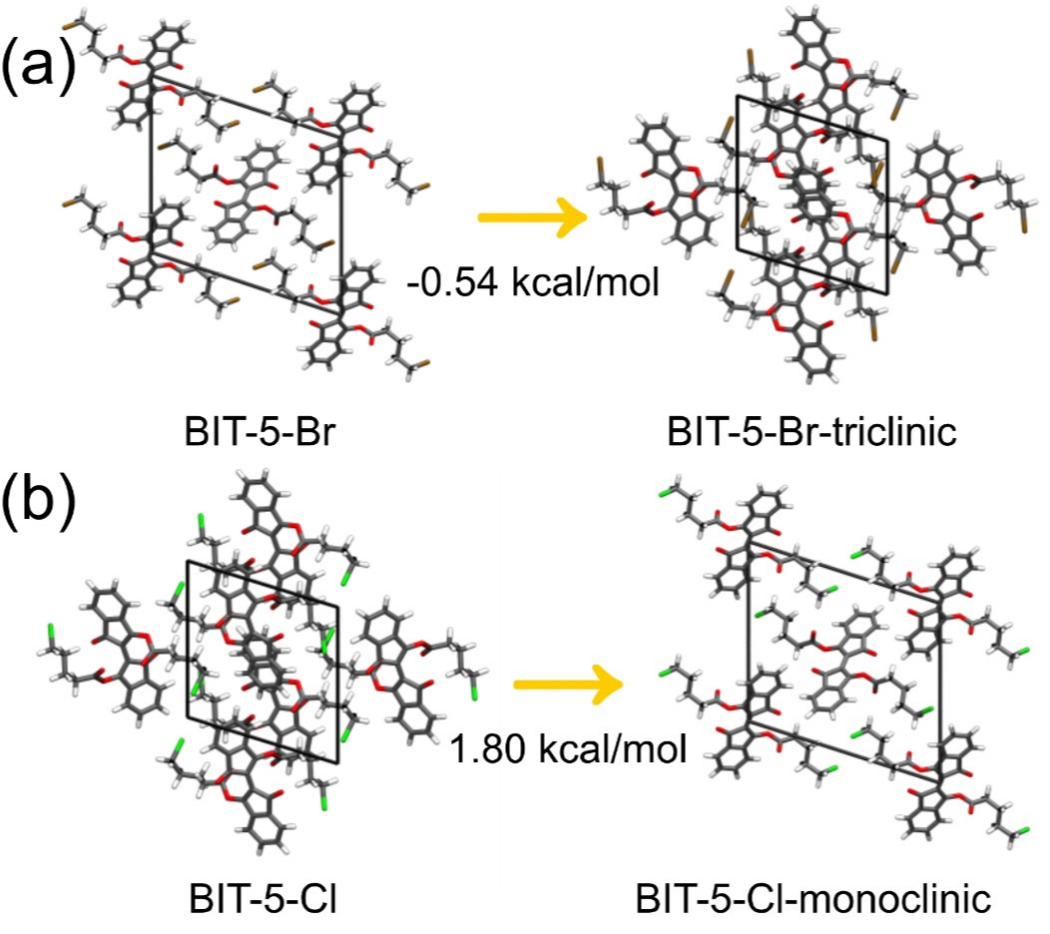
a) Differences in the lattice energy for the monoclinic BIT‐5‐Br crystal (left) to triclinic BIT‐5‐Br crystal (right). b) Differences in the lattice energy for the triclinic BIT‐5‐Cl crystal (left) to monoclinic BIT‐5‐Cl crystal (right). Br, brown; Cl, green; C, grey; O, red; H, white.

To better explain the role of the halide end groups on the crystal structures of BIT‐5‐Br and BIT‐5‐Cl, density functional theory (DFT) calculations were performed to calculate the difference in the lattice energies of the crystal structures exhibited by the different end groups. In addition to the two experimental crystal structures of the BIT‐5‐Br and BIT‐5‐Cl monomers, two crystal structures with exchanged halide groups were modeled as shown in Figure 5. Namely, the bromine atoms in the monoclinic crystal of BIT‐5‐Br were exchanged with chlorine (BIT‐5‐Cl‐monoclinic) and the chlorine atoms in the triclinic crystal of BIT‐5‐Cl were exchanged for bromine (BIT‐5‐Br‐triclinic) to establish the relative energetics and preference of the halide end groups for their respective phases.

The calculated change in the lattice energy from BIT‐5‐Br to BIT‐5‐Br‐triclinic is −0.537 kcal mol^−1^, whereas BIT‐5‐Cl to BIT‐5‐Cl‐monoclinic is 1.796 kcal mol^−1^. This reveals that the thermodynamically preferred crystal structure for the BIT monomer with both halide end groups is the triclinic structure. However, the difference in the lattice energy for BIT‐5‐Br is close to the thermal energy, as opposed to BIT‐5‐Cl where the value is more than three times larger. The smaller and more electronegative chlorine atoms on BIT‐5‐Cl can more easily fill the voids in crystals to interact with different hydrogens, thus twisting the 1D crystals into 2D. These non‐selective C−H⋅⋅⋅Cl interactions prohibit the BIT monomer with the chlorine end group from forming the molecular requirements for photopolymerization. These observations from the lattice energy differences and the hydrogen bonding networks help rationalize the distinct behaviors observed for the two halogen end groups.

**Figure 6:**
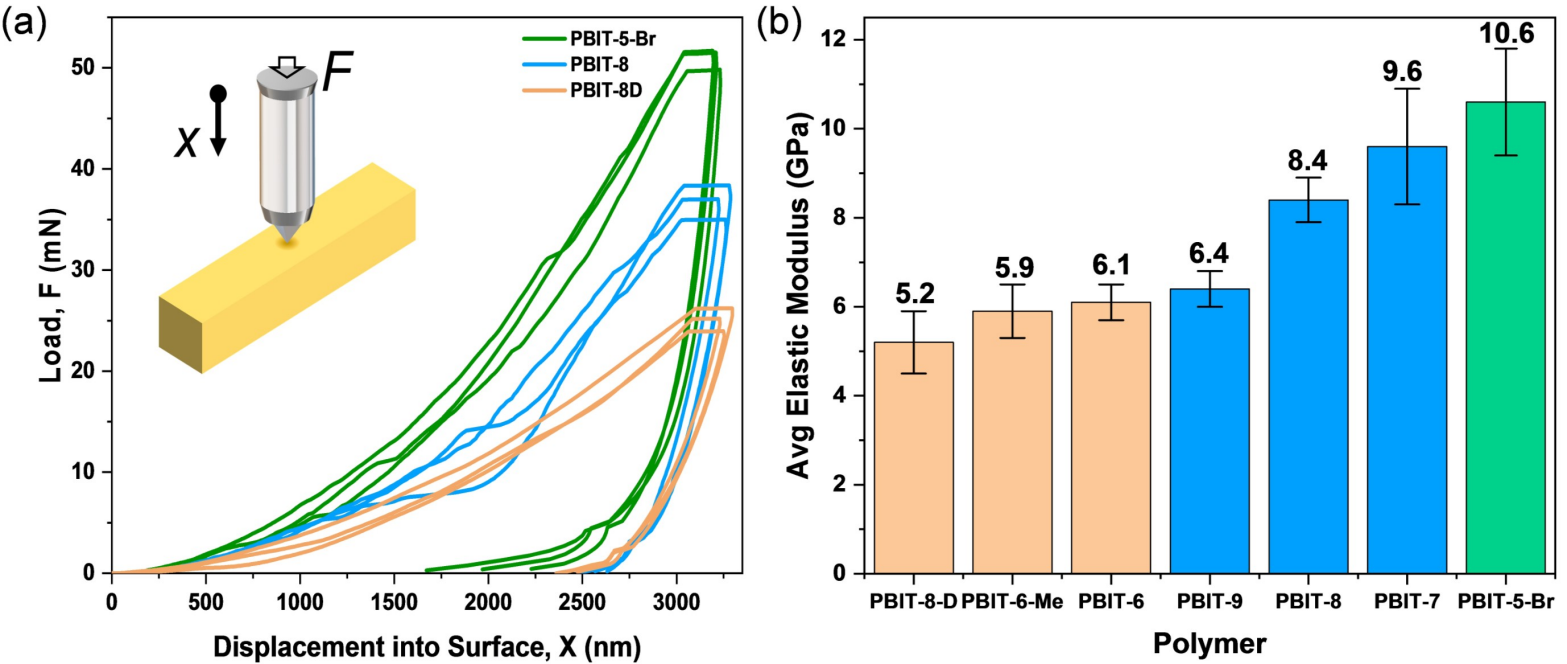
Mechanical characterization of polymer single crystals via nanoindentation. a) Load‐displacement curves of PBIT‐5‐Br, PBIT‐8 and PBIT‐8D. Inset is the schematic of the nanoindentation experiments. b) Summary of average elastic moduli for all polymer single crystals that are categorized as weak (labeled in beige), moderate (labeled in blue), and high (labeled in green). Three different crystals were measured for each polymer with ten different locations measured on each crystal.

By modifying the BIT monomers with different side chains, the topochemical reactivity and kinetics can be altered in a controlled manner. Furthermore, we postulated that incorporating functional side chains into PBIT polymers can also affect their mechanical properties. To compare the mechanical responses, nanoindentation tests were performed. We selected seven types of PBIT polymer crystals with approximately the same thickness and length. Before the tests, all the polymer samples were washed with dichloromethane to avoid monomer residues inside the polymer crystals. PBIT crystals were mounted on a glass slide with epoxy to prevent crystals from sliding. The probe penetrated the crystals in the direction orthogonal to the crystal elongation direction (inset of Figure 6a). Then the load‐displacement curves were recorded (Figure 6a with curves of PBIT‐8D, PBIT‐8 and PBIT‐5‐Br, load‐displacement curves for the other four polymer single crystals can be found in Figure S33–S36, Supporting Information). The elastic modulus was calculated as a function of the indentation depth and the average values between 2000 nm and 3000 nm are reported. Three different crystals were measured for each polymer to obtain tens of data points (snapshots of all polymers single crystals after nanoindentations can be found in Figure S37, Supporting Information). Among all the polymers, PBIT‐5‐Br has the highest elastic modulus of 10.6 GPa. PBIT‐7, PBIT‐8, and PBIT‐9 have elastic moduli between 6 and 10 GPa, which can be considered as moderate‐modulus crystals (Figure 6b). It is worth mentioning that as the side chain lengths increase, the corresponding moduli drop among the moderate‐modulus single crystals. The remaining three polymer single crystals with an average elastic modulus around or below 6 GPa are best categorized as low‐modulus crystals (Figure 6b). Young's moduli for all PBIT polymer crystals are comparable with typical organic crystals. (Organic crystals typically have Young's moduli in the range 1–25 GPa, with approximately 8 % having Young's moduli less than 1 GPa and approximately 8 % having Young's moduli larger than 25 GPa).^[24]^

To connect the observed mechanical properties with specific structural features and to build a mechanistic understanding of the behavior of these polymer single crystals, we carried out a detailed examination and evaluation of slight differences in crystal packing of all seven polymers. Regardless of the extent of elastic moduli (high, moderate, or low), for all materials no significant differences in crystal habits (all 1D needle‐ like) were found (Figure 1, Table 1). It was claimed that the elastic modulus depends on the crystal density as well as the number and strength of intermolecular interactions occurring in organic crystals.[^24^, ^25^] Among all the polymer crystals, PBIT‐5‐Br shows the highest crystal density of 1.702 g cm^−3^, while all other crystals have similar densities around 1.3 g cm^−3^(Table S2).

**Figure 7:**
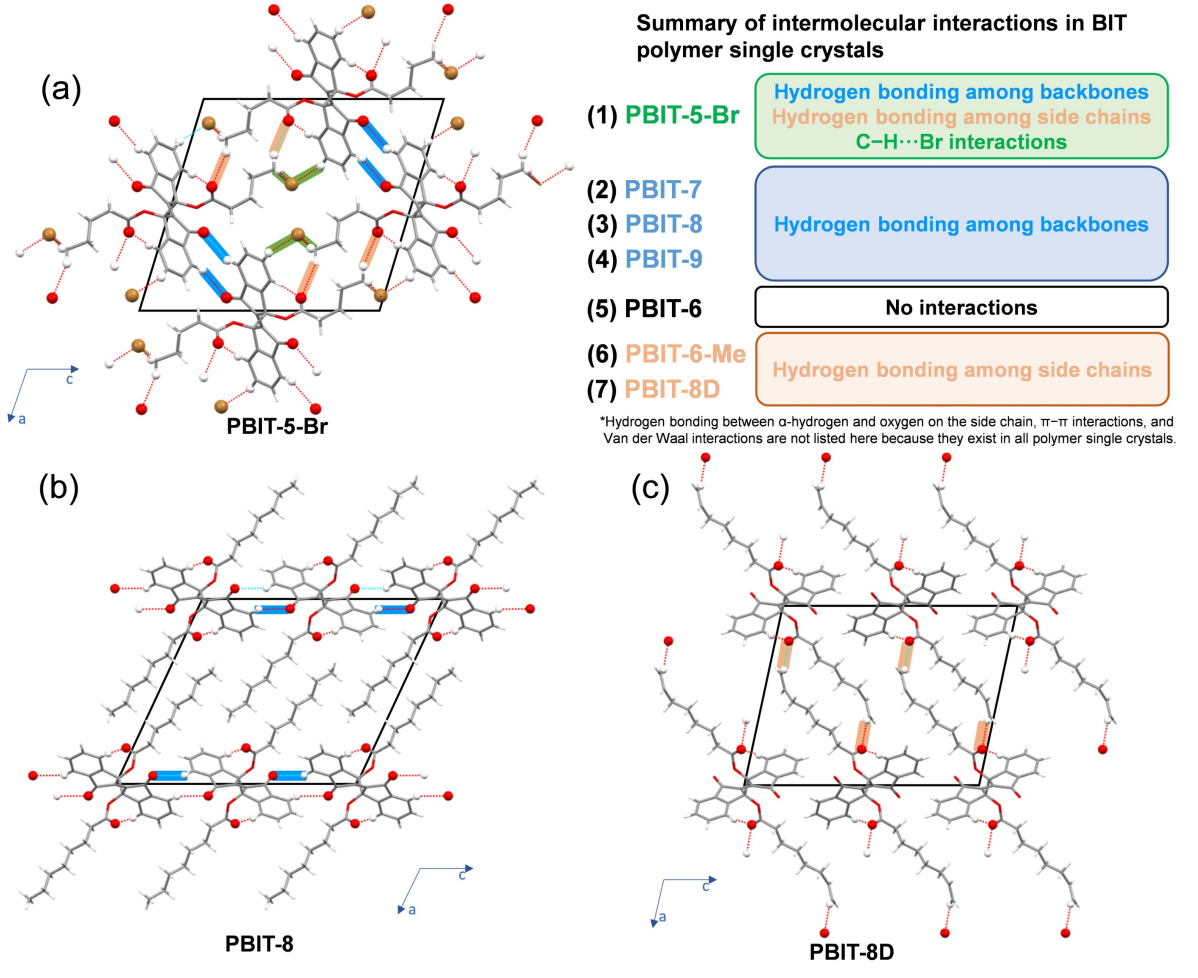
Crystallography analysis of PBIT single crystals. Hydrogen bonding among backbones is labeled in blue; hydrogen bonding among side chains is labeled in beige; C−H⋅⋅⋅Br interactions are labeled in green. a) PBIT‐5‐Br single crystal structure with side chains. b) PBIT‐8 single crystal structure with side chains. c)PBIT‐8D single crystal structure with side chains. Br, brown; C, grey; O, red; H, white. Bromine atoms, oxygen atoms, and hydrogen atoms that form hydrogen bonds are labeled in ball and stick style.

The systematic structural data in hand for these polymers also suggest that distinct differences in elastic moduli may be traced to the relative importance of specific structure‐directing intermolecular interactions. Although the premise may seem simplistic, it turns out that such an explanation provides a remarkably good fit with all the experimental data. All the polymer single crystals inherit the selective C−H⋅⋅⋅O hydrogen bonding between hydrogen on the backbone and carbonyl on the neighboring side chain like the monomer single crystals. But with different side chains, different types, and numbers of interchain interactions could be tracked that significantly impact the elastic modulus. No other interactions are observed in PBIT‐6 polymer crystal, and the corresponding average elastic modulus, which was measured to be 6.1 GPa, was thus treated as a standard for all polymers (Figure S38, Supporting Information). Within a unit cell of the PBIT‐5‐Br crystal, there are C−H⋅⋅⋅O interchain interactions within the backbones as well as between side chains (Figure 7a). The C−H⋅⋅⋅Br interactions also persist in the PBIT‐5‐Br crystals. Every single polymer chain is linked together by intermolecular and interchain interactions forming a network so that slipping is minimized between polymer chains, leading to the observed highest elastic modulus of 10.6 GPa, a 74 % improvement compared to the “standard” PBIT‐6 polymer. For polymers with moderate elastic moduli (PBIT‐7, PBIT‐8, and PBIT‐9), only C−H⋅⋅⋅O interchain hydrogen bonds within the backbones are observed (Figure 7b for PBIT‐8, Figure S39 for PBIT‐7 and PBIT‐9, Supporting Information). There are no significant interactions within the side chains regions, and the pure alkyl chain side chains isolate each layer of polymer chains from each other. As the side chain length increases from seven‐carbon to nine‐carbon, the “isolation region” (Figure S40, Supporting Information) also expands, causing a drop in elastic modulus from PBIT‐7 to PBIT‐9. For polymers with low elastic moduli (average modulus below 6 GPa), only C−H⋅⋅⋅O interchain hydrogen bonds within the side chains are realized (Figure 7c for PBIT‐8D, Figure S41 for PBIT‐6‐Me, Supporting Information). Introducing an unsaturated group (double bond in PBIT‐8D) or a bulky group (isobutyl group in PBIT‐6‐Me) interrupts the polymer crystal packing so that the backbones become isolated. The hydrogen bonds between side chains can not compensate for the structural disturbance, leading to elastic moduli for those two polymers to be lower than that of the standard PBIT‐6.

**Figure 8:**
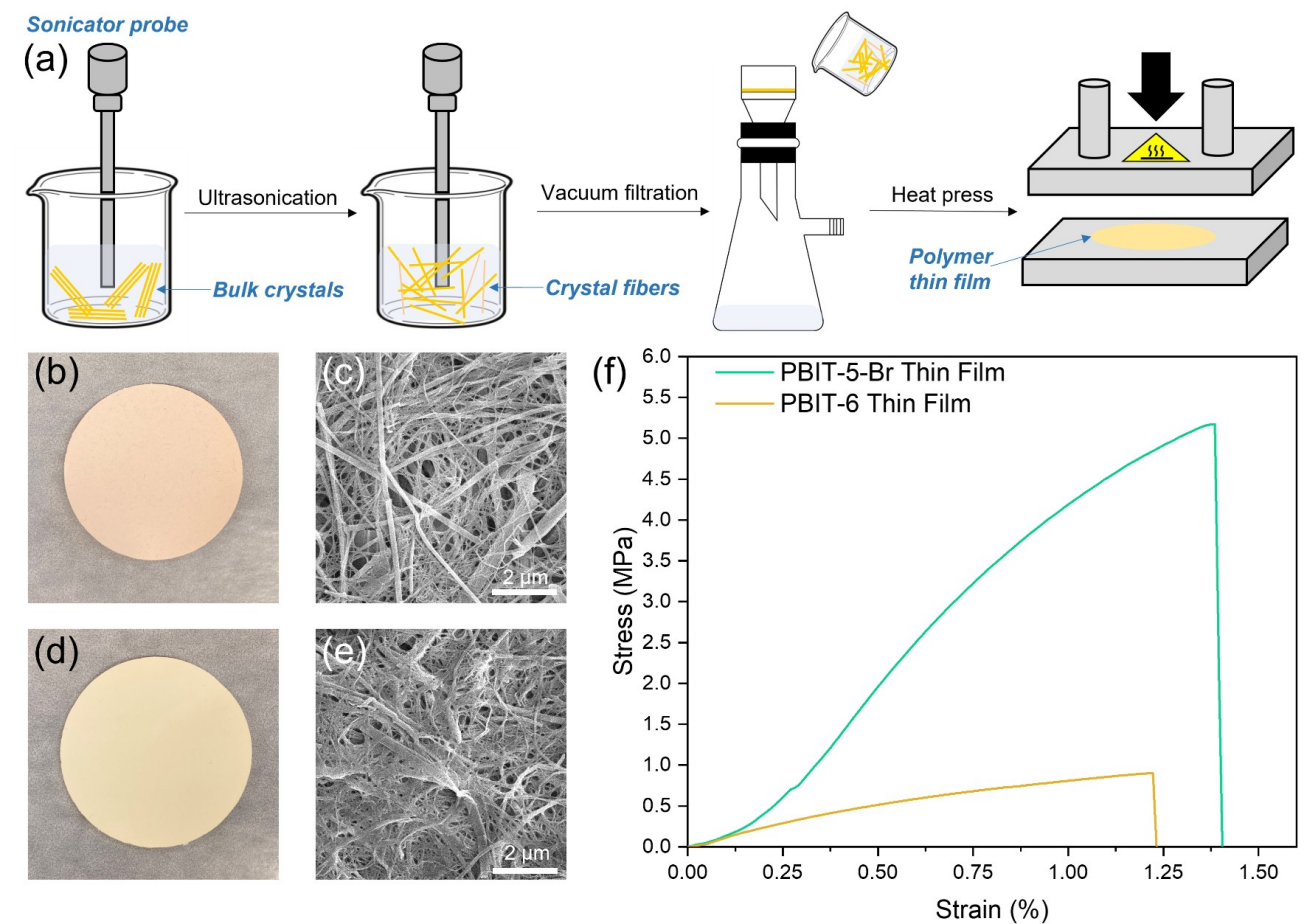
Demonstration of PBIT polymer processing. a) Scheme of processing PBIT polymer single crystals into polymer thin films. b) Image of sonication processed PBIT‐5‐Br thin film. c) SEM images of PBIT‐5‐Br thin film. d) Images of sonication processed PBIT‐6 thin film. e) SEM images of PBIT‐6 thin film. f) Representative tensile stress‐strain curves of PBIT‐5‐Br and PBIT‐6 thin films.

Topochemical polymerizations have been studied for years but using corresponding polymer single crystals in real life is quite challenging because most polymer single crystals are not soluble in any solvents. Insoluble and highly crystalline polymers are hard to process with existing methods. To overcome this problem, we developed an ultrasonication process to convert 1D crystalline PBIT polymers into free‐standing and robust thin films. As the scheme in Figure 8a demonstrates, an ultrasonic processor was used to break bulk polymer single crystals into crystal fibers. After ultrasonication of polymer crystals for 30–60 minutes in organic solvents, the crystal fibers are uniformly suspended (Figure S42, Supporting Information). Then the suspension was filtered under vacuum and heat pressed at ≈50 °C to generate a smooth thin film. Both PBIT‐5‐Br with highest elastic modulus in crystal form and standard PBIT‐6 crystals were transformed into thin films (Figure 8b, d). By controlling the ultrasonication time, interchain interactions were disturbed while the polymer chains remain intact. High‐resolution SEM images of a PBIT‐5‐Br thin film (Figure 8c) shows that after sonication, the polymer chains are straight and entangled; however, for PBIT‐6 thin film, the polymer chains are curved and much thinner (Figure 8e). Both thin films were characterized using standard tensile tests (Figure S43–S44, Supporting Information). The PBIT‐5‐Br thin film exhibits an outstanding tensile modulus of 625 MPa (avg. 552 MPa) and a tensile strength of 6.8 MPa (avg. 5.4 MPa). In comparison, PBIT‐6 thin films show a tensile modulus of only 127 MPa (avg. 108 MPa) and a tensile strength of just 2.4 MPa (avg. 2.1 MPa) (Figure 8f). Thanks to the multiple intermolecular and interchain interactions within the PBIT‐5‐Br polymers, the corresponding polymer thin films also present superior mechanical properties when compared to standard PBIT‐6 polymer thin films. This processing method could be generally utilized for other insoluble topochemical polymers and open up possibilities for using topochemical polymer single crystals in different applications such as separation membranes, filtration, and as functional device substrates.

## Conclusion

We conducted a systematic study of bis(indendione) derivatives by variation of the side chain chemistries. We found that the side chains govern the monomer molecular packing via a number of non‐covalent interactions. Although subtle, the differences in intermolecular interactions, combined with substantial structural interlocking, are critical for inducing and controlling the topochemical reactivities and elastic responses. It should be noted that designing the side chains of the BIT system provides a valuable structural handle to determine the importance of the supramolecular interactions by 1) introducing different intermolecular and interchain interactions by swapping carbons with heteroatoms, and 2) optimizing interactions by changing the side chain length. The BIT backbone has shown great tolerance towards side chains with different functional groups,^[26]^ indicating that more and other side chains could be incorporated. We have also demonstrated a general and effective way to process insoluble polymer crystals into robust thin films by ultrasonication, vacuum filtration, and heat pressing. This straightforward method offers a different pathway for topochemical polymer processing and could be used for handling other crystalline polymers. Research into other interesting topics such as how defects influence polymerizations and mechanical properties, optoelectronic properties of these polymers, and depolymerization of polymers is ongoing. Our results and analysis provide a method to rationally design molecular structures and engineer intermolecular interactions to facilitate more topochemical reactions as well as modulate their mechanical behaviors.

## Conflict of interest

The authors declare no conflict of interest.

1

## Supporting information

As a service to our authors and readers, this journal provides supporting information supplied by the authors. Such materials are peer reviewed and may be re‐organized for online delivery, but are not copy‐edited or typeset. Technical support issues arising from supporting information (other than missing files) should be addressed to the authors.

Supporting Information

## Data Availability

The data that support the findings of this study are available in the supplementary material of this article.
