# Supplementary material for: Side‐Chain Control of Topochemical Polymer Single Crystals with Tunable Elastic Modulus
Source: Angew Chem Int Ed Engl. 2022 Oct 26;61(49):e202213840. doi: 10.1002/anie.202213840 (PMC10092176; doi:10.1002/anie.202213840)
Supplement: Supplementary file 1 — Supporting Information [file ANIE-61-0-s001.pdf]

Angewandte Chemie
Eine Zeitschrift der Gesellschaft Deutscher Chemiker

Supporting Information

Side-Chain Control of Topochemical Polymer Single Crystals with Tunable Elastic Modulus

*Z. Wei, X. Wang, B. Seo, X. Luo, Q. Hu, J. Jones, M. Zeller, K. Wang, B. M. Savoie, K. Zhao, L. Dou**

Contents

1. Experimental Procedures 2
1.1. General Information 2
1.2. Materials Synthesis 3
1.3. Single Crystal Preparation 13
1.4. Topochemical Polymerization Process 13
1.5. DFT Calculations 13
1.6. Polymer Thin Film Processing 14
1.7. Polymer sample preparation for tensile stress-strain tests 14
1.8. Summary of ^{1}H and ^{13}C NMR for BIT Monomers 15
1.9. Cambridge Crystallographic Data Center Reference Numbers for Crystals 28
2. Results and Discussions 29
3. References 69
4. Author Contributions 69

1. Experimental Procedures

1.1. General Information

All reagents were purchased from suppliers including Fisher Scientific, Sigma-Aldrich, VWR, and Enamine and used without further purification. 1H- and 13C-NMR spectra were recorded using a Bruker ARX 400 spectrometer where the samples were dissolved in deuterated chloroform at 298 K. Single crystal absorption spectra were recorded on an Agilent UV-Vis-NIR Cary-5000 spectrometer in transmission mode. Single crystals were analyzed using a Bruker Quest diffractometer with kappa geometry, an I-μ-S microsource X-ray tube (Cu Kα radiation, λ = 1.54178 Å), laterally graded multilayer (Goebel) mirror for monochromatization, and a Photon2 CMOS area detector. The instrument is equipped with Oxford Cryosystems low temperature device. The examination and data collection were performed at 150 K. Nanoindentation experiments were performed using a KLA G200 Nano-indenter in an argon filled glovebox. Modulus were obtained as the average value for tip penetration greater than 150 nm. Ultrasonication of polymer single crystals was conducted using QSonica Q700 Sonicatior with a ½'' standard probe. Heat press was conducted using Dulytek Elite DE10K Electric Hybrid Rosin Press. Tensile stress-strain tests were conducted using Mechanical Testing System (MTS) Criterion Model 43. Scanning electron microscope (SEM) was performed using Quanta 3D FEG.

For all crystal structures, without specific labels, atoms are labeled in the following colors: C, grey; O, red; S, yellow; H, white; Br, brown; Cl, green.

1.2. Materials Synthesis

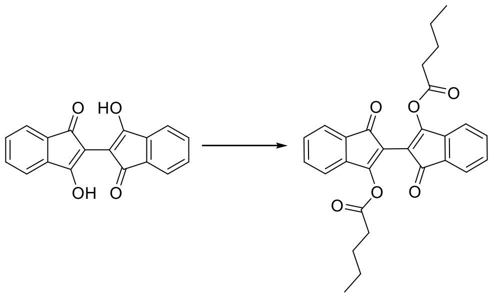

1,1'-dioxo-1H,1'H-[2,2'-biindene]-3,3'-diyl dipentanoate (**BIT-5**):

[2,2′-bi-1H-indene]-3,3′- dihydroxy-1,1′-dione (BIT-OH_2, 1.00 g, 3.4 mmol) was added to a dry two-neck round-bottom flask, followed by addition of 40 mL anhydrous chloroform under argon atmosphere. The mixture was cooled to -15 °C in salt ice bath and 0.96g (7.6mmol) N,N-Diisopropylethylamine was added. BIT-OH_2 was dissolved, and a purple solution was formed. Valeroyl chloride (0.81 mL, 6.8 mmol) was added dropwise in ten minutes to the solution at -15 °C. The solution gradually turned orange during the 2 hours reaction at -15 °C and was then quenched by water. After washed with brine and dried with MgSO4, the crude product was dried via rotary evaporation and recrystallized by adding 5mL of cold methanol into the flask. Orange powder of BIT-5 was filtered (1.18 g, 75% yield). ^{1}H NMR (400 MHz, CDCl3) δ 7.43 (d, J = 7.1 Hz, 1H), 7.37 (td, J = 7.6, 1.2 Hz, 1H), 7.27 (d, J = 7.2 Hz, 1H), 7.09 (d, J = 7.2 Hz, 1H), 2.65 (t, J = 7.5 Hz, 2H), 1.72 (p, J = 7.5 Hz, 2H), 1.43 (h, J = 7.4 Hz, 2H), 0.95 (t, J = 7.3 Hz, 3H). ^{13}C NMR (101 MHz, CDCl3) δ 192.25, 169.10, 164.32, 140.26, 133.27, 130.76, 129.81, 122.18, 119.80, 112.49, 33.78, 26.39, 22.02, 13.58. HRMS (ESI+) calculated for $C_{28}H_{26}O_6$ ($[M+H]^+$): 459.1808; found: 459.1796.

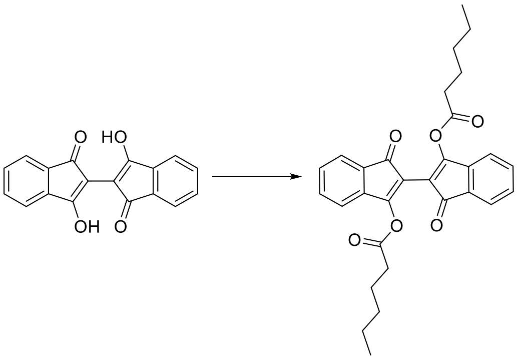

1,1'-dioxo-1H,1'H-[2,2'-biindene]-3,3'-diyl dihexanoate (**BIT-6**):

[2,2′-bi-1H-indene]-3,3′- dihydroxy-1,1′-dione (BIT-OH_2, 1.00 g, 3.4 mmol) was added to a dry two-neck round-bottom flask, followed by addition of 40 mL anhydrous chloroform under argon atmosphere. The mixture was cooled to -15 °C in salt ice bath and 0.96g (7.6mmol) N,N-Diisopropylethylamine was added. BIT-OH_2 was dissolved, and a purple solution was formed. Hexanoyl chloride (0.95 mL, 6.8 mmol) was added dropwise in ten minutes to the solution at -15 °C. The solution gradually turned orange during the 2 hours reaction at -15 °C and was then quenched by water. After washed with brine and dried with MgSO4, the crude product was dried via rotary evaporation and recrystallized by adding 5mL of cold methanol into the flask. Orange powder of BIT-6 was filtered (1.24 g, 75% yield). ^{1}H NMR (400 MHz, $CDCl_3$) δ 7.45 – 7.40 (m, 1H), 7.37 (td, J = 7.6, 1.2 Hz, 1H), 7.28 (d, J = 7.3 Hz, 1H), 7.10 (d, J = 7.1

Hz, 1H), 2.65 (t, J = 7.4 Hz, 2H), 1.73 (pd, J = 5.9, 3.0 Hz, 2H), 1.45 – 1.27 (m, 4H), 0.95 – 0.87 (m, 3H). ^{13}C NMR (100 MHz, $CDCl_3$, ppm) δ: 192.26, 169.09, 164.26, 140.27, 133.26, 130.74, 129.79, 122.18, 119.78, 112.46, 34.03, 31.02, 24.04, 22.22, 13.80. HRMS (ESI+) calculated for $C_{30}H_{30}O_6Na^+$ ($[M+Na]^+$): 509.1935; found: 509.1930.

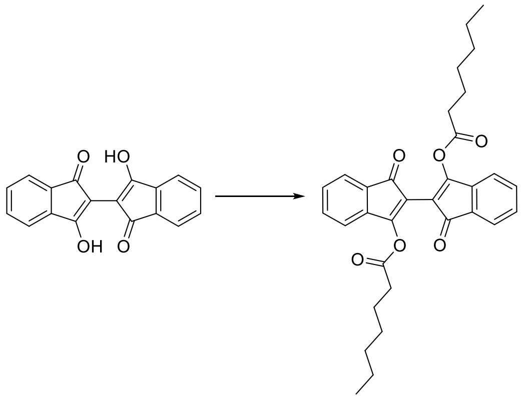

1,1'-dioxo-1H,1'H-[2,2'-biindene]-3,3'-diyl diheptanoate (**BIT-7**):

[2,2′-bi-1H-indene]-3,3′- dihydroxy-1,1′-dione ($BIT\text{-}OH_2$, 1.00 g, 3.4 mmol) was added to a dry two-neck round-bottom flask, followed by addition of 40 mL anhydrous chloroform under argon atmosphere. The mixture was cooled to -15 °C in salt ice bath and 0.96g (7.6mmol) N,N-Diisopropylethylamine was added. $BIT\text{-}OH_2$ was dissolved, and a purple solution was formed. Heptanoyl chloride (1.05 mL, 6.8 mmol) was added dropwise in ten minutes to the solution at -15 °C. The solution gradually turned orange during the 2 hours reaction at -15 °C and was then quenched by water. After washed with brine and dried with MgSO4, the crude product was dried via rotary evaporation and recrystallized by adding 5mL of cold methanol into the flask. Orange powder of BIT-7 was filtered (1.32 g, 75% yield). ^{1}H NMR (400 MHz, $CDCl_3$) δ 7.43 (d, J = 7.1 Hz, 1H), 7.37 (t, J = 7.5 Hz, 1H), 7.28 (d, J = 7.4 Hz, 1H), 7.10 (d, J = 7.1 Hz, 1H), 2.65 (t, J = 7.4 Hz, 2H), 1.72 (p, J = 7.3 Hz, 2H), 1.45 – 1.21 (m, 6H), 0.89 (d, J = 6.3 Hz, 3H). ^{13}C NMR (101 MHz, $CDCl_3$) δ 140.27, 133.27, 130.75, 129.80, 122.20, 119.80, 77.11, 34.08, 31.34, 28.57, 24.32, 22.38, 13.93.

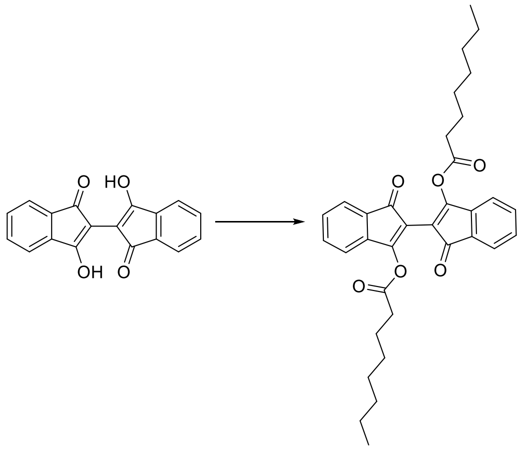

1,1'-dioxo-1H,1'H-[2,2'-biindene]-3,3'-diyl dioctanoate (**BIT-8**):

[2,2′-bi-1H-indene]-3,3′- dihydroxy-1,1′-dione ($BIT\text{-}OH_2$, 1.00 g, 3.4 mmol) was added to a dry two-neck round-bottom flask, followed by addition of 40 mL anhydrous chloroform under argon atmosphere. The mixture was cooled to -15 °C in salt ice bath and 0.96g (7.6mmol) N,N-Diisopropylethylamine was added. $BIT\text{-}OH_2$ was dissolved, and a purple solution was formed. Octanoyl chloride (1.29 mL, 6.8 mmol) was added dropwise in ten minutes to the solution at -15 °C. The solution gradually turned orange during the 2

hours reaction at -15 °C and was then quenched by water. After washed with brine and dried with MgSO4, the crude product was dried via rotary evaporation and recrystallized by adding 5mL of cold methanol into the flask. Orange powder of BIT-8 was filtered (1.38 g, 75% yield). ^{1}H NMR (400 MHz, $CDCl_3$) δ 7.43 (dd, J = 7.0, 0.9 Hz, 1H), 7.37 (td, J = 7.6, 1.2 Hz, 1H), 7.27 (d, J = 7.3 Hz, 1H), 7.10 (d, J = 7.1 Hz, 1H), 2.65 (t, J = 7.4 Hz, 2H), 1.72 (p, J = 7.4 Hz, 2H), 1.39 – 1.22 (m, 8H), 0.91 – 0.83 (m, 3H). ^{13}C NMR (101 MHz, $CDCl_3$) δ 192.27, 169.11, 164.31, 140.28, 133.26, 130.75, 129.79, 122.20, 119.79, 112.48, 34.08, 31.55, 28.86, 28.83, 24.37, 22.49, 13.97 HRMS (ESI) calculated for $C_{34}H_{38}O_6Na^+$ ($[M+Na]^+$): 565.2561; found: 565.2555.

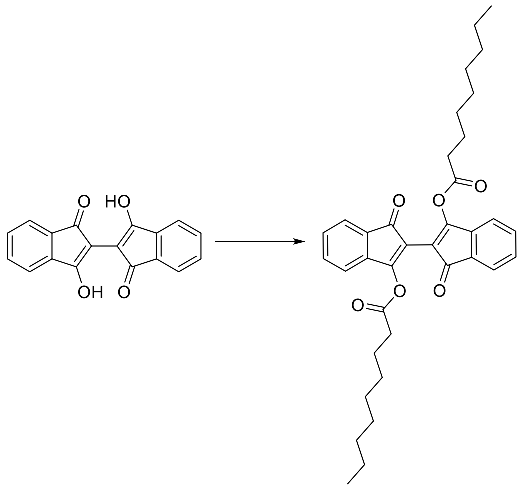

1,1'-dioxo-1H,1'H-[2,2'-biindene]-3,3'-diyl dinonanoate (**BIT-9**):

[2,2′-bi-1H-indene]-3,3′- dihydroxy-1,1′-dione ($BIT-OH_2$, 1.00 g, 3.4 mmol) was added to a dry two-neck round-bottom flask, followed by addition of 40 mL anhydrous chloroform under argon atmosphere. The mixture was cooled to -15 °C in salt ice bath and 0.96g (7.6mmol) N,N-Diisopropylethylamine was added. $BIT-OH_2$ was dissolved, and a purple solution was formed. Nonanoyl chloride (1.23 mL, 6.8 mmol) was added dropwise in ten minutes to the solution at -15 °C. The solution gradually turned orange during the 2 hours reaction at -15 °C and was then quenched by water. After washed with brine and dried with MgSO4, the crude product was dried via rotary evaporation and recrystallized by adding 5mL of cold methanol into the flask. Orange powder of BIT-9 was filtered (1.46 g, 75% yield). ^{1}H NMR (400 MHz, $CDCl_3$) δ 7.43 (d, J = 7.2 Hz, 1H), 7.37 (t, J = 7.5 Hz, 1H), 7.27 (d, J = 10.5 Hz, 1H), 7.10 (d, J = 7.2 Hz, 1H), 2.64 (t, J = 7.4 Hz, 2H), 1.78 – 1.66 (m, 1H), 1.39 (t, J = 7.8 Hz, 2H), 1.31 (dd, J = 13.6, 6.4 Hz, 4H), 1.26 (d, J = 4.2 Hz, 4H), 0.88 (t, J = 6.5 Hz, 3H). ^{13}C NMR (101 MHz, $CDCl_3$) δ 192.28, 169.12, 164.29, 140.28, 133.27, 130.75, 129.79, 122.20, 119.80, 112.48, 77.11, 34.08, 31.70, 29.13, 29.02, 28.90, 24.36, 22.55, 14.00.

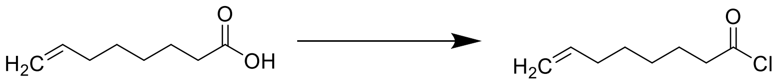

Oct-7-enoyl chloride:

In a two-neck round-bottom flask, oxalyl chloride (2.32 g, 18.28 mmol) was dissolved in 4 mL of anhydrous chloroform. Oct-enoic acid (2.00g, 14.06 mmol) was dissolved in 4 mL of anhydrous chloroform and was added dropwise to the previous solution. After the addition, five drops of DMF were added and the mixture was stirred at room temperature for 30 minutes. Then Et_2O was added, and the mixture was filtered and concentrated under reduced pressure to give oct-7-enoyl chloride (2.15g, 95% yield). ^{1}H NMR (400 MHz,

$CDCl_3$) δ 5.78 (ttd, *J* = 16.9, 6.5, 1.8 Hz, 1H), 5.09 – 4.88 (m, 2H), 2.99 – 2.78 (m, 2H), 2.05 (tt, *J* = 6.8, 3.4 Hz, 2H), 1.80 – 1.64 (m, 2H), 1.39 (tq, *J* = 14.4, 4.9 Hz, 4H).

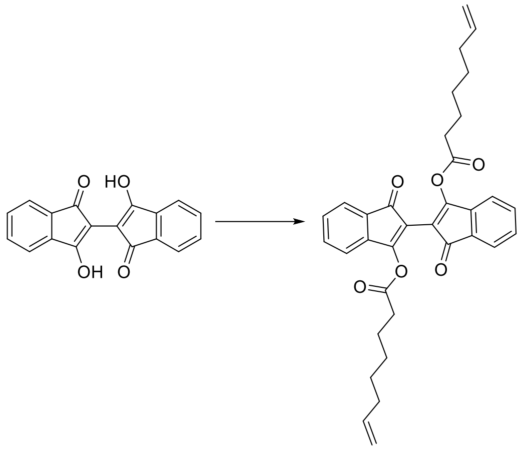

1,1'-dioxo-1H,1'H-[2,2'-biindene]-3,3'-diyl bis(oct-7-enoate) (**BIT-8D**):

[2,2′-bi-1H-indene]-3,3′- dihydroxy-1,1′-dione (BIT-OH_2, 1.00 g, 3.4 mmol) was added to a dry two-neck round-bottom flask, followed by addition of 40 mL anhydrous chloroform under argon atmosphere. The mixture was cooled to -15 °C in salt ice bath and 0.96g (7.6mmol) N,N-Diisopropylethylamine was added. BIT-OH_2 was dissolved, and a purple solution was formed. Oct-7-enoyl chloride (1.20 g, 7.5 mmol) was added dropwise in ten minutes to the solution at -15 °C. The solution gradually turned orange during the 2 hours reaction at -15 °C and was then quenched by water. After washed with brine and dried with MgSO4, the crude product was dried via rotary evaporation and recrystallized by adding 5mL of cold methanol into the flask. Orange powder of BIT-8D was filtered (1.37 g, 75% yield). ^1H NMR (400 MHz, $CDCl_3$) δ 7.43 (dd, *J* = 7.1, 1.0 Hz, 1H), 7.37 (td, *J* = 7.6, 1.3 Hz, 1H), 7.28 (d, *J* = 7.2 Hz, 1H), 7.10 (d, *J* = 7.0 Hz, 1H), 5.79 (ddt, *J* = 17.0, 10.2, 6.7 Hz, 1H), 5.05 – 4.90 (m, 2H), 2.65 (t, *J* = 7.4 Hz, 2H), 2.05 (tdd, *J* = 7.0, 5.5, 2.6 Hz, 2H), 1.73 (tt, *J* = 7.5, 5.9 Hz, 2H), 1.48 – 1.36 (m, 4H). ^{13}C NMR (101 MHz, $CDCl_3$) δ 192.30, 169.05, 164.25, 140.26, 138.63, 133.29, 130.73, 129.82, 122.21, 119.81, 114.41, 112.46, 34.00, 33.43, 28.41, 28.33, 24.19. HRMS (ESI) calculated for $C_{34}H_{34}O_6Na^+$ ([M+Na]$^+$): 561.2248; found: 561.2242.

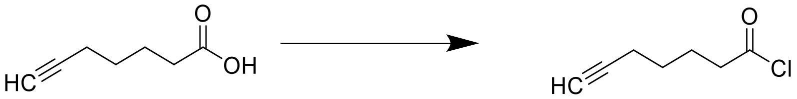

Hept-6-ynoyl chloride:

In a two-neck round-bottom flask, thionyl chloride (1.84 g, 15.48 mmol) was dissolved in 4 mL of anhydrous chloroform. Hept-6-ynoic acid (1.50g, 11.90 mmol) was dissolved in 4 mL of anhydrous chloroform and was added dropwise to the previous solution. After the addition, five drops of DMF were added and the mixture was stirred at room temperature for 3 hours. Then the mixture concentrated under reduced pressure to give hept-6-ynoyl chloride (2.05g, 92% yield). ^1H NMR (400 MHz, $CDCl_3$) δ 2.90 (td, *J* = 7.3, 2.5 Hz, 2H), 2.20 (tt, *J* = 6.6, 2.9 Hz, 2H), 1.95 (q, *J* = 2.6 Hz, 1H), 1.81 (ddt, *J* = 15.1, 10.3, 5.6 Hz, 2H), 1.55 (tq, *J* = 9.5, 5.3 Hz, 2H).

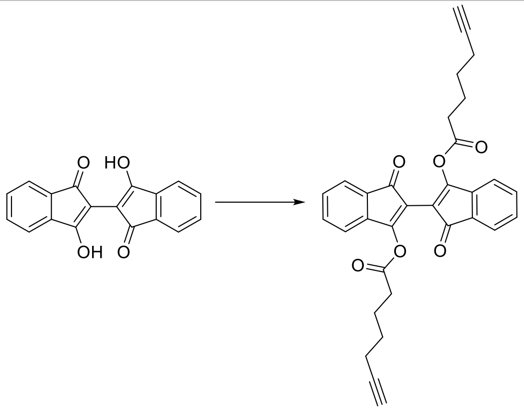

1,1'-dioxo-1H,1'H-[2,2'-biindene]-3,3'-diyl bis(hept-6-ynoate) (**BIT-7T**):

[2,2′-bi-1H-indene]-3,3′- dihydroxy-1,1′-dione (BIT-OH_2, 1.00 g, 3.4 mmol) was added to a dry two-neck round-bottom flask, followed by addition of 40 mL anhydrous chloroform under argon atmosphere. The mixture was cooled to -15 °C in salt ice bath and 0.96g (7.6mmol) N,N-Diisopropylethylamine was added. BIT-OH_2 was dissolved, and a purple solution was formed. Hept-6-ynoyl chloride (1.08 g, 7.5 mmol) was added dropwise in ten minutes to the solution at -15 °C. The solution gradually turned orange during the 2 hours reaction at -15 °C and was then quenched by water. After washed with brine and dried with MgSO4, the crude product was dried via rotary evaporation and recrystallized by adding 5mL of cold methanol into the flask. Orange powder of BIT-7T was filtered (1.31 g, 75% yield). ^{1}H NMR (400 MHz, $CDCl_3$) δ 7.47 – 7.33 (m, 2H), 7.27 (ddd, *J* = 7.9, 7.1, 1.0 Hz, 2H), 7.11 (dt, *J* = 7.3, 0.9 Hz, 1H), 2.69 (t, *J* = 7.3 Hz, 2H), 2.24 (td, *J* = 7.0, 2.7 Hz, 2H), 1.95 (t, *J* = 2.7 Hz, 1H), 1.92 – 1.80 (m, 2H), 1.71 – 1.57 (m, 2H). ^{13}C NMR (101 MHz, $CDCl_3$) δ 192.38, 168.73, 164.07, 140.22, 133.35, 130.66, 129.89, 122.27, 119.82, 112.39, 83.70, 68.67, 33.46, 27.46, 23.34, 18.02. HR-MS (ESI+) Expected 507.1808 [M]+ Observed 507.1794.

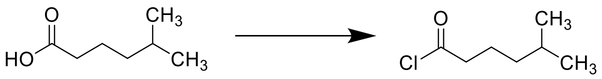

5-methylhexanoyl chloride:

In a two-neck round-bottom flask, thionyl chloride (1.20 mL, 16.30 mmol) was added under argon atmosphere. 5-methylhexanoic acid (2.02g, 15.54 mmol) was added dropwise to the flask. The mixture was heated under reflux for 2.5h. Then the mixture was cooled to room temperature and extra thionyl chloride was removed under reduced pressure to give a colorless liquid (2.26g, 98% yield). ^{1}H NMR (400 MHz, $CDCl_3$) δ 2.84 (t, *J* = 7.2 Hz, 2H), 1.75 – 1.67 (m, 1H), 1.66 (t, *J* = 7.4 Hz, 1H), 1.54 (hept, *J* = 7.1 Hz, 1H), 1.21 (q, *J* = 7.5 Hz, 2H), 0.90 – 0.84 (m, 6H).

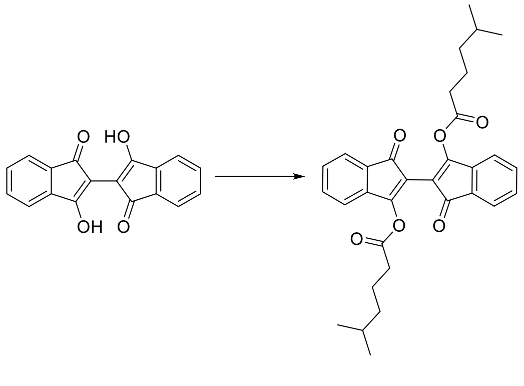

1,1'-dioxo-1H,1'H-[2,2'-biindene]-3,3'-diyl bis(5-methylhexanoate) (**BIT-6-Me**):

To a two-neck round-bottom flask, [2,2′-bi-1H-indene]-3,3′- dihydroxy-1,1′-dione (BIT-OH_2, 0.40 g, 1.37 mmol) was added in 20 mL dry chloroform under argon atmosphere. The mixture was cooled to -15 °C in salt ice bath and 0.52 mL N,N-Diisopropylethylamine was added. BIT-OH_2 dissolved, and a purple solution formed. 5-methylhexanoyl chloride (0.51 g, 3.49 mmol) was added dropwise to the solution at -15 °C. The solution gradually turned orange during the 2 hours reaction at -15 °C and was then quenched by water. After washed with brine and dried with $MgSO_4$, the crude product was dried via rotary evaporation and recrystallized by adding 5mL of cold methanol into the flask. Orange powder of BIT-6-Me was filtered (0.56g, 80% yield). ^{1}H NMR (400 MHz, $CDCl_3$) δ 7.46 – 7.33 (m, 2H), 7.31 – 7.22 (m, 1H), 7.10 (d, *J* = 7.2 Hz, 1H), 2.63 (t, *J* = 7.5 Hz, 2H), 1.79 – 1.66 (m, 2H), 1.66 – 1.49 (m, *J* = 6.4 Hz, 1H), 1.33 – 1.22 (m, 2H), 0.89 (d, *J* = 6.6 Hz, 6H). ^{13}C NMR (101 MHz, $CDCl_3$) δ 192.28, 169.11, 164.28, 140.28, 133.28, 130.75, 129.80, 122.21, 119.80, 112.46, 38.03, 34.28, 27.67, 22.36, 22.26. HRMS (ESI) calculated for $C_{32}H_{34}O_6Na^+$ ($[M+Na]^+$): 537.2248; found: 537.2241.

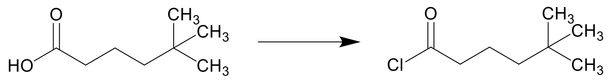

5,5-dimethylhexanoyl chloride:

In a two-neck round-bottom flask, thionyl chloride (1.20 mL, 16.30 mmol) was added under argon atmosphere. 5,5-dimethylhexanoic acid (2.23g, 15.52 mmol) was added dropwise to the flask. The mixture was heated under reflux for 2.5h. Then the mixture was cooled to room temperature and extra thionyl chloride was removed under reduced pressure to give a colorless liquid (2.34g, 93% yield). ^{1}H NMR (400 MHz, $CDCl_3$) δ 2.86 (t, *J* = 7.2 Hz, 2H), 2.17 (s, 1H), 1.74 – 1.64 (m, 2H), 1.27 – 1.17 (m, 2H), 0.89 (s, 9H).

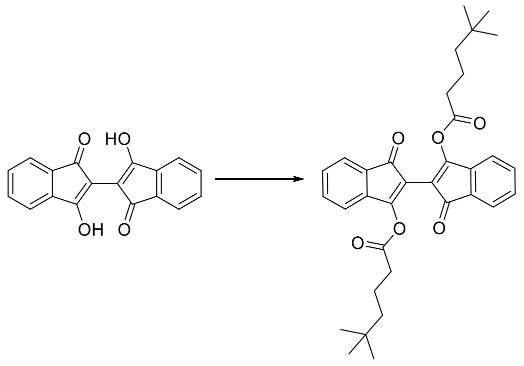

1,1'-dioxo-1H,1'H-[2,2'-biindene]-3,3'-diyl bis(5,5-dimethylhexanoate) (**BIT-6-2Me**):

[2,2′-bi-1H-indene]-3,3′- dihydroxy-1,1′-dione (BIT-OH_2, 1.00 g, 3.4 mmol) was added to a dry two-neck round-bottom flask, followed by addition of 40 mL anhydrous chloroform under argon atmosphere. The mixture was cooled to -15 °C in salt ice bath and 0.96g (7.6mmol) N,N-Diisopropylethylamine was added. BIT-OH_2 was dissolved, and a purple solution was formed. 5,5-dimethylhexanoyl chloride (1.38 g, 8.5 mmol) was added dropwise in ten minutes to the solution at -15 °C. The solution gradually turned orange during the 2 hours reaction at -15 °C and was then quenched by water. After washed with brine and dried with MgSO4, the crude product was dried via rotary evaporation and recrystallized by adding 5mL of cold methanol into the flask. Orange powder of BIT-6-2Me was filtered (1.44 g, 78% yield). ^{1}H NMR (400 MHz, $CDCl_3$) δ 7.43 (dt, *J* = 7.1, 0.8 Hz, 1H), 7.37 (td, *J* = 7.5, 1.1 Hz, 1H), 7.30 – 7.25 (m, 1H), 7.10 (dd, *J* = 7.2, 1.0 Hz, 1H), 2.63 (t, *J* = 7.4 Hz, 2H), 1.75 – 1.65 (m, 2H), 1.31 – 1.24 (m, 2H), 0.89 (s, 9H). ^{13}C NMR

(101 MHz, $CDCl_3$) δ 192.25, 169.12, 164.34, 140.27, 133.28, 130.75, 129.80, 122.21, 119.82, 112.47, 43.28, 34.86, 30.25, 29.18, 19.74. HR-MS (ESI+) Expected 543.2747 [M]+ Observed 543.2757.

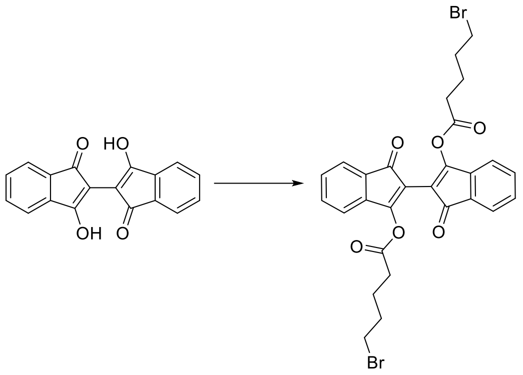

1,1'-dioxo-1H,1'H-[2,2'-biindene]-3,3'-diyl bis(5-bromopentanoate) (**BIT-5-Br**):

[2,2′-bi-1H-indene]-3,3′- dihydroxy-1,1′-dione (BIT-OH_2, 1.00 g, 3.4 mmol) was added to a dry two-neck round-bottom flask, followed by addition of 40 mL anhydrous chloroform under argon atmosphere. The mixture was cooled to -15 °C in salt ice bath and 0.96g (7.6mmol) N,N-Diisopropylethylamine was added. BIT-OH_2 was dissolved, and a purple solution was formed. 5-Bromopentanoyl chloride (0.91 mL, 6.8 mmol) was added dropwise in ten minutes to the solution at -15 °C. The solution gradually turned orange during the 2 hours reaction at -15 °C and was then quenched by water. After washed with brine and dried with MgSO4, the crude product was dried via rotary evaporation and recrystallized by adding 5mL of cold methanol into the flask. Orange powder of BIT-5-Br was filtered (1.65 g, 79% yield). ^{1}H NMR (400 MHz, $CDCl_3$) δ 7.43 (d, J = 7.1 Hz, 1H), 7.38 (t, J = 7.3 Hz, 1H), 7.29 (d, J = 7.5 Hz, 1H), 7.11 (d, J = 7.2 Hz, 1H), 3.43 (t, J = 6.5 Hz, 2H), 2.71 (t, J = 7.0 Hz, 2H), 1.98 (tdd, J = 7.9, 6.5, 3.8 Hz, 2H), 1.94 – 1.84 (m, 2H). ^{13}C NMR (101 MHz, $CDCl_3$) δ 192.47, 168.53, 163.92, 140.18, 133.43, 130.59, 129.97, 122.31, 119.83, 112.33, 32.98, 32.81, 31.53, 22.84. HR-MS (ESI+) Expected 615.0018 [M]+ Observed 615.0040.

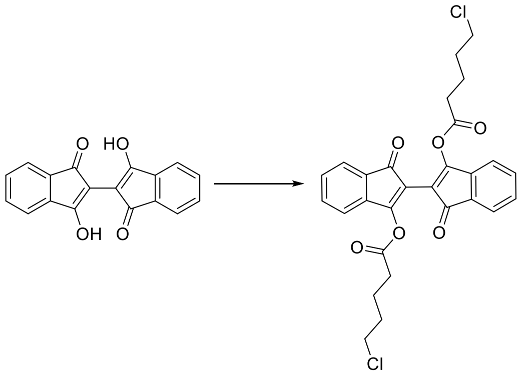

1,1'-dioxo-1H,1'H-[2,2'-biindene]-3,3'-diyl bis(5-chloropentanoate) (**BIT-5-Cl**):

[2,2′-bi-1H-indene]-3,3′- dihydroxy-1,1′-dione (BIT-OH_2, 1.00 g, 3.4 mmol) was added to a dry two-neck round-bottom flask, followed by addition of 40 mL anhydrous chloroform under argon atmosphere. The mixture was cooled to -15 °C in salt ice bath and 0.96g (7.6mmol) N,N-Diisopropylethylamine was added. BIT-OH_2 was dissolved, and a purple solution was formed. 5-Chloropentanoyl chloride (1.05 g, 6.8 mmol) was added dropwise in ten minutes to the solution at -15 °C. The solution gradually turned orange during the 2 hours reaction at -15 °C and was then quenched by water. After washed with brine and dried with MgSO4, the crude product was dried via rotary evaporation and recrystallized by adding 5mL of cold methanol into the flask. Orange powder of BIT-5-Cl was filtered

(1.40 g, 78% yield). ^{1}H NMR (400 MHz, $CDCl_3$) δ 7.43 (d, *J* = 7.1 Hz, 1H), 7.38 (t, *J* = 7.3 Hz, 1H), 7.29 (d, *J* = 7.5 Hz, 1H), 7.11 (d, *J* = 7.2 Hz, 1H), 3.43 (t, *J* = 6.5 Hz, 2H), 2.71 (t, *J* = 7.0 Hz, 2H), 1.98 (tdd, *J* = 7.9, 6.5, 3.8 Hz, 2H), 1.94 – 1.84 (m, 2H). ^{13}C NMR (101 MHz, $CDCl_3$) δ 192.47, 168.53, 163.92, 140.18, 133.43, 130.59, 129.97, 122.31, 119.83, 112.33, 32.98, 32.81, 31.53, 22.84. ^{1}H NMR (400 MHz, $CDCl_3$) δ 7.42 (t, *J* = 6.9 Hz, 1H), 7.37 (d, *J* = 7.3 Hz, 1H), 7.29 (d, *J* = 7.3 Hz, 1H), 7.11 (d, *J* = 7.1 Hz, 1H), 3.56 (d, *J* = 5.7 Hz, 2H), 2.71 (q, *J* = 4.7 Hz, 2H), 1.90 (q, *J* = 3.8 Hz, 4H). ^{13}C NMR (101 MHz, $CDCl_3$) δ 192.46, 168.57, 163.94, 140.18, 133.42, 130.59, 129.96, 122.29, 119.83, 112.33, 44.27, 33.10, 31.41, 21.58.

HR-MS (ESI+) Expected 527.1028 [M]+ Observed 527.1016.

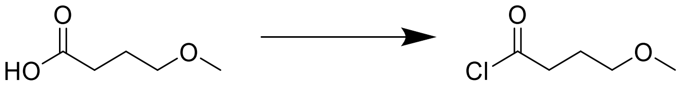

4-methoxybutanoyl chloride:

In a two-neck round-bottom flask, oxalyl chloride (2.10 g, 16.53 mmol) was dissolved in 4 mL of anhydrous chloroform. 4-methoxybutanoic acid (1.50g, 12.71 mmol) was dissolved in 4 mL of anhydrous chloroform and was added dropwise to the previous solution. After the addition, five drops of DMF were added and the mixture was stirred at room temperature for 2h. Then the mixture was concentrated under reduced pressure to give 4-methoxybutanoyl chloride (1.47g, 85% yield). ^{1}H NMR (400 MHz, $CDCl_3$) δ 3.41 (t, *J* = 6.0 Hz, 2H), 3.32 (s, 3H), 3.03 – 2.98 (m, 2H), 2.00 – 1.89 (m, 2H).

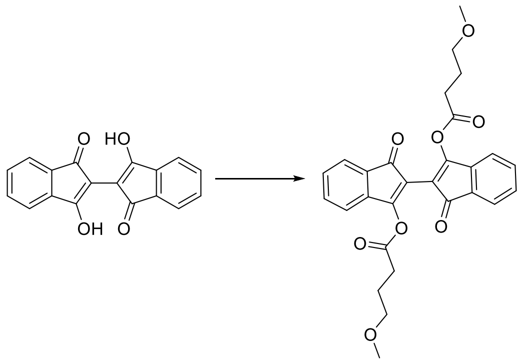

1,1'-dioxo-1H,1'H-[2,2'-biindene]-3,3'-diyl bis(4-methoxybutanoate) (**BIT-6-O**):

[2,2′-bi-1H-indene]-3,3′- dihydroxy-1,1′-dione (BIT-OH_2, 1.00 g, 3.4 mmol) was added to a dry two-neck round-bottom flask, followed by addition of 40 mL anhydrous chloroform under argon atmosphere. The mixture was cooled to -15 °C in salt ice bath and 0.96g (7.6mmol) N,N-Diisopropylethylamine was added. BIT-OH_2 was dissolved, and a purple solution was formed. 4-methoxybutanoyl chloride (1.02 g, 7.5 mmol) was added dropwise in ten minutes to the solution at -15 °C. The solution gradually turned orange during the 2 hours reaction at -15 °C and was then quenched by water. After washed with brine and dried with MgSO4, the crude product was dried via rotary evaporation and recrystallized by adding 5mL of cold methanol into the flask. Orange powder of BIT-6-O was filtered (1.27 g, 75% yield). ^{1}H NMR (400 MHz, $CDCl_3$) δ 7.42 (d, *J* = 6.9 Hz, 1H), 7.37 (td, *J* = 7.5, 1.1 Hz, 1H), 7.29 – 7.26 (m, 1H), 7.12 (d, *J* = 7.3 Hz, 1H), 3.47 (t, *J* = 6.0 Hz, 2H), 3.34 (s, 3H), 2.75 (t, *J* = 7.3 Hz, 2H), 2.04 – 1.96 (m, 2H).

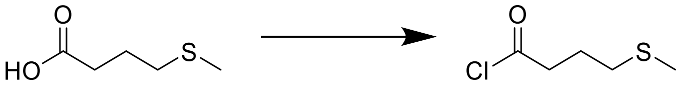

4-(methylthio)butanoyl chloride:

In a two-neck round-bottom flask, oxalyl chloride (1.93 g, 15.20 mmol) was dissolved in 4 mL of anhydrous chloroform. 4-(methylthio)butanoic acid (1.57g, 11.70 mmol) was dissolved in 4 mL of anhydrous chloroform and was added dropwise to the previous solution. After the addition, five drops of DMF were added and the mixture was stirred at room temperature for 2h. Then the mixture was concentrated under reduced pressure to give 4-(methylthio)butanoyl chloride (1.60g, 90% yield). ^{1}H NMR (400 MHz, $CDCl_3$) δ 3.05 (t, *J* = 7.2 Hz, 2H), 2.55 (t, *J* = 6.9 Hz, 2H), 2.09 (s, 3H), 2.00 (p, *J* = 6.9 Hz, 2H).

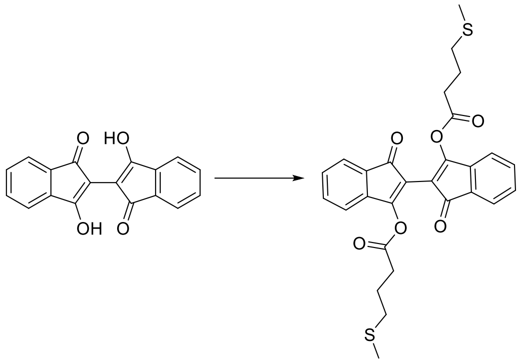

1,1'-dioxo-1*H*,1'*H*-[2,2'-biindene]-3,3'-diyl bis(4-(methylthio)butanoate)

1,1'-dioxo-1H,1'H-[2,2'-biindene]-3,3'-diyl bis(4-(methylthio)butanoate) (**BIT-6-S**):

[2,2′-bi-1H-indene]-3,3′- dihydroxy-1,1′-dione (BIT-OH_2, 1.00 g, 3.4 mmol) was added to a dry two-neck round-bottom flask, followed by addition of 40 mL anhydrous chloroform under argon atmosphere. The mixture was cooled to -15 °C in salt ice bath and 0.96g (7.6mmol) N,N-Diisopropylethylamine was added. BIT-OH_2 was dissolved, and a purple solution was formed. 4-(methylthio)butanoyl chloride (1.14 g, 7.5 mmol) was added dropwise in ten minutes to the solution at -15 °C. The solution gradually turned orange during the 2 hours reaction at -15 °C and was then quenched by water. After washed with brine and dried with MgSO4, the crude product was dried via rotary evaporation and recrystallized by adding 5mL of cold methanol into the flask. Orange powder of BIT-6-S was filtered (1.35 g, 76% yield). ^{1}H NMR (400 MHz, $CDCl_3$) δ 7.45 – 7.40 (m, 1H), 7.38 (td, *J* = 7.6, 1.2 Hz, 1H), 7.30 – 7.26 (m, 1H), 7.12 (d, *J* = 7.2 Hz, 1H), 2.81 (t, *J* = 7.2 Hz, 2H), 2.60 (t, *J* = 7.1 Hz, 2H), 2.09 (s, 3H), 2.02 (p, *J* = 7.1 Hz, 2H). ^{13}C NMR (101 MHz, $CDCl_3$) δ 192.36, 168.66, 164.04, 140.19, 133.39, 130.64, 129.92, 122.25, 119.86, 112.40, 77.14, 33.04, 32.63, 23.38, 15.14. HR-MS (ESI+) Expected 523.1249 [M]+ Observed 523.1265.

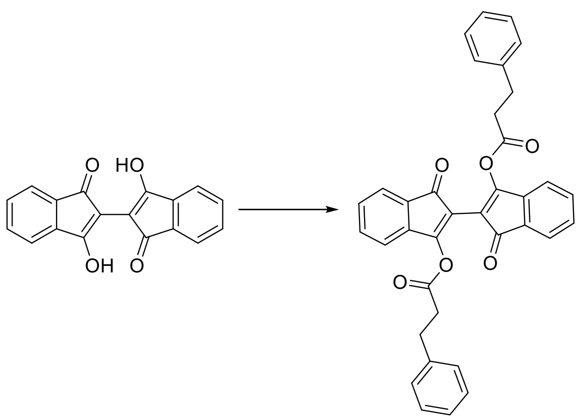

1,1'-dioxo-1H,1'H-[2,2'-biindene]-3,3'-diyl bis(3-phenylpropanoate) (**BIT-4-Ph**):

[2,2′-bi-1H-indene]-3,3′- dihydroxy-1,1′-dione (BIT-OH_2, 1.00 g, 3.4 mmol) was added to a dry two-neck round-bottom flask, followed by addition of 40 mL anhydrous chloroform under argon atmosphere. The mixture was cooled to -15 °C in salt ice bath and 0.96g

(7.6mmol) N,N-Diisopropylethylamine was added. BIT-OH_2 was dissolved, and a purple solution was formed. Hexanoyl chloride (1.476g, 6.8 mmol) was added dropwise in ten minutes to the solution at -15 °C. The solution gradually turned orange during the 2 hours reaction at -15 °C and was then quenched by water. After washed with brine and dried with MgSO4, the crude product was dried via rotary evaporation and recrystallized by adding 5mL of cold methanol into the flask. Orange powder of BIT-4-Ph was filtered (1.41 g, 75% yield). ^{1}H NMR (400 MHz, $CDCl_3$) δ 7.42 (d, J = 7.1 Hz, 1H), 7.40 – 7.34 (m, 1H), 7.28 (dt, J = 11.7, 7.6 Hz, 4H), 7.20 (s, 2H), 7.09 (d, J = 7.2 Hz, 1H), 2.82 – 2.62 (m, 4H), 2.07 (p, J = 7.4 Hz, 2H). ^{13}C NMR (101 MHz, $CDCl_3$) δ 192.31, 168.86, 164.21, 141.03, 140.22, 133.33, 130.71, 129.87, 128.43, 128.37, 126.00, 122.26, 119.84, 112.48, 34.77, 33.34, 25.87. HRMS (ESI+) calculated for $C_{36}H_{26}O_6$ ($[M+H]^+$): 583.2120; found: 583.2116.

1.3. Single Crystal Preparation

All the BIT single crystals were prepared with a slow-evaporation method: 20mg of monomer powders were put in a 20mL vial and first dissolved in 5 mL DCM or chloroform. Then 10 mL of methanol or ethanol was added to the solution and the solution was mixed to a clear orange color. The vial was put in the fume hood with the cap open to let the solvents slowly evaporate from the vial. After the vial was kept in the fume hood overnight, the vial is dry and needle-like (1D polymerizable crystals) or plate-like (2D non-polymerizable crystals) were obtained.

1.4. Topochemical Polymerization Process

Monomer single crystals were first put on the weighing paper on the bench. Then the crystals were put under OLYMPUS BX3M-LEDR optical microscope accessory with distance of 1 cm.

1.5. DFT Calculations

Density Functional Theory (DFT) calculations were used to characterize the difference in lattice energies. All calculations were performed using the Vienna Ab Initio Simulation Package (VASP, version 5.4.1) [1]implemented with projector augmented wave (PAW) methods.[2] The generalized gradient approximation (GGA) by Perdew, Burke, and Ernzerhof (PBE)[3] was used as the exchange-correlation functional with the effective-pairwise dispersion correction of Tkatchenko and Scheffler[4] applied. The plane-wave cut-off for the energy were set as 1000 eV. Initial crystal structures obtained from the experiments were relaxed with the convergence criteria of 10^{-5} eV for the energy and 5×10^{-3} eV·Å^{-1} for the gradient. The k-point mesh utilized was up to $4\times3\times3$ in the gamma centered Monkhorst-Pack Grid generated with vaspkit package.[5] The difference in the lattice energies of crystals with the same molecular compositions were calculated by $\Delta E_{latt} = E_{cryst,\alpha}/Z_{\alpha} - E_{cryst,\beta}/Z_{\beta}$ where $E_{cryst,i}$ and Z_i are the energy and number of molecules in the unit cell i.

1.6. Polymer Thin Film Processing

In a 50 mL beaker, PBIT polymer crystals were suspended in 20 mL chloroform. The mixture was cooled in salt ice bath and the ultrasonic processor probe was immersed into the mixture. The ultrasonic processor parameters were set as amplitude 15, 4 sec pulse and 1 sec rest. After 30-60 minutes sonication, the suspension was filtered with a vacuum filtration apparatus and a nylon membrane filter to provide free-standing polymer thin films. Each thin film is about 50 mg and 100 µm thick. Finally, PBIT polymer thin films were placed between two pieces of aluminum foils and pressed under around 5 MPa pressure at 50 ºC for 3 minutes. After removing the aluminum foils, smooth and robust thin films were obtained.

1.7. Polymer sample preparation for tensile stress-strain tests

Freshly prepared PBIT thin films were cut into roughly 25 mm * 5 mm strips. Then both ends of the polymer strip was glued on pieces of polyethylene terephthalate (PET) sheet with epoxy glue. The initial length for (engineering) strain calculation was measured from the distance between edges of two PET sheets. The cross-sectional area was calculated by the width of the strip multiplied by the thickness of the strip.

1.8. Summary of ^{1}H and ^{13}C NMR for BIT Monomers

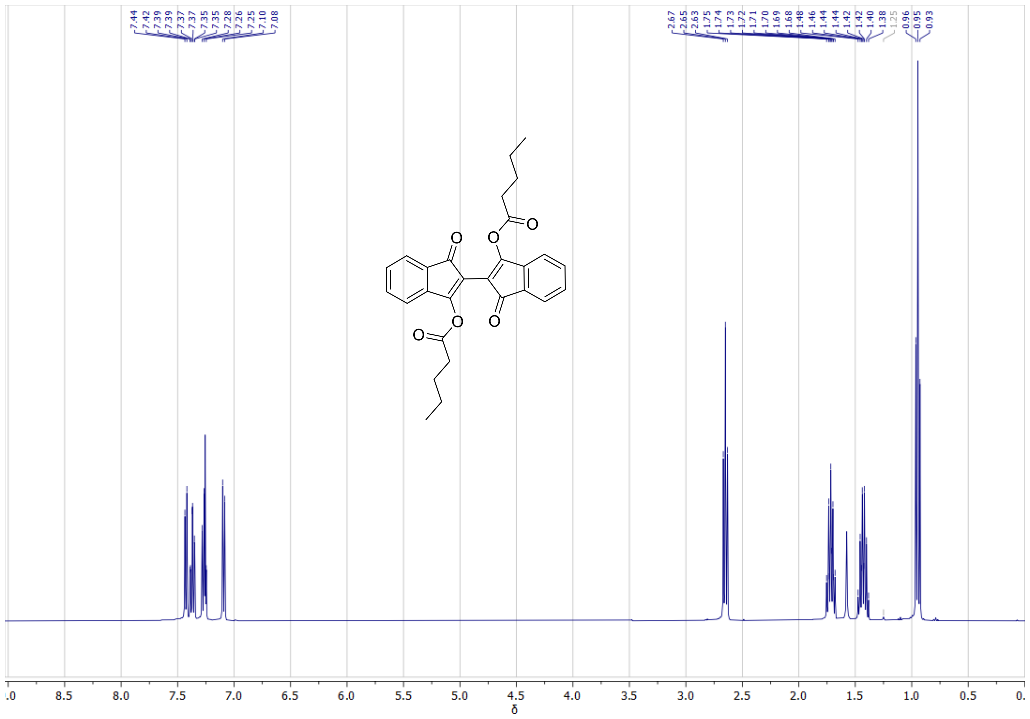

^{1}H NMR spectrum of **BIT-5** in $CDCl_3$

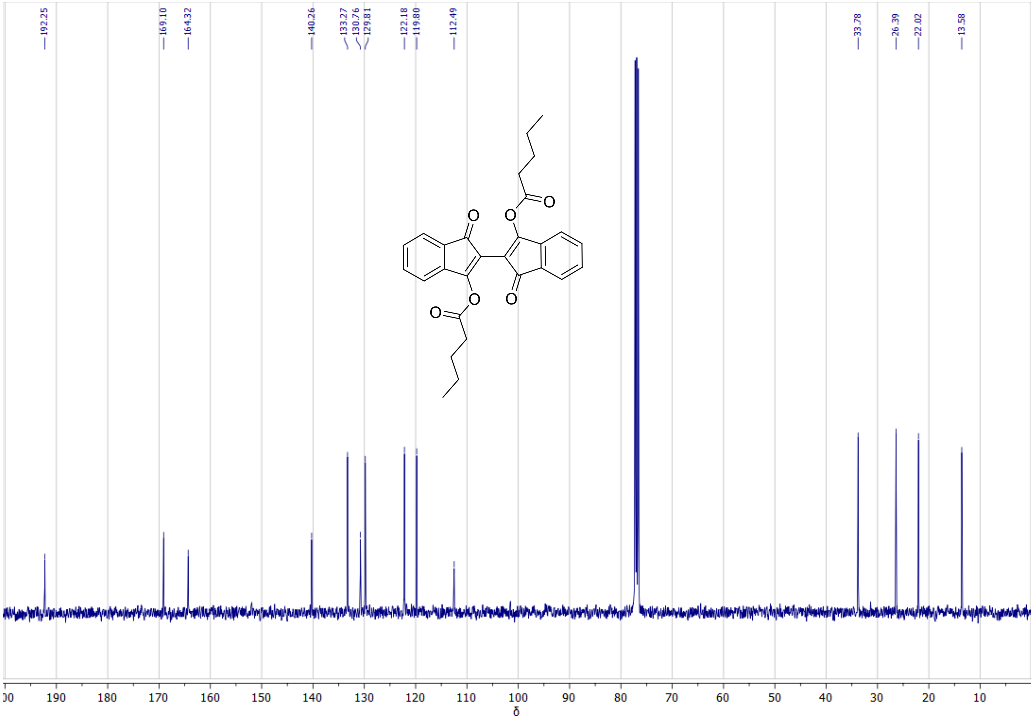

^{13}C NMR spectrum of **BIT-5** in $CDCl_3$

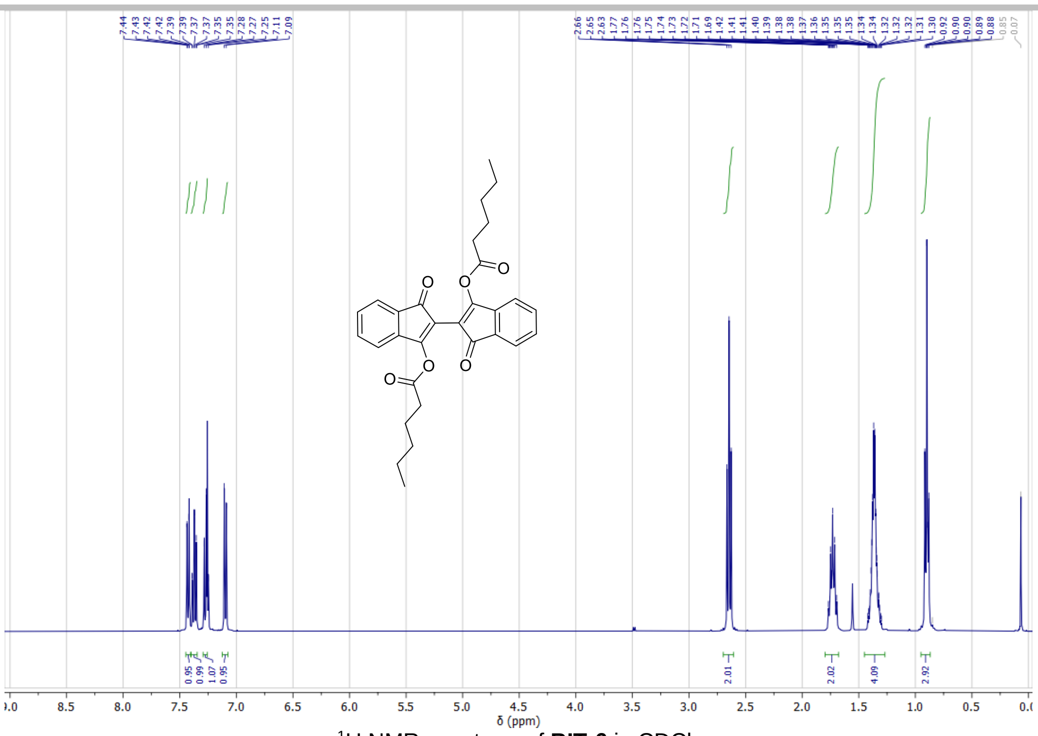

^{1}H NMR spectrum of **BIT-6** in $CDCl_3$

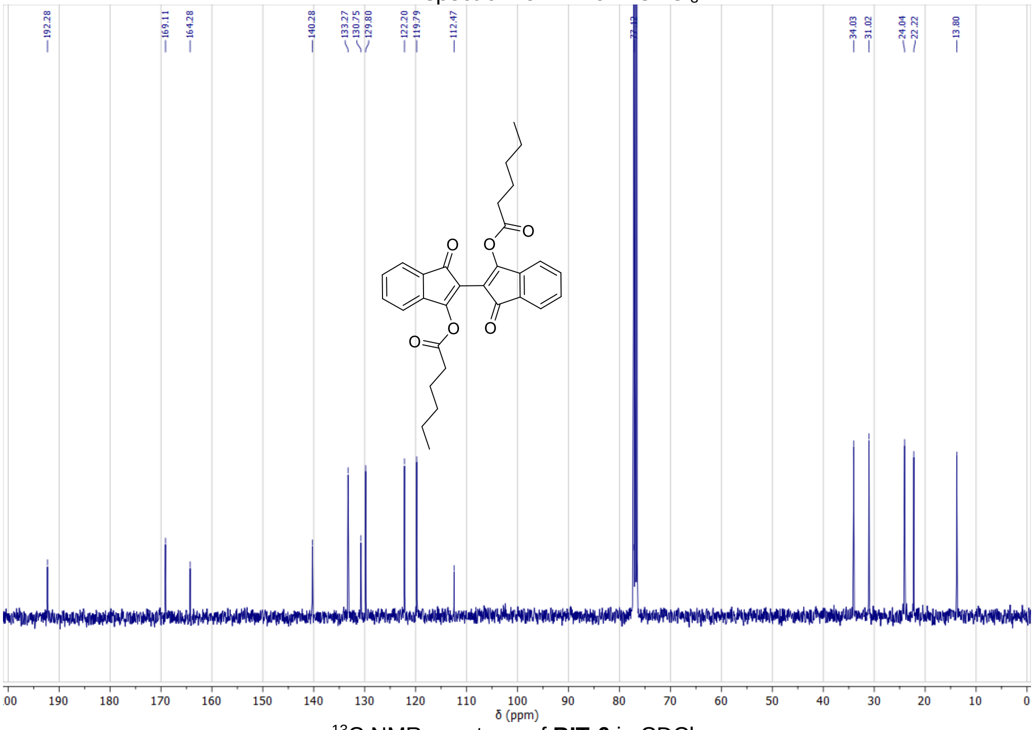

^{13}C NMR spectrum of **BIT-6** in $CDCl_3$

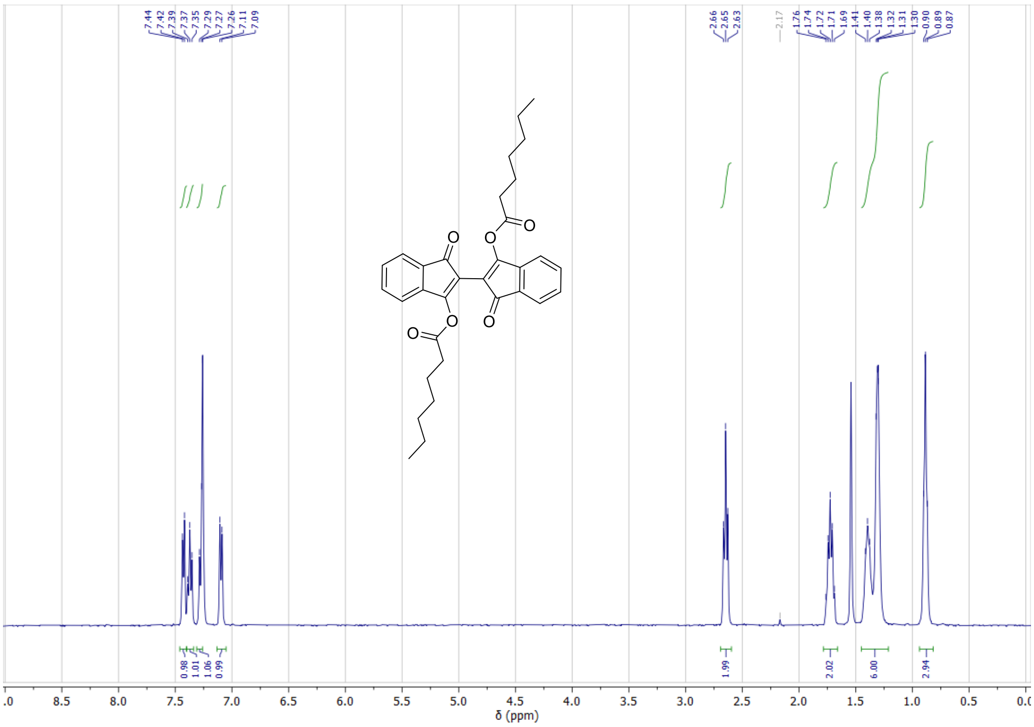

^1H NMR spectrum of **BIT-7** in $CDCl_3$

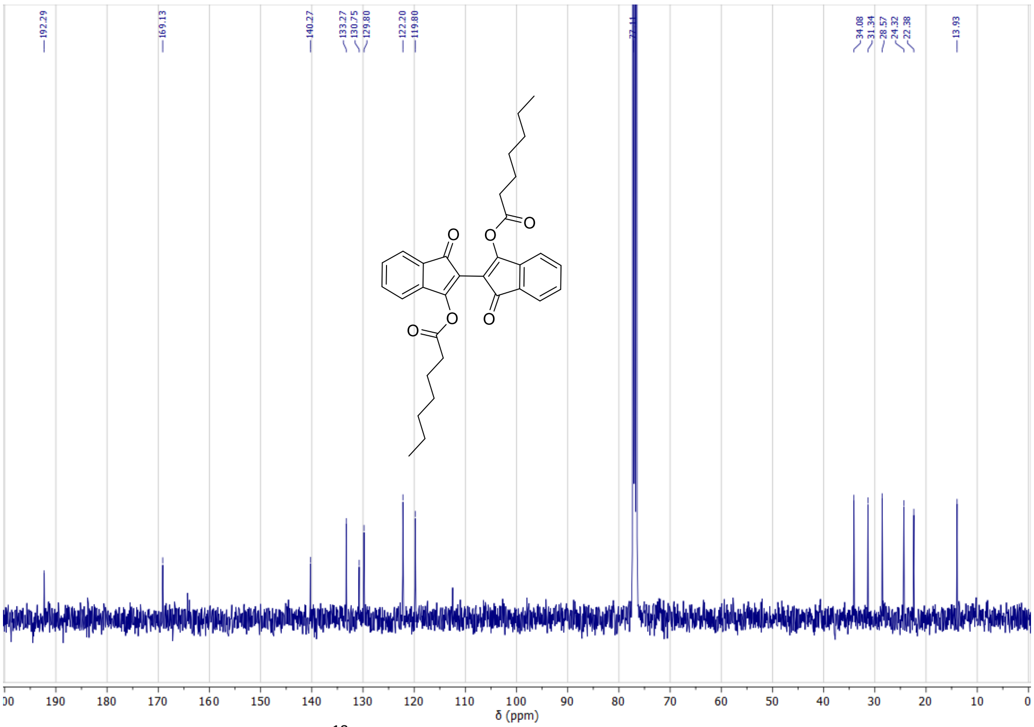

^{13}C NMR spectrum of **BIT-7** in $CDCl_3$

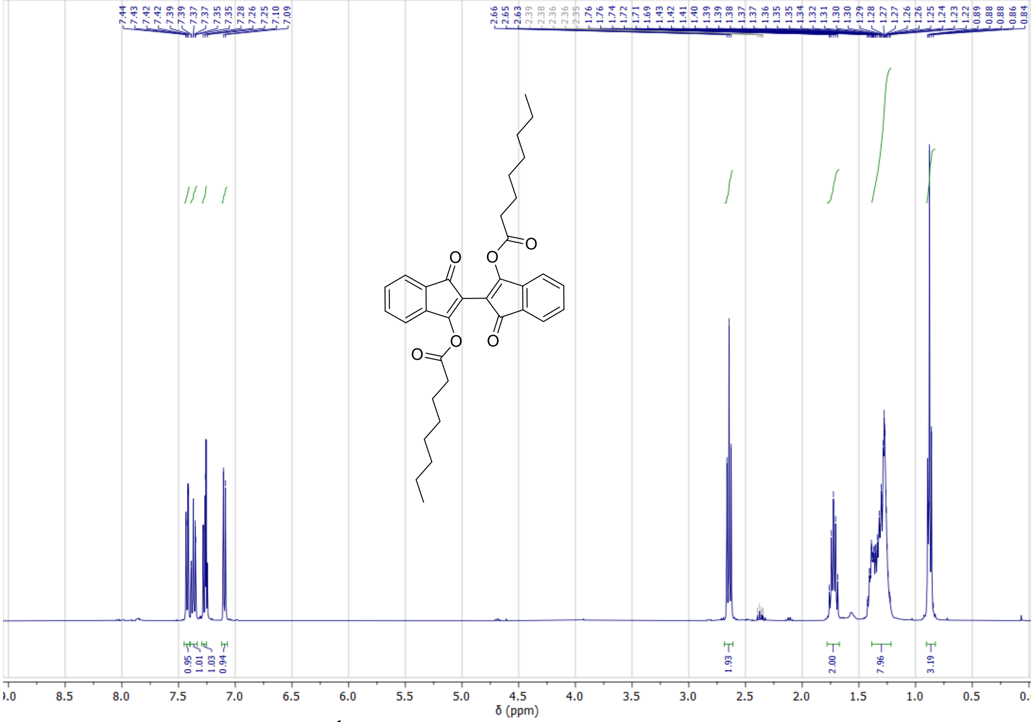

^{1}H NMR spectrum of **BIT-8** in $CDCl_3$

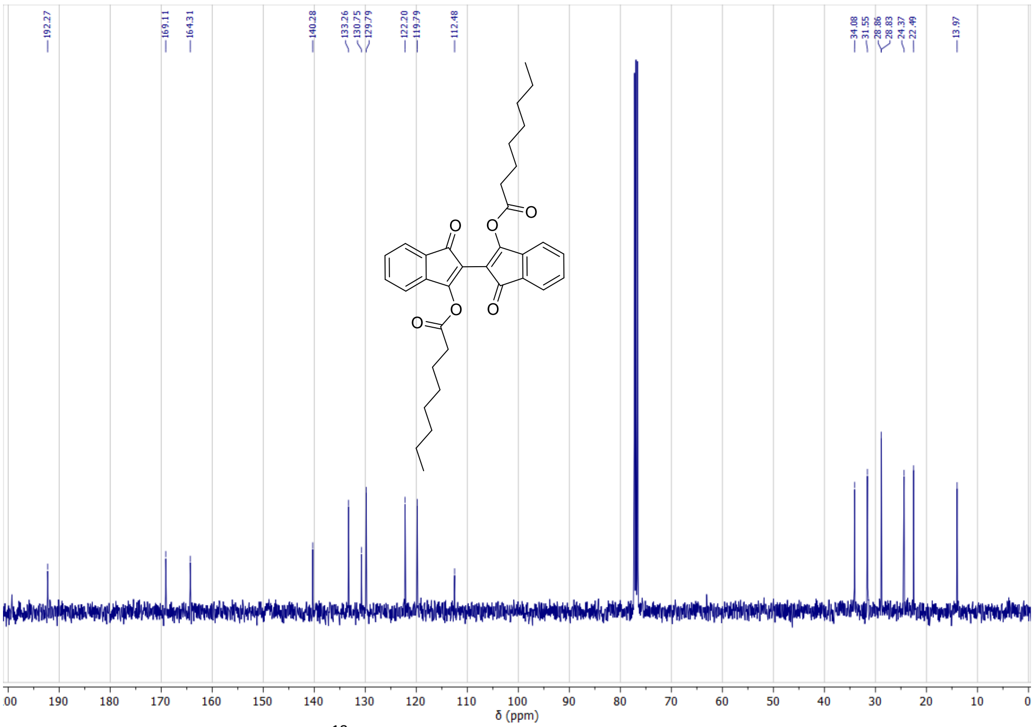

^{13}C NMR spectrum of **BIT-8** in $CDCl_3$

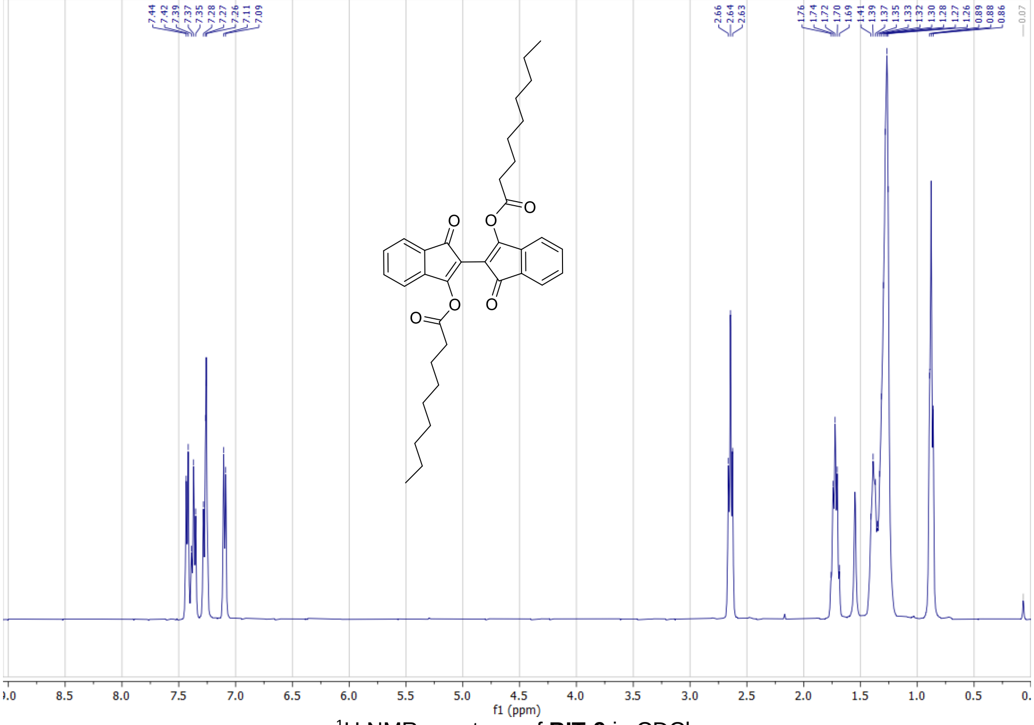

^{1}H NMR spectrum of **BIT-9** in $CDCl_3$

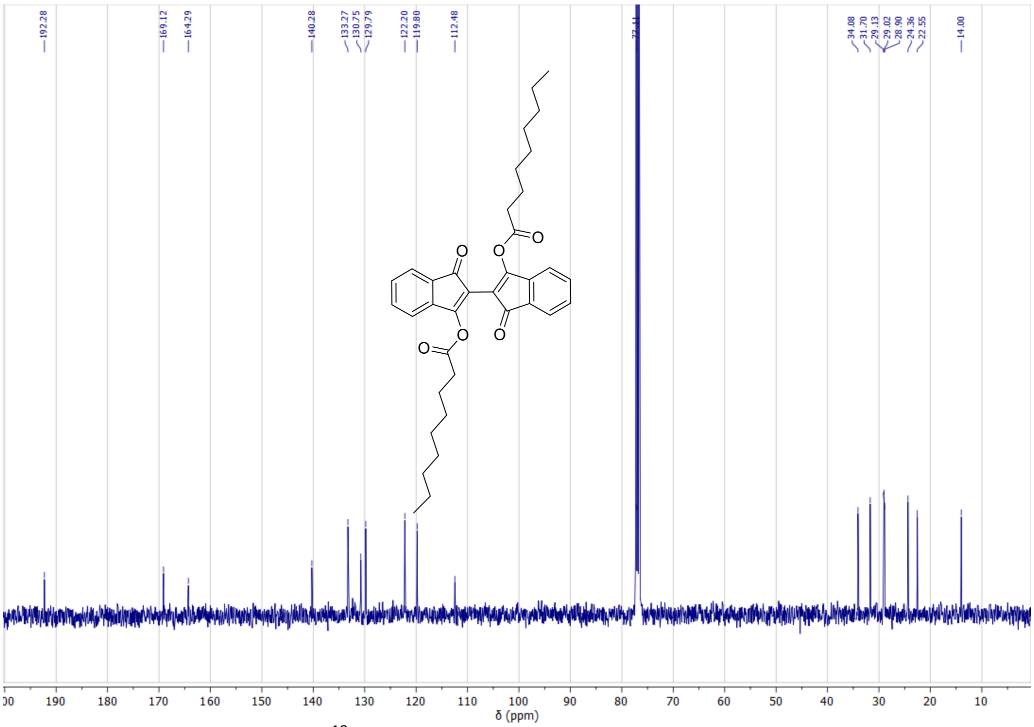

^{13}C NMR spectrum of **BIT-9** in $CDCl_3$

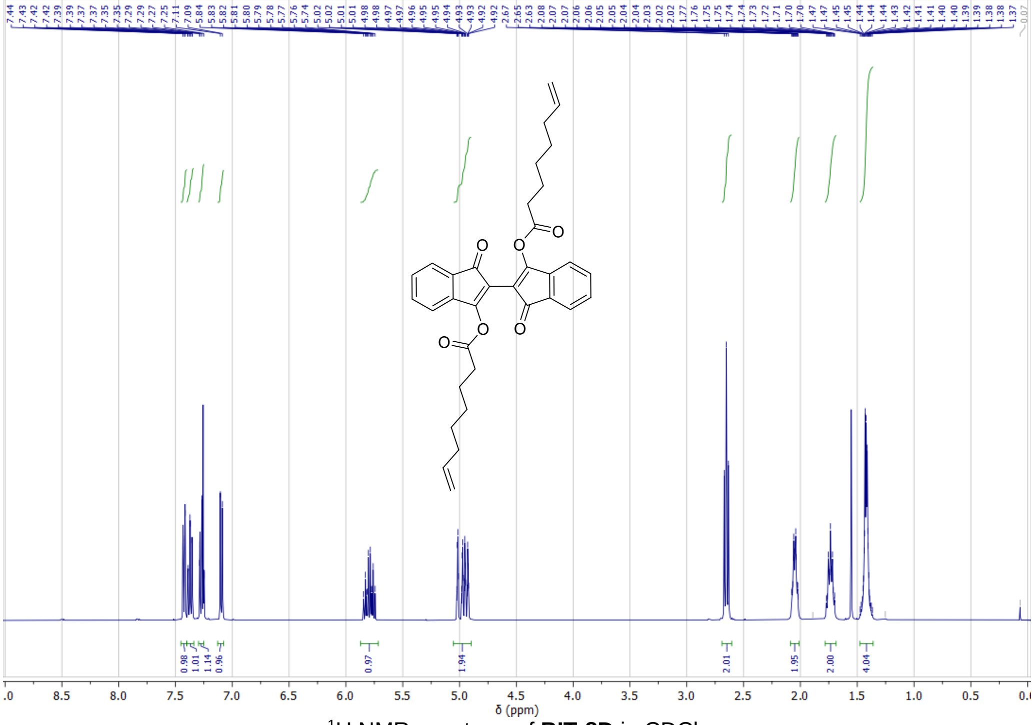

^{1}H NMR spectrum of **BIT-8D** in $CDCl_3$

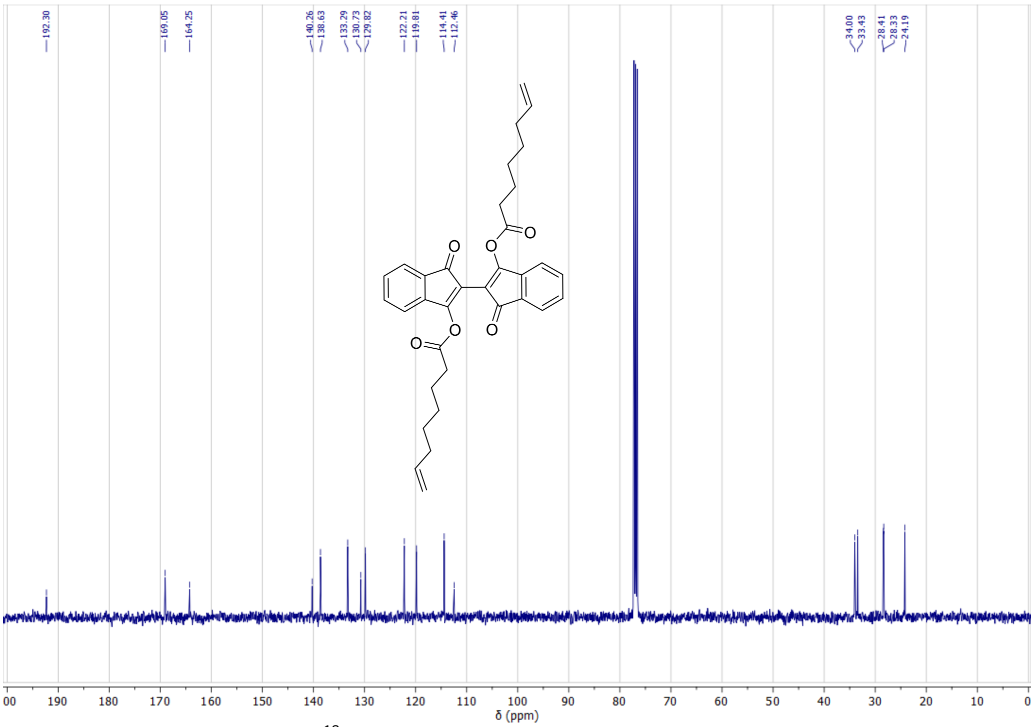

^{13}C NMR spectrum of **BIT-8D** in $CDCl_3$

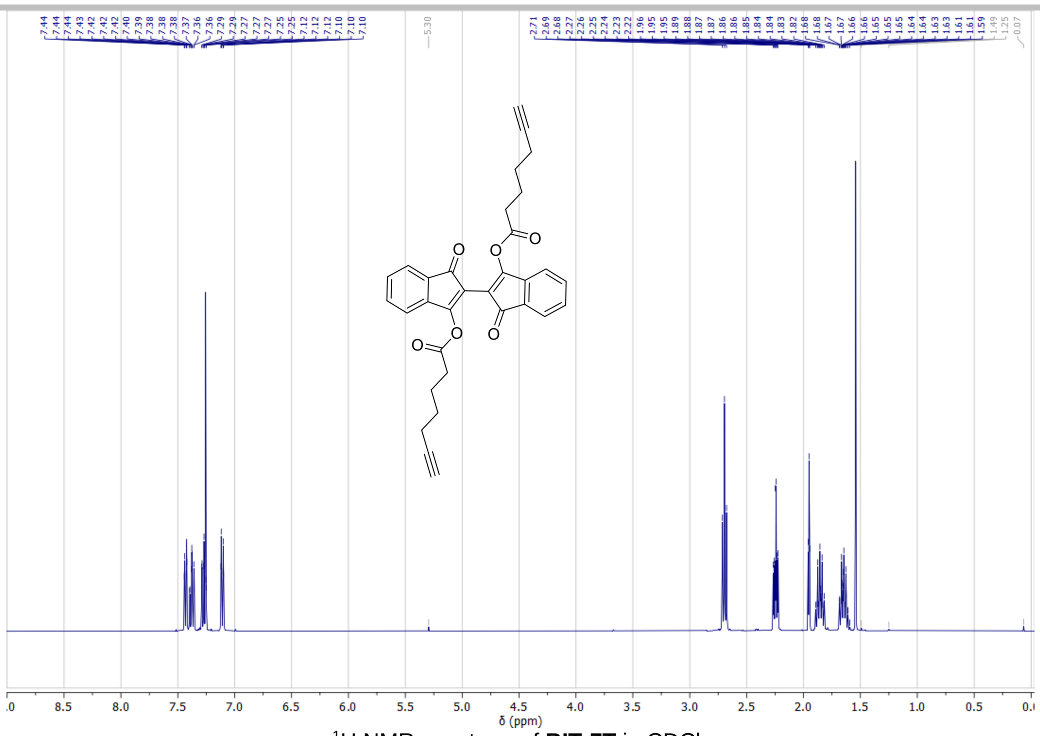

^{1}H NMR spectrum of **BIT-7T** in $CDCl_3$

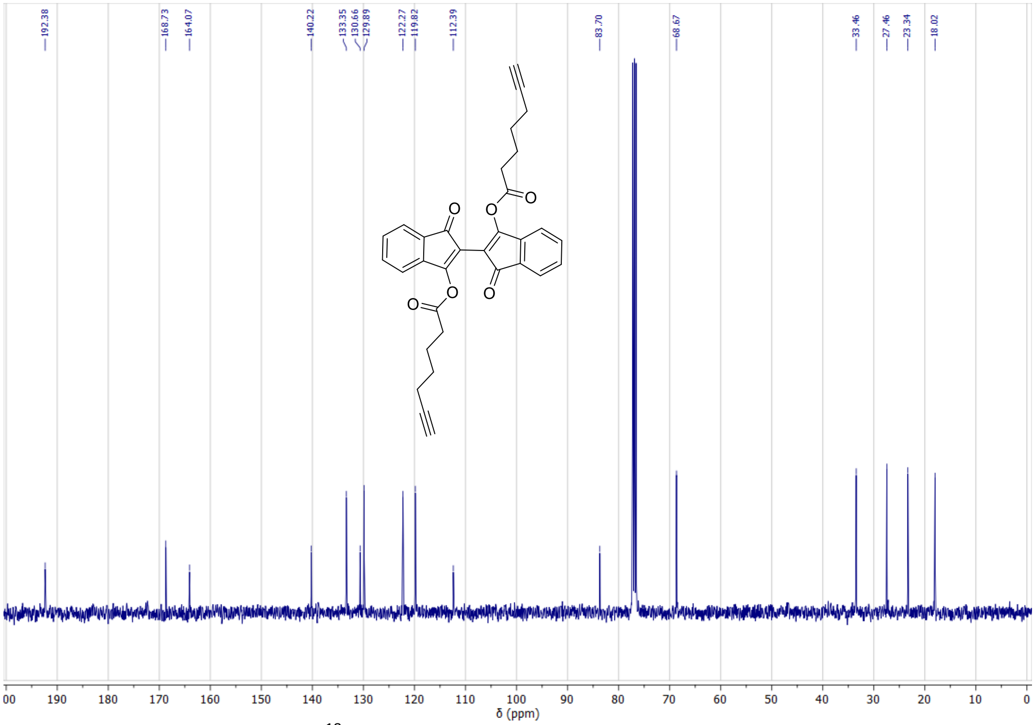

^{13}C NMR spectrum of **BIT-7T** in $CDCl_3$

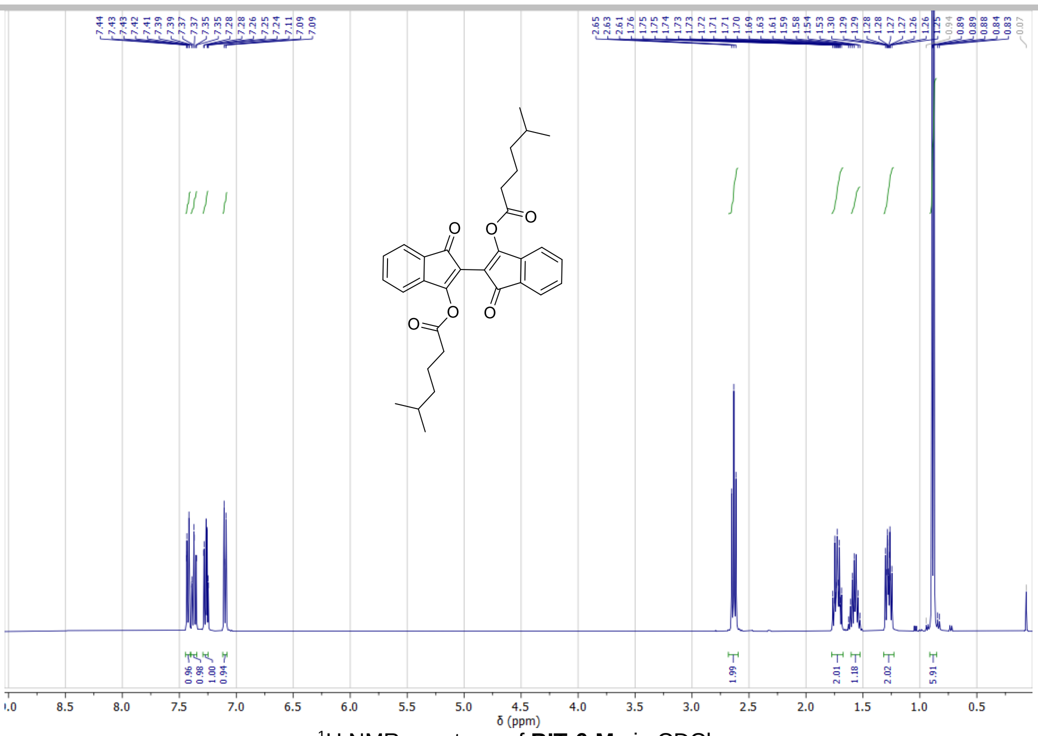

^{1}H NMR spectrum of **BIT-6-Me** in $CDCl_3$

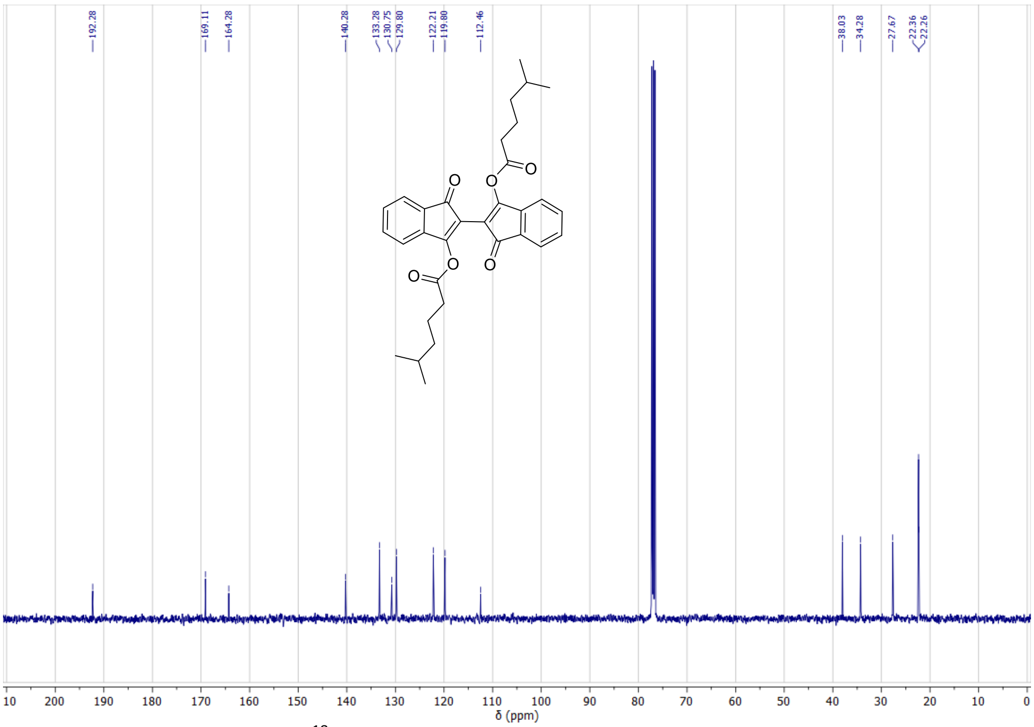

^{13}C NMR spectrum of **BIT-6-Me** in $CDCl_3$

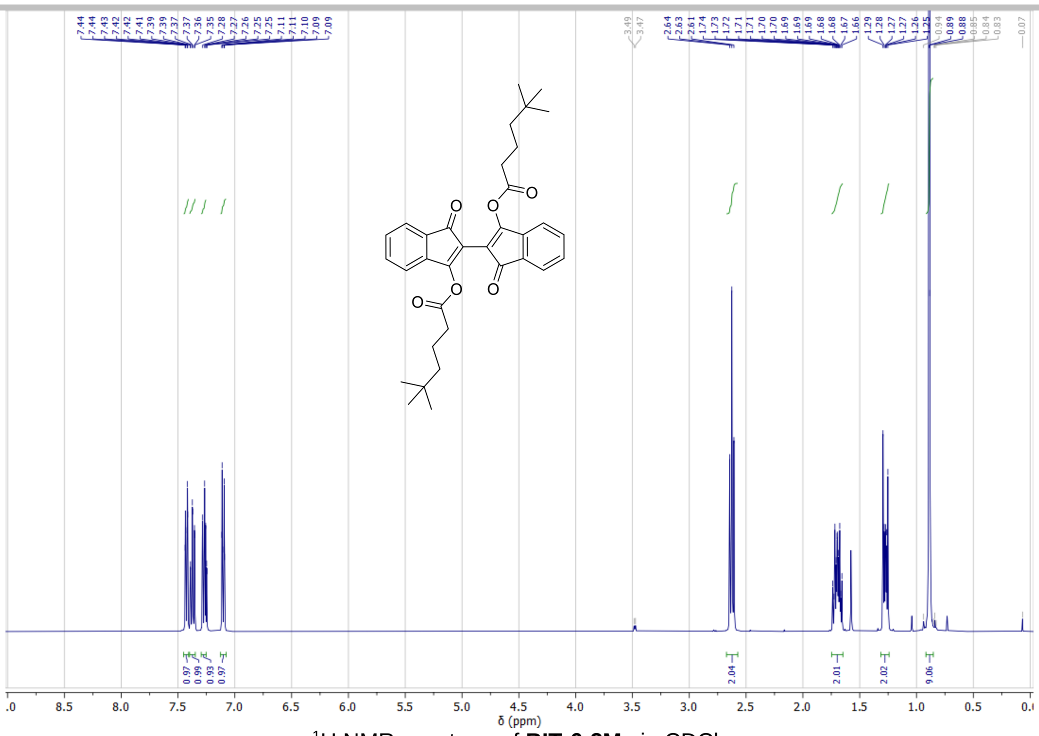

^1H NMR spectrum of **BIT-6-2Me** in $CDCl_3$

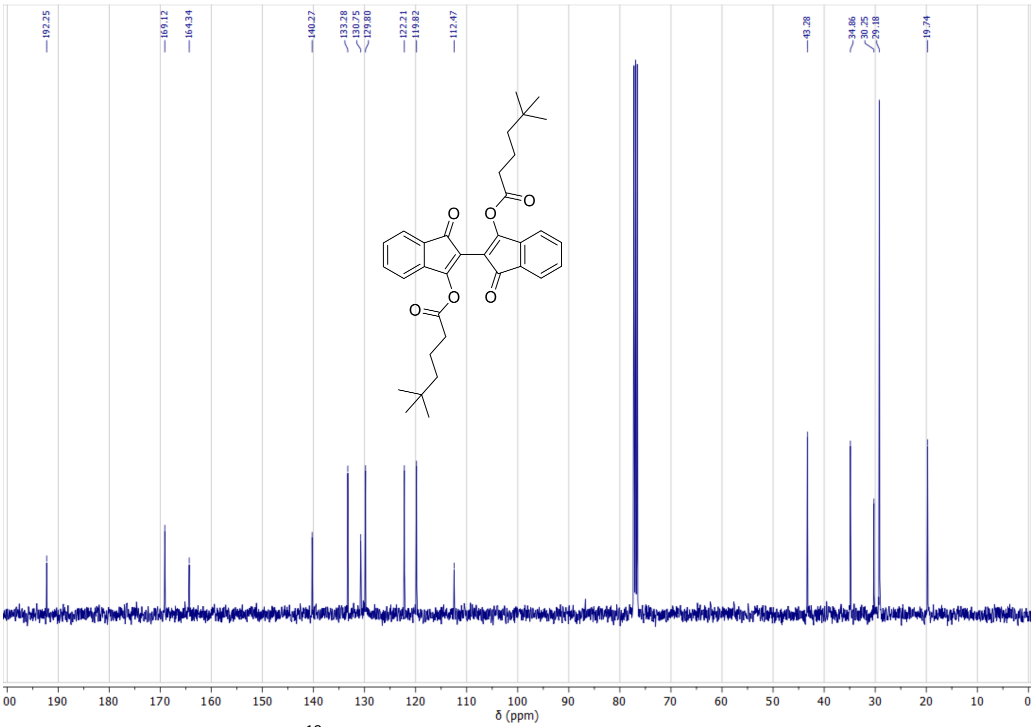

^{13}C NMR spectrum of **BIT-6-2Me** in $CDCl_3$

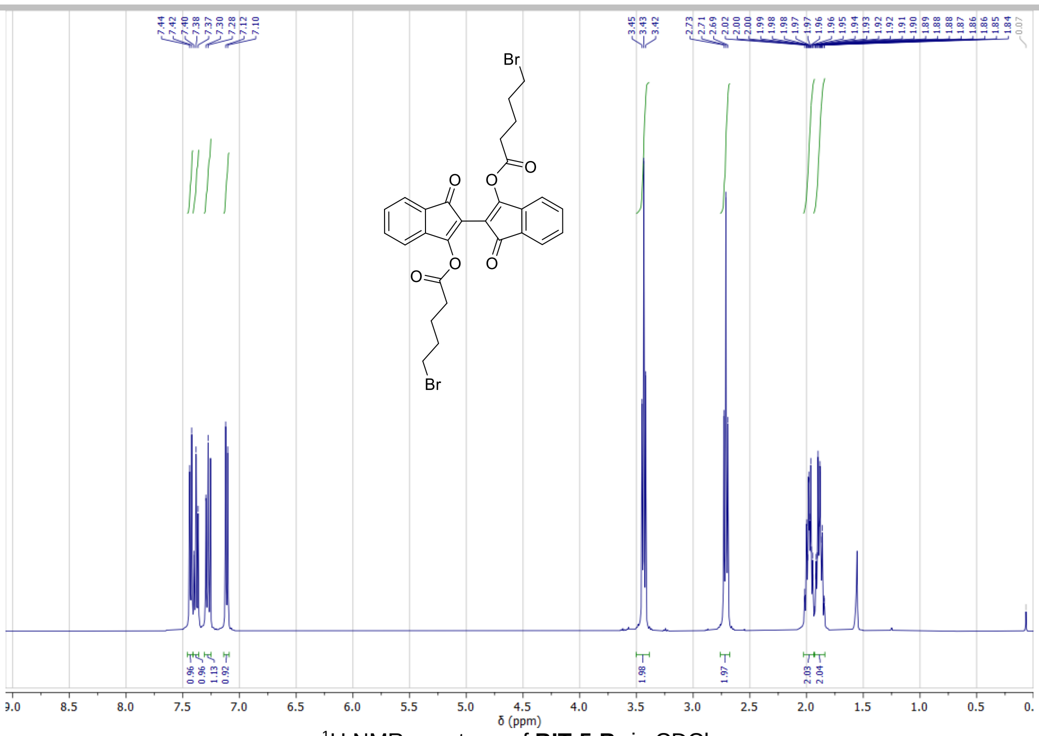

^1H NMR spectrum of **BIT-5-Br** in $CDCl_3$

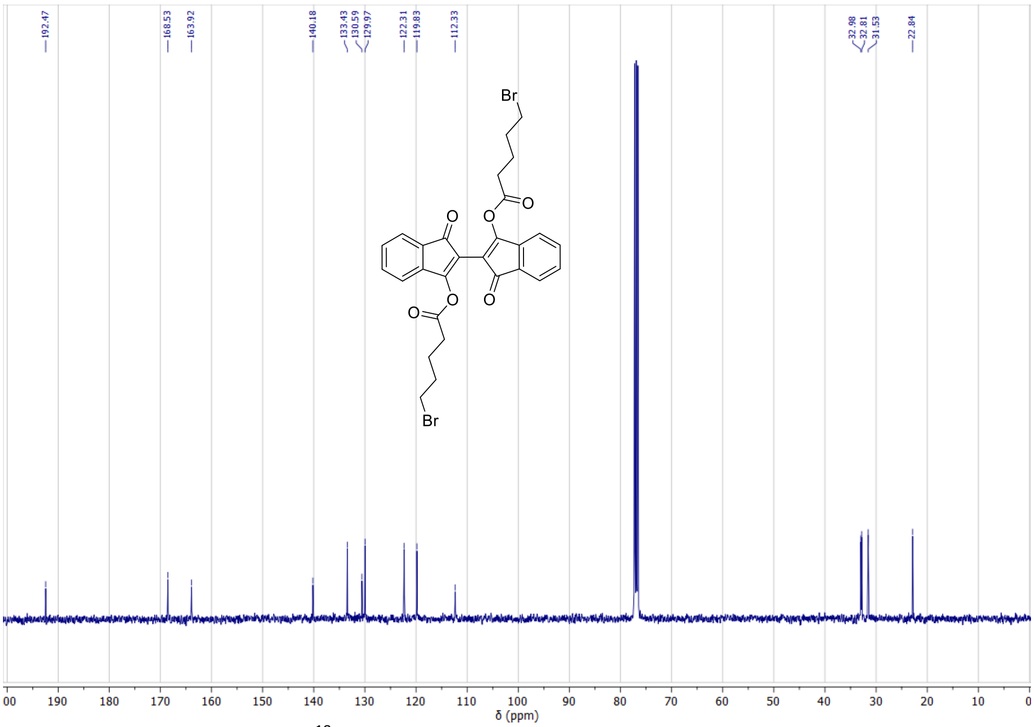

^{13}C NMR spectrum of **BIT-5-Br** in $CDCl_3$

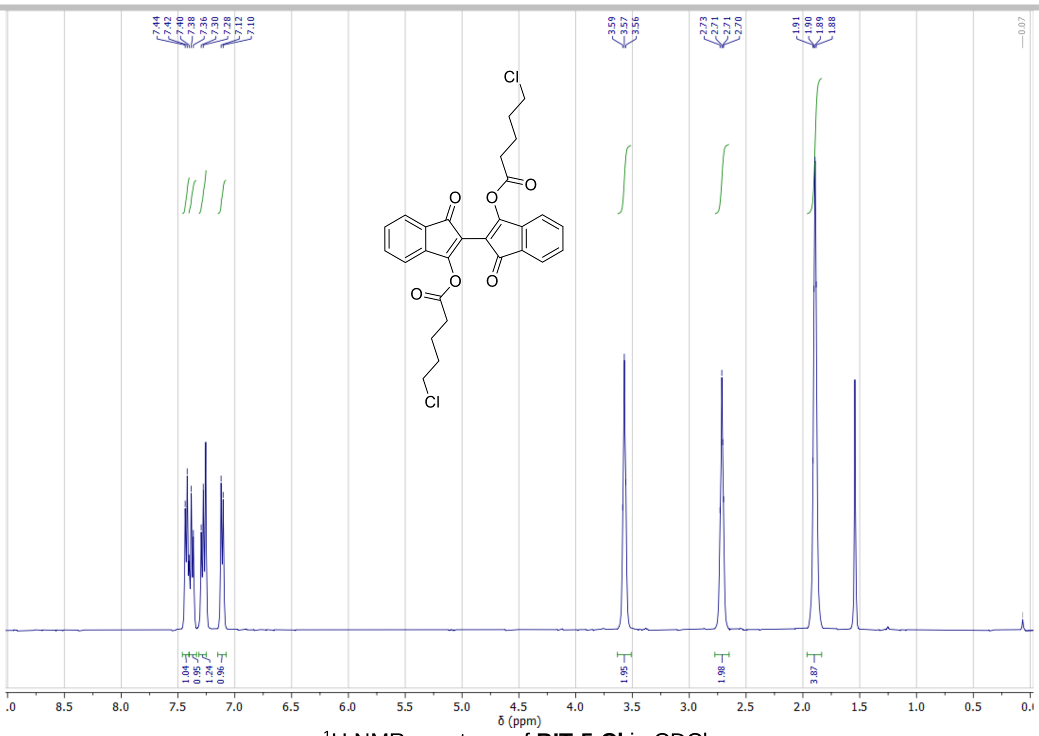

^{1}H NMR spectrum of **BIT-5-Cl** in $CDCl_3$

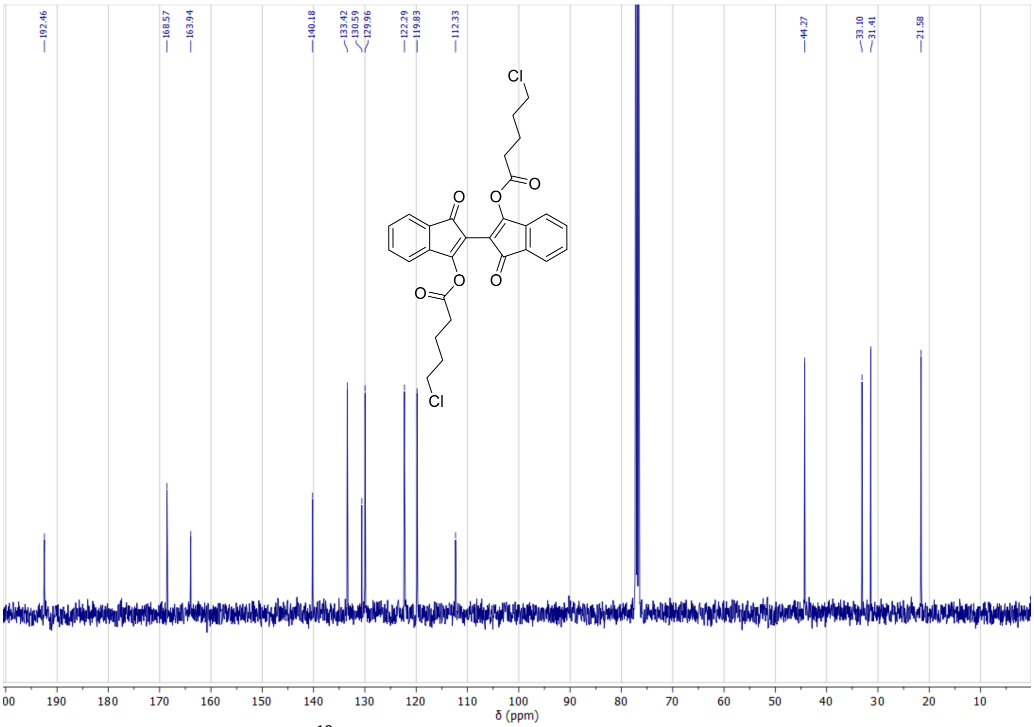

^{13}C NMR spectrum of **BIT-5-Cl** in $CDCl_3$

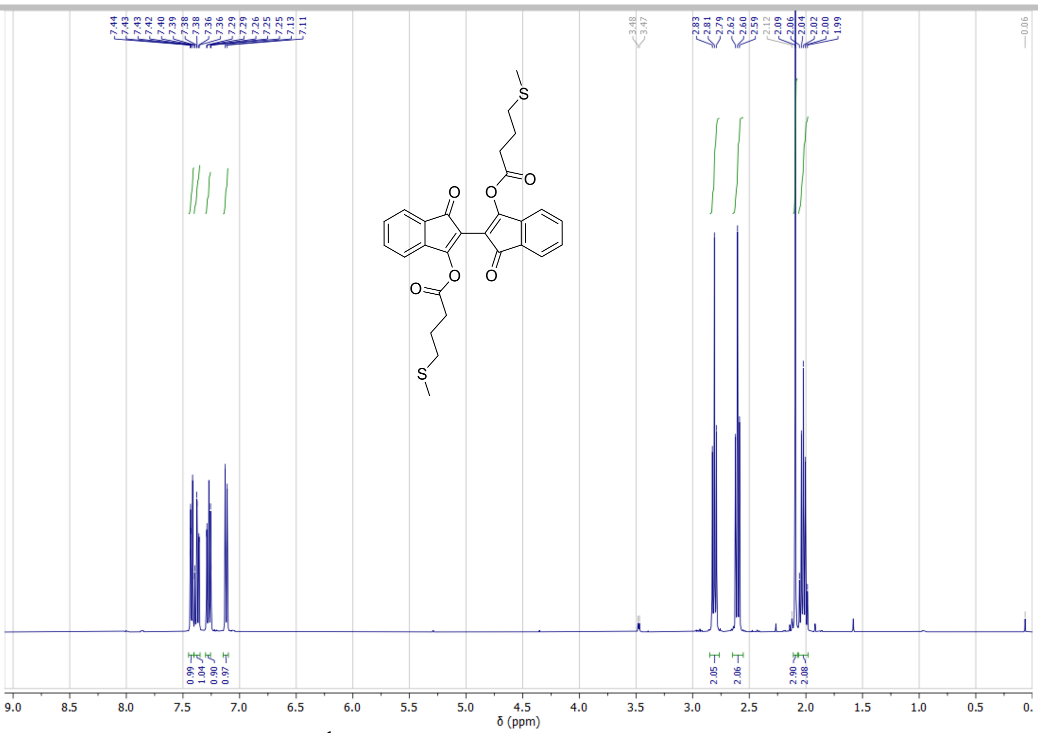

^1H NMR spectrum of **BIT-6-S** in $CDCl_3$

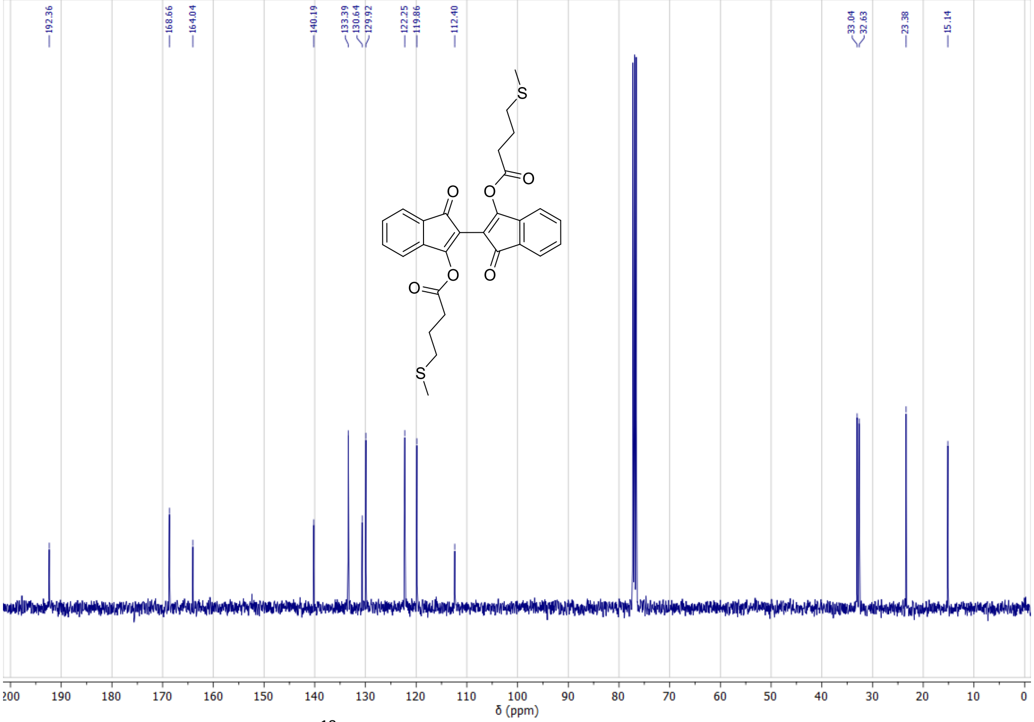

^{13}C NMR spectrum of **BIT-6-S** in $CDCl_3$

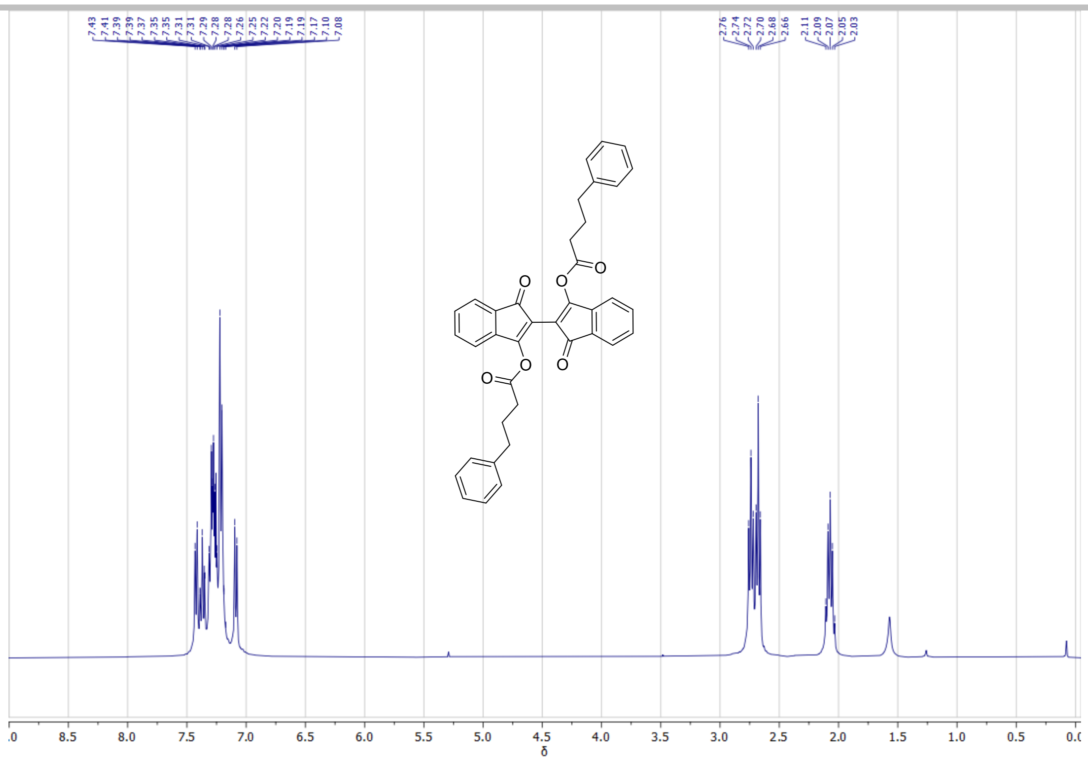

^{1}H NMR spectrum of **BIT-4-Ph** in $CDCl_3$

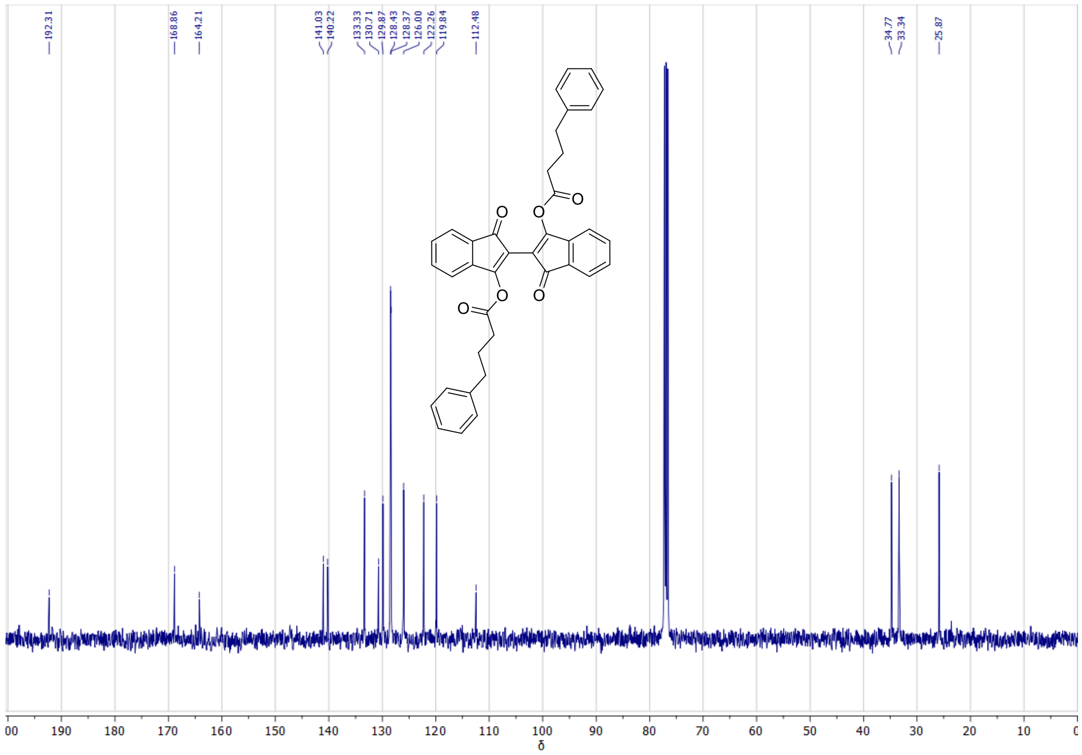

^{13}C NMR spectrum of **BIT-4-Ph** in $CDCl_3$

1.9. Cambridge Crystallographic Data Center Reference Numbers for Crystals

Complete crystallographic data, in CIF format, have been deposited with the Cambridge Crystallographic Data Centre and can be obtained free of charge from The Cambridge Crystallographic Data Centre via www.ccdc.cam.ac.uk/data_request/cif.

Crystal Name	CCDC Number	Crystal Name	CCDC Number
BIT-6	2177475	PBIT-6	2088147
BIT-7	2177481	PBIT-7	2133259
BIT-8	2177476	PBIT-8	2088148
BIT-9	2177478	PBIT-9	2133258
BIT-8D	2177479	PBIT-8D	2131647
BIT-6-Me	2177477	PBIT-6-Me	2131645
BIT-5-Br	2177480	PBIT-5-Br	2177487
BIT-5-Cl	2177483	BIT-6-2Me	2177484
BIT-6-O	2177486	BIT-6-S	2177482
BIT-7T	2177485	BIT-5	2206363
BIT-4-Ph	2206364		

2. Results and Discussions

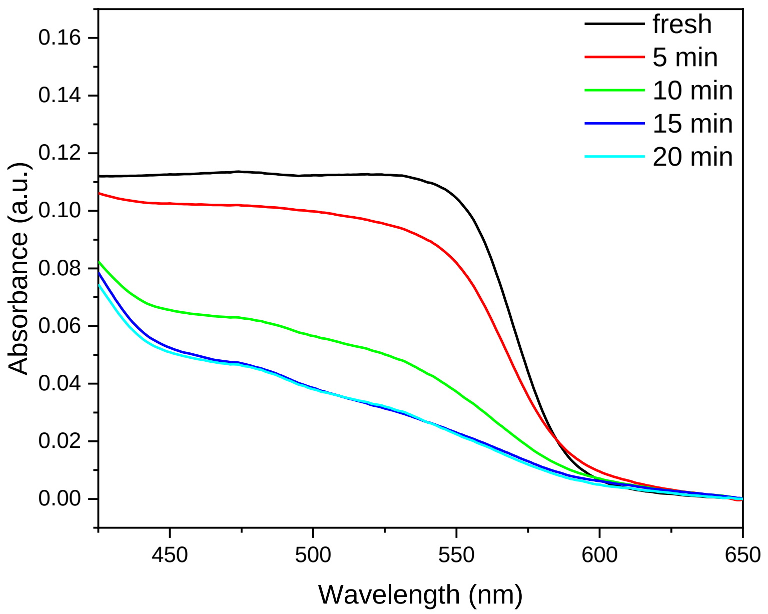

Figure S1. Change in UV-vis spectrum during the crystalline-state polymerization of **BIT-6** under visible light irradiation at room temperature.

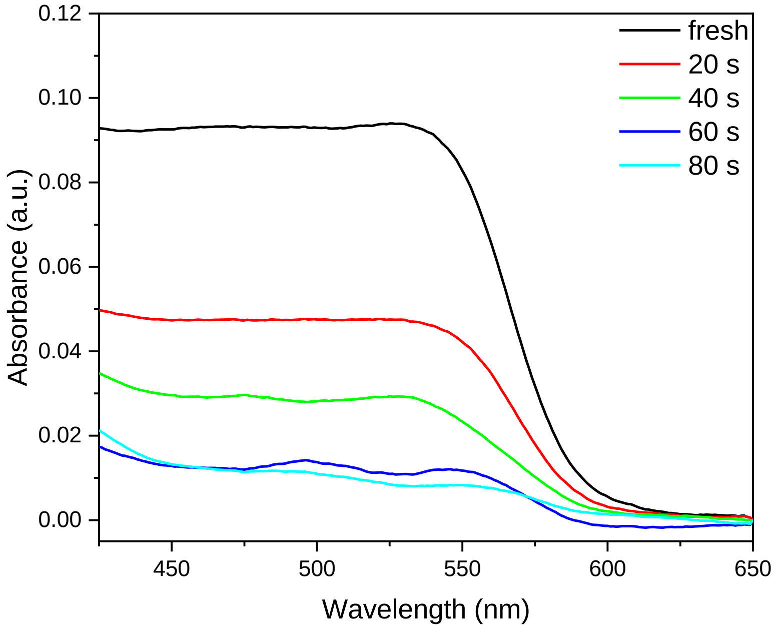

Figure S2. Change in UV-vis spectrum during the crystalline-state polymerization of **BIT-7** under visible light irradiation at room temperature.

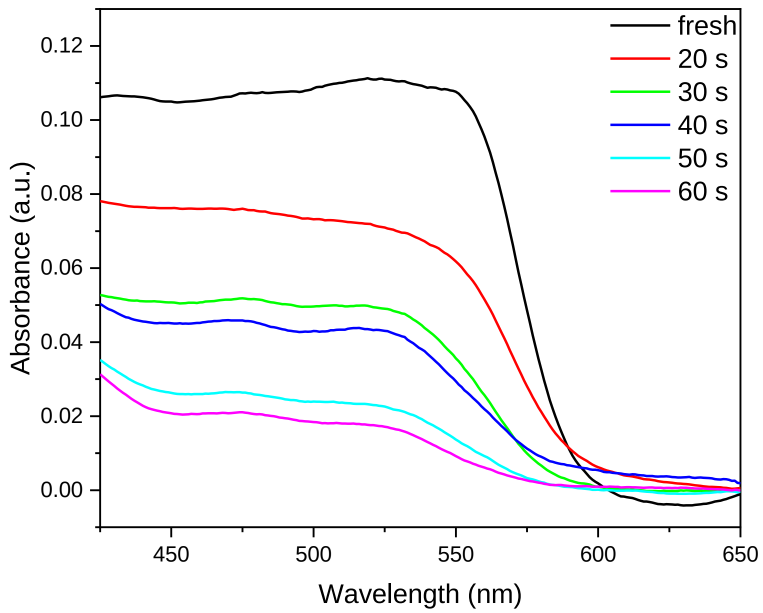

Figure S3. Change in UV-vis spectrum during the crystalline-state polymerization of **BIT-8** under visible light irradiation at room temperature.

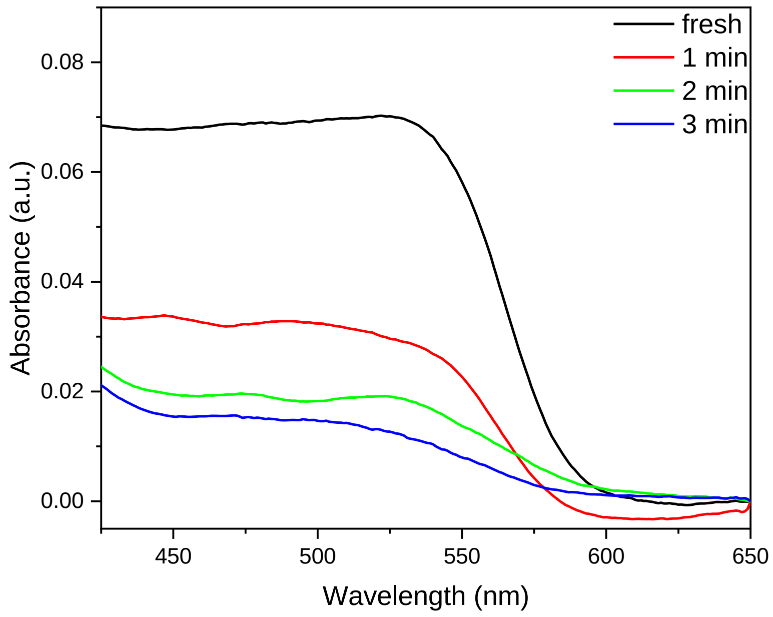

Figure S4. Change in UV-vis spectrum during the crystalline-state polymerization of **BIT-6-Me** under visible light irradiation at room temperature.

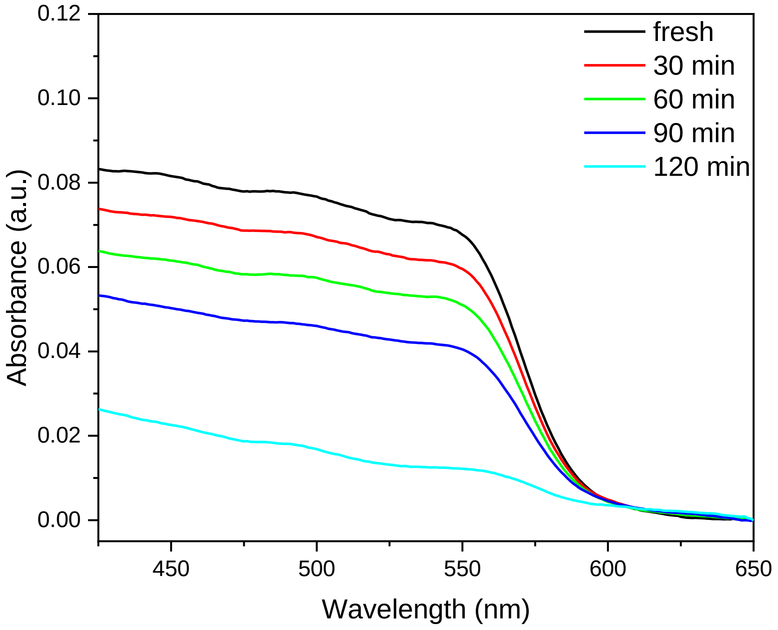

Figure S5. Change in UV-vis spectrum during the crystalline-state polymerization of **BIT-8D** under visible light irradiation at room temperature.

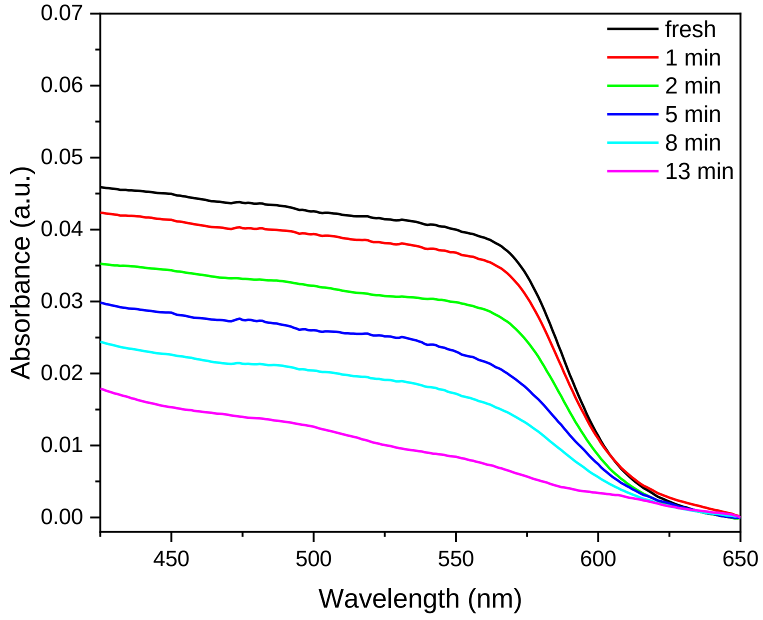

Figure S6. Change in UV-vis spectrum during the crystalline-state polymerization of **BIT-5-Br** under visible light irradiation at room temperature.

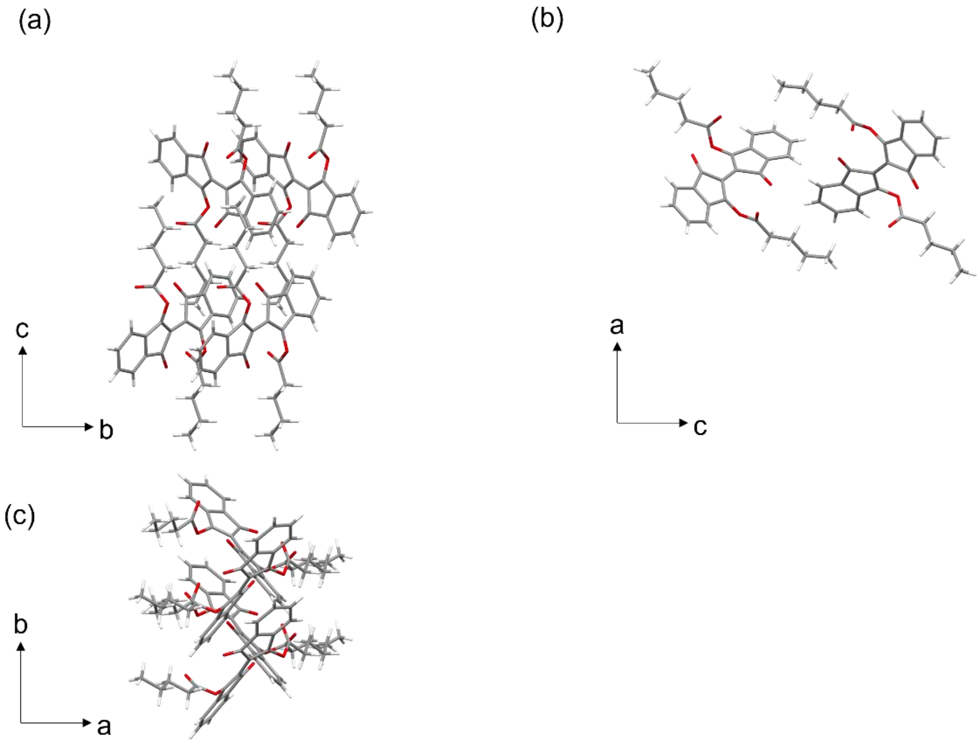

Figure S7. Single crystal X-ray diffraction results for photostable polymorph of **BIT-5**. (a) View along a axis. (b) View along b axis. (c) View along c axis.

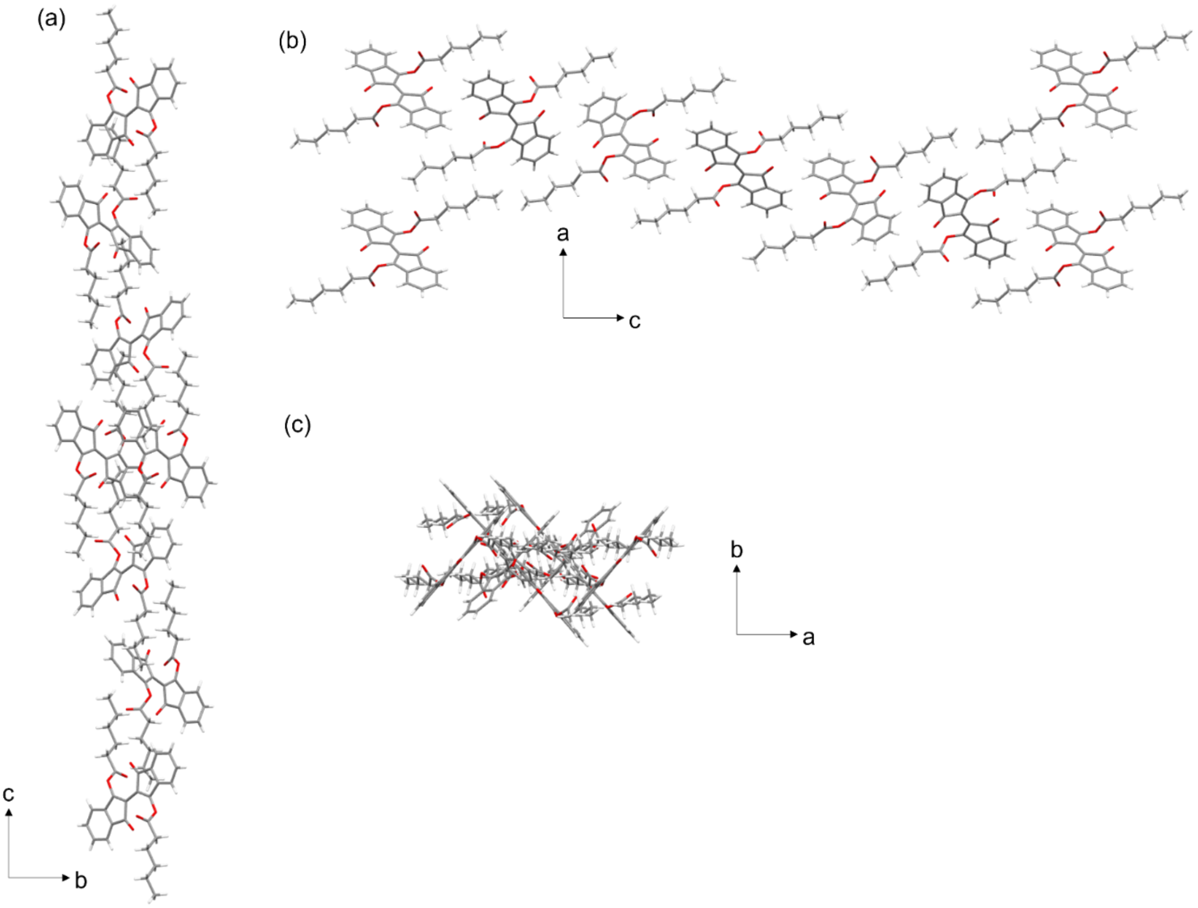

Figure S8. Single crystal X-ray diffraction results for **BIT-6**. (a) View along a axis. (b) View along b axis. (c) View along c axis.

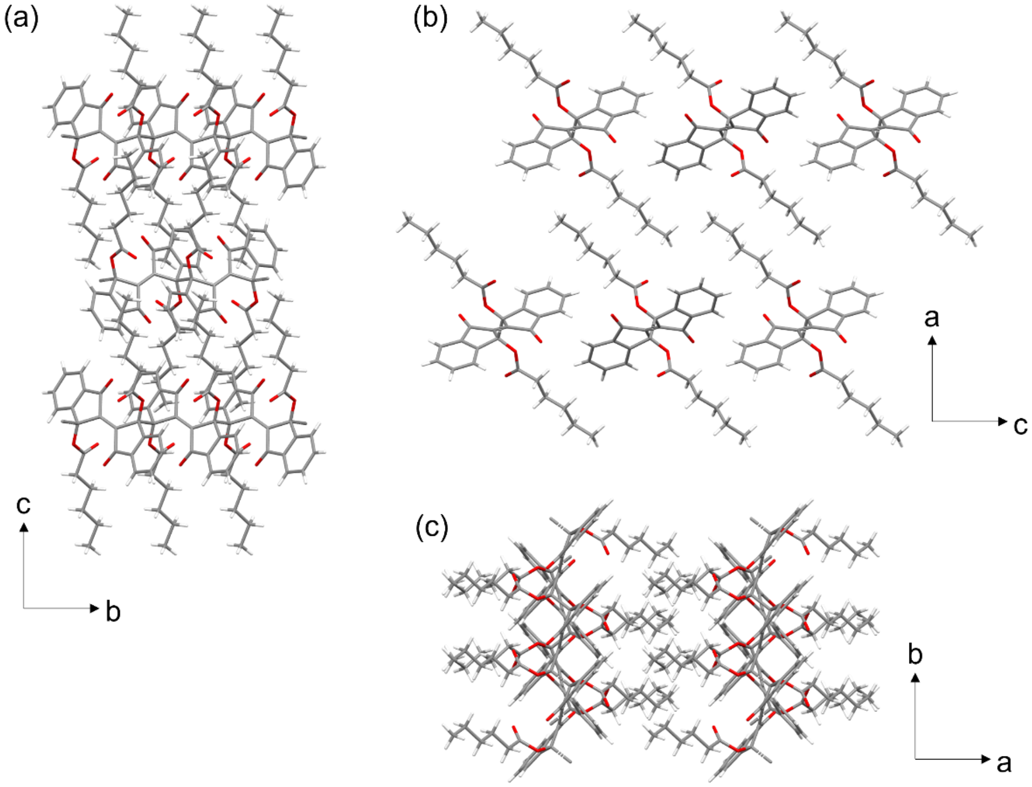

Figure S9. Single crystal X-ray diffraction results for **PBIT-6**. (a) View along a axis. (b) View along b axis. (c) View along c axis.

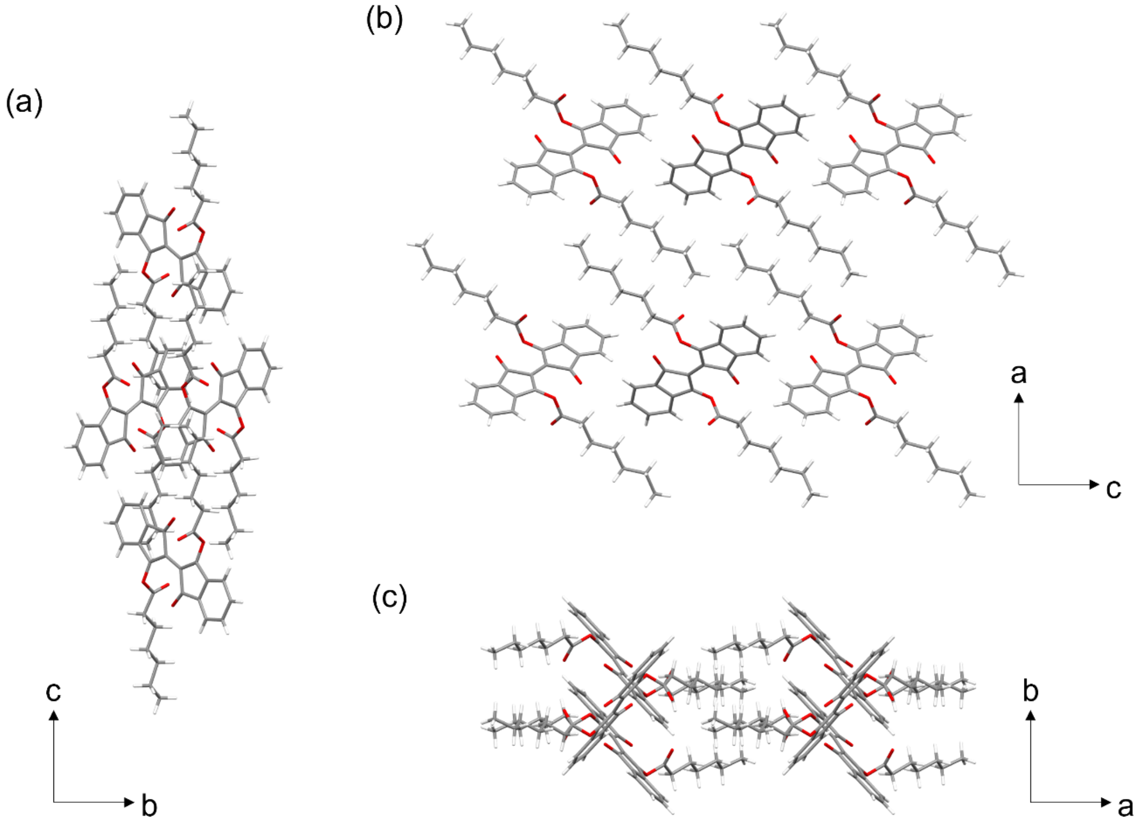

Figure S10. Single crystal X-ray diffraction results for **BIT-7**. (a) View along a axis. (b) View along b axis. (c) View along c axis.

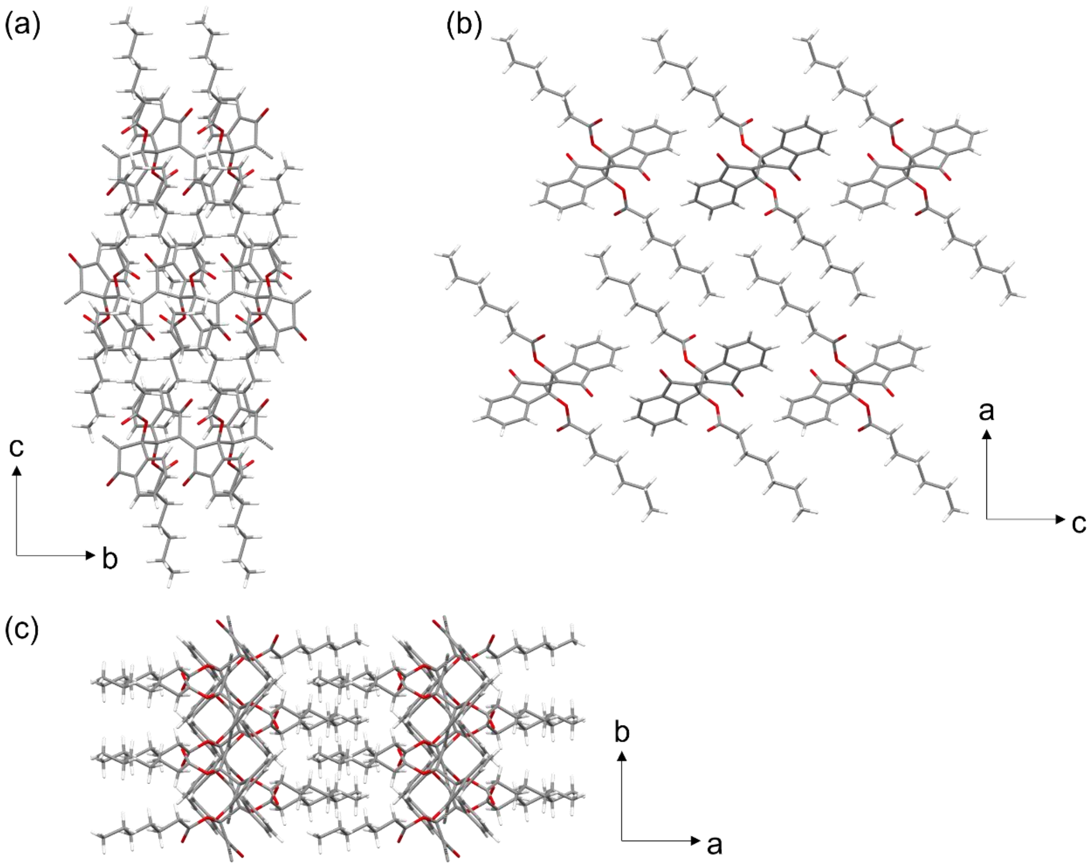

Figure S11. Single crystal X-ray diffraction results for **PBIT-7**. (a) View along a axis. (b) View along b axis. (c) View along c axis.

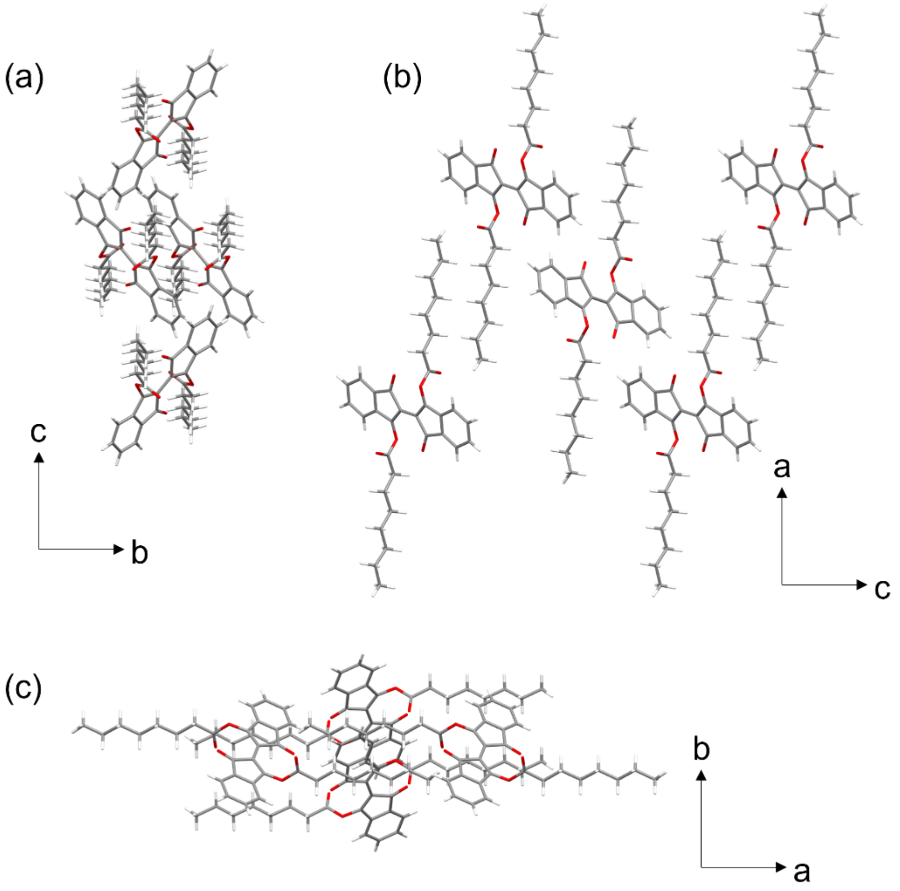

Figure S12. Single crystal X-ray diffraction results for **BIT-8**. (a) View along a axis. (b) View along b axis. (c) View along c axis.

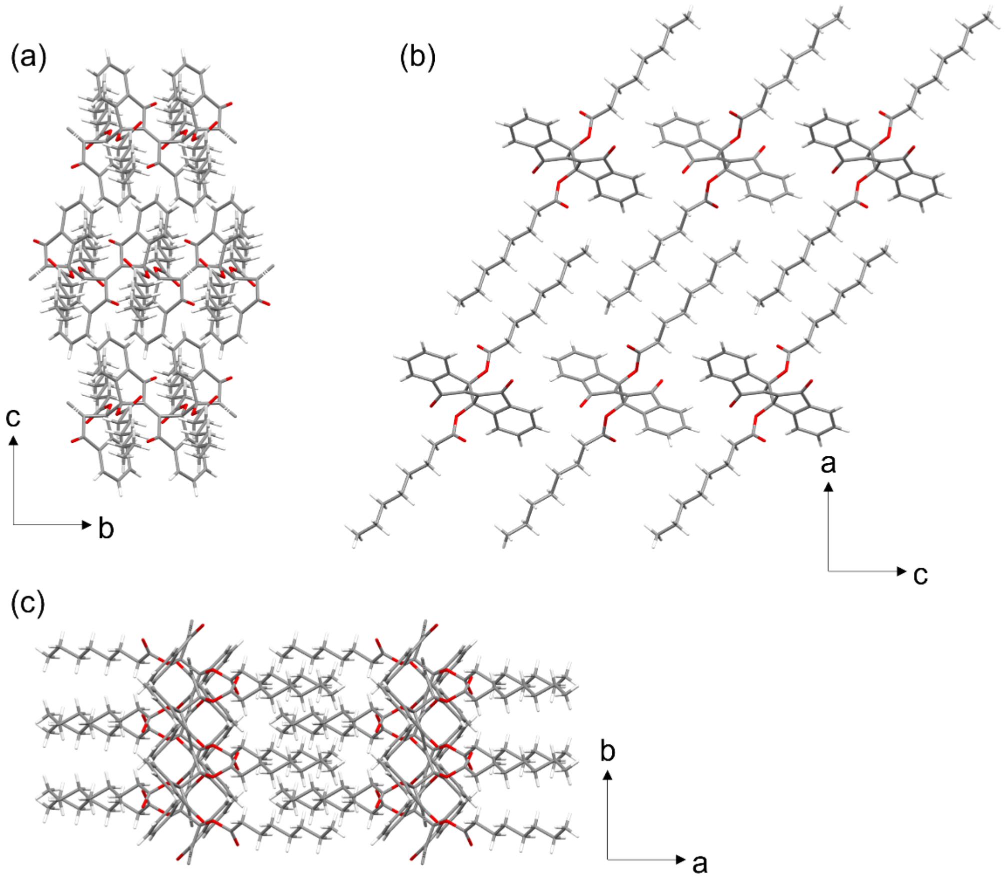

Figure S13. Single crystal X-ray diffraction results for **PBIT-8**. (a) View along a axis. (b) View along b axis. (c) View along c axis.

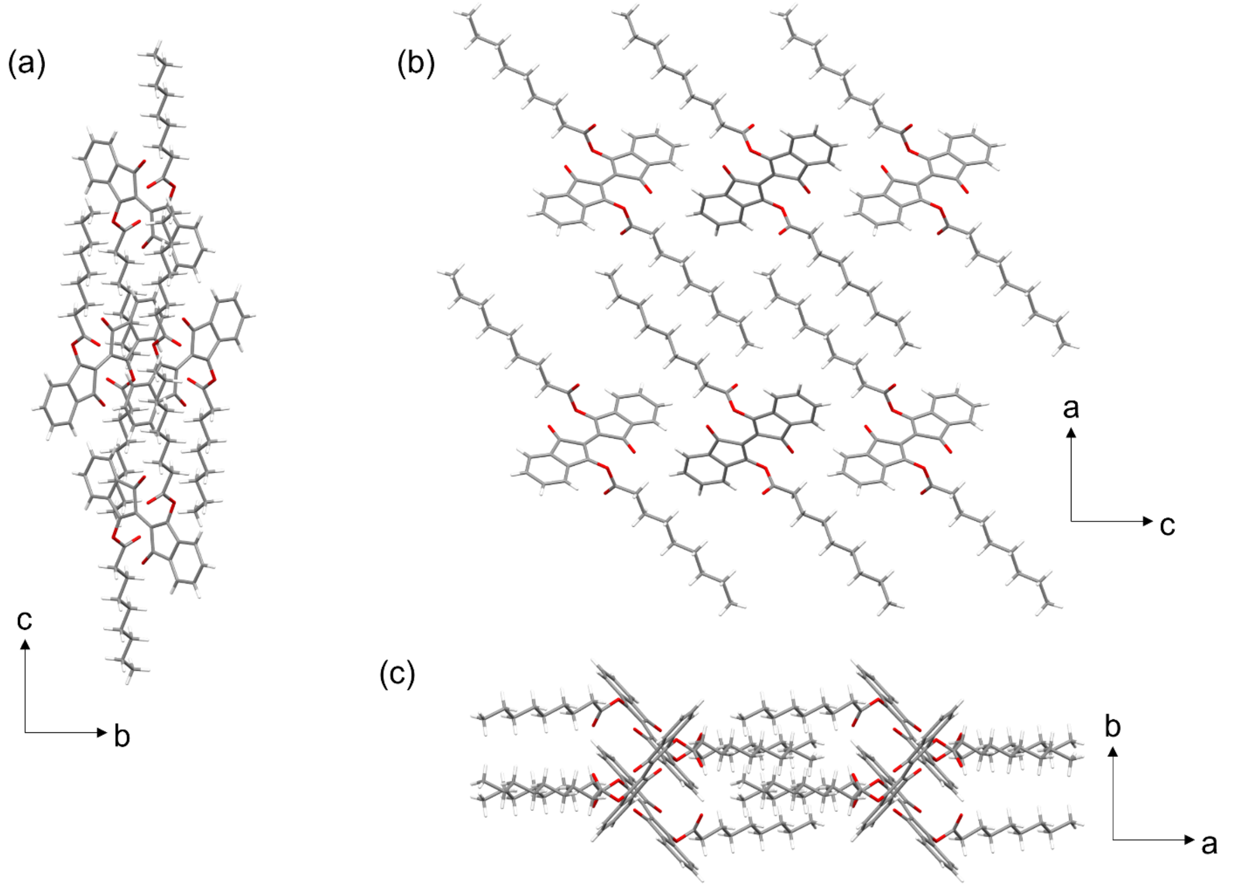

Figure S14. Single crystal X-ray diffraction results for **BIT-9**. (a) View along a axis. (b) View along b axis. (c) View along c axis.

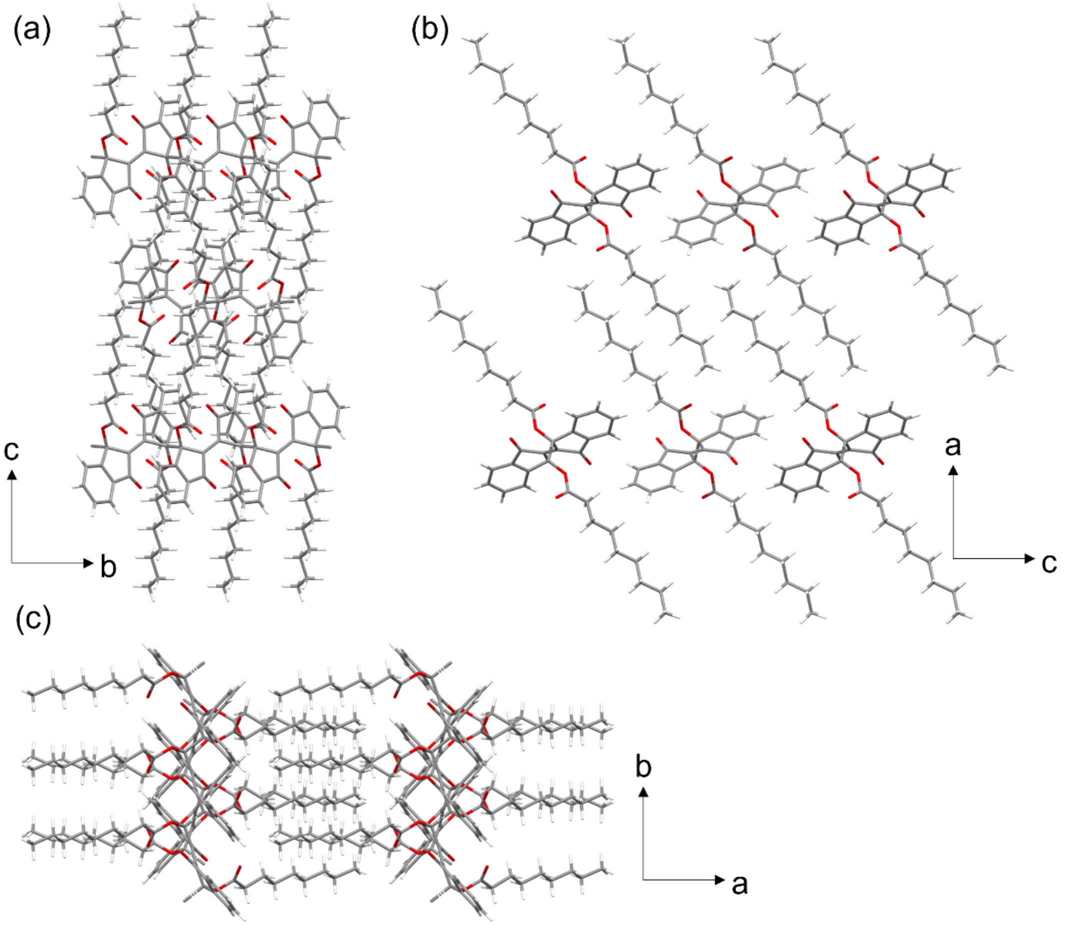

Figure S15. Single crystal X-ray diffraction results for **PBIT-9**. (a) View along a axis. (b) View along b axis. (c) View along c axis.

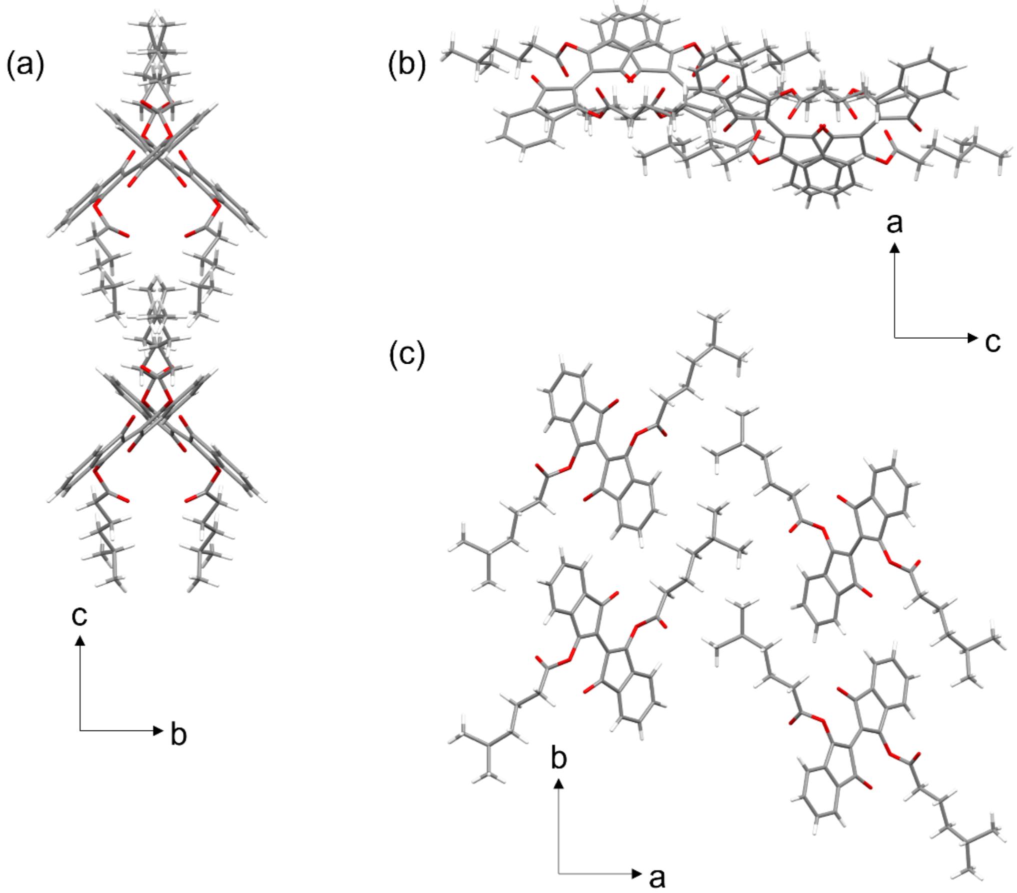

Figure S16. Single crystal X-ray diffraction results for **BIT-6-Me**. (a) View along a axis. (b) View along b axis. (c) View along c axis.

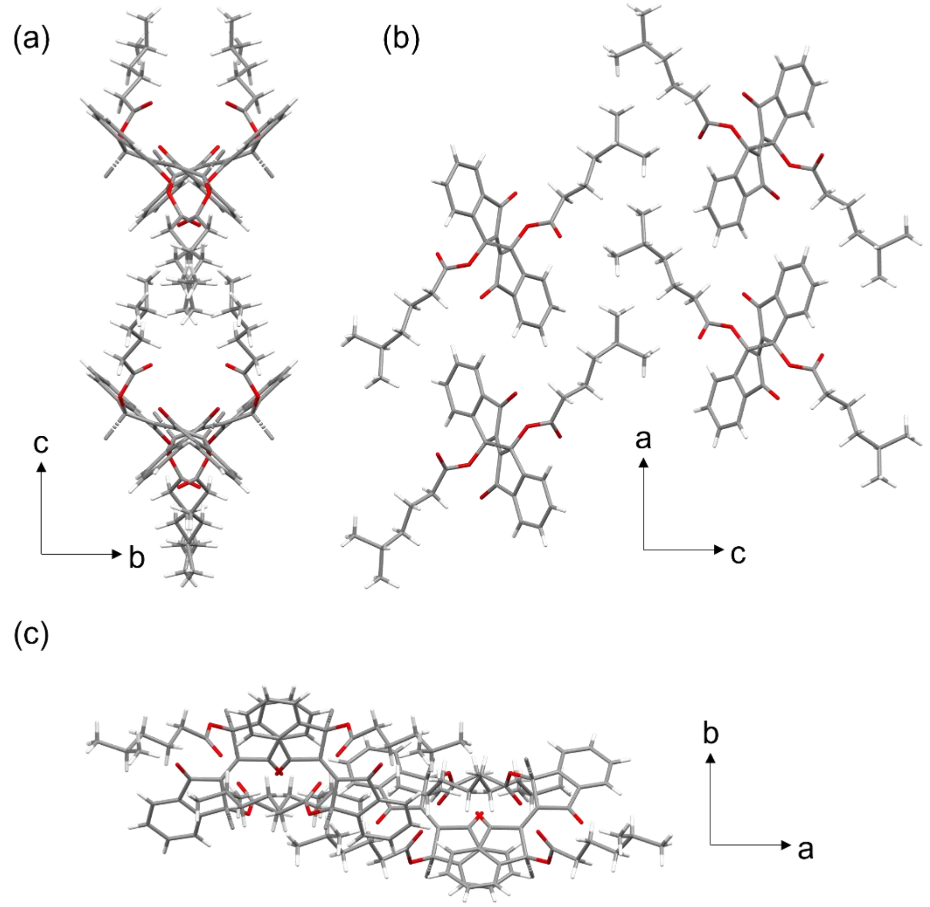

Figure S17. Single crystal X-ray diffraction results for **PBIT-6-Me**. (a) View along a axis. (b) View along b axis. (c) View along c axis

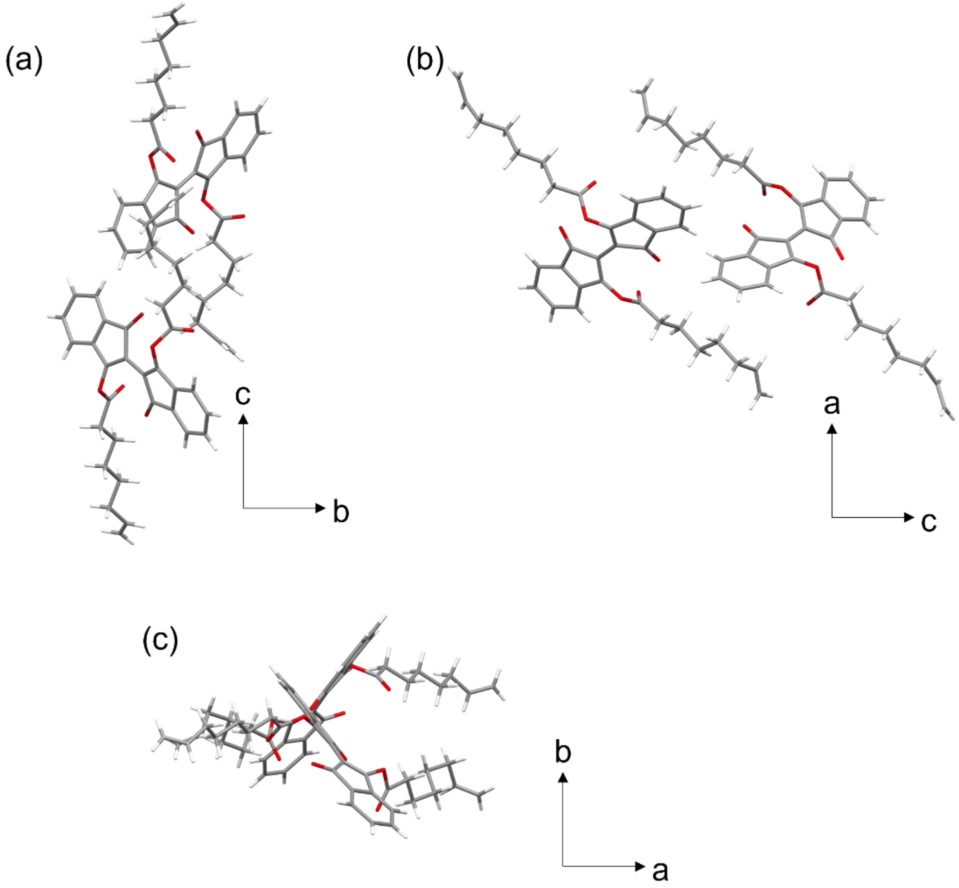

Figure 18. Single crystal X-ray diffraction results for **BIT-8D**. (a) View along a axis. (b) View along b axis. (c) View along c axis.

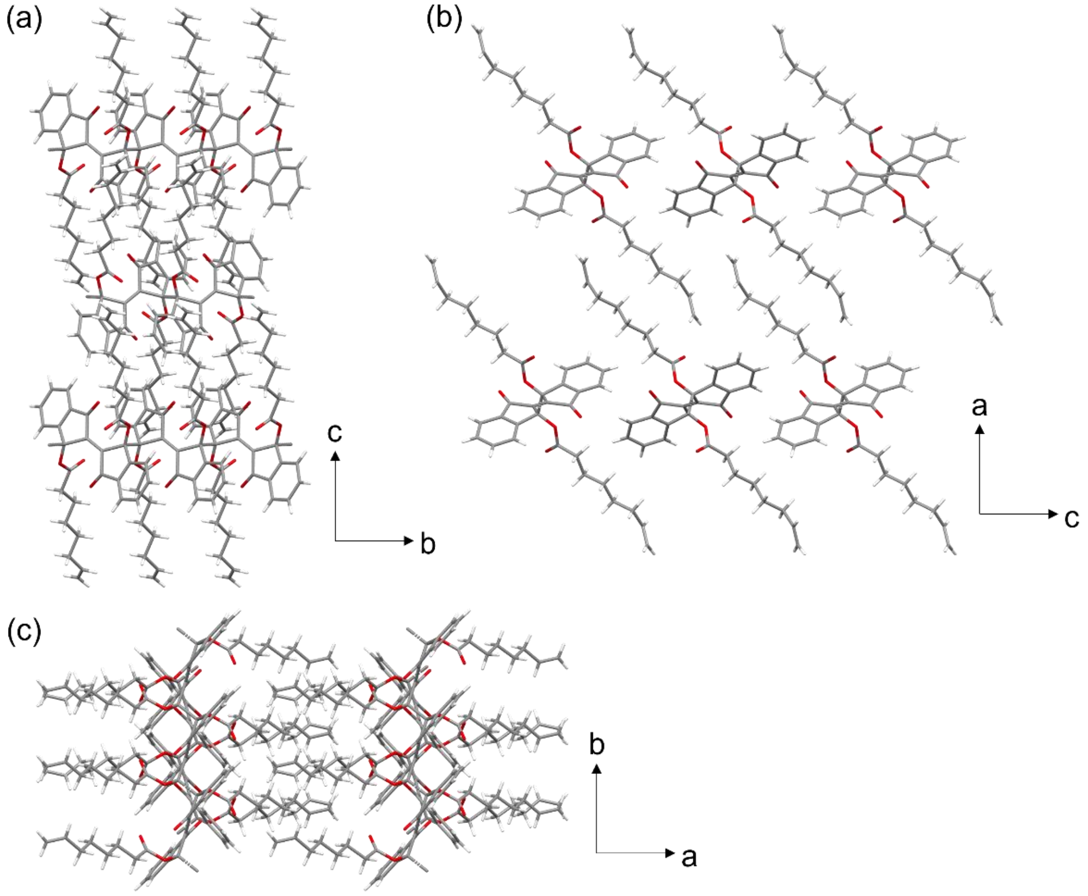

Figure S19. Single crystal X-ray diffraction results for **PBIT-8D**. (a) View along a axis. (b) View along b axis. (c) View along c axis.

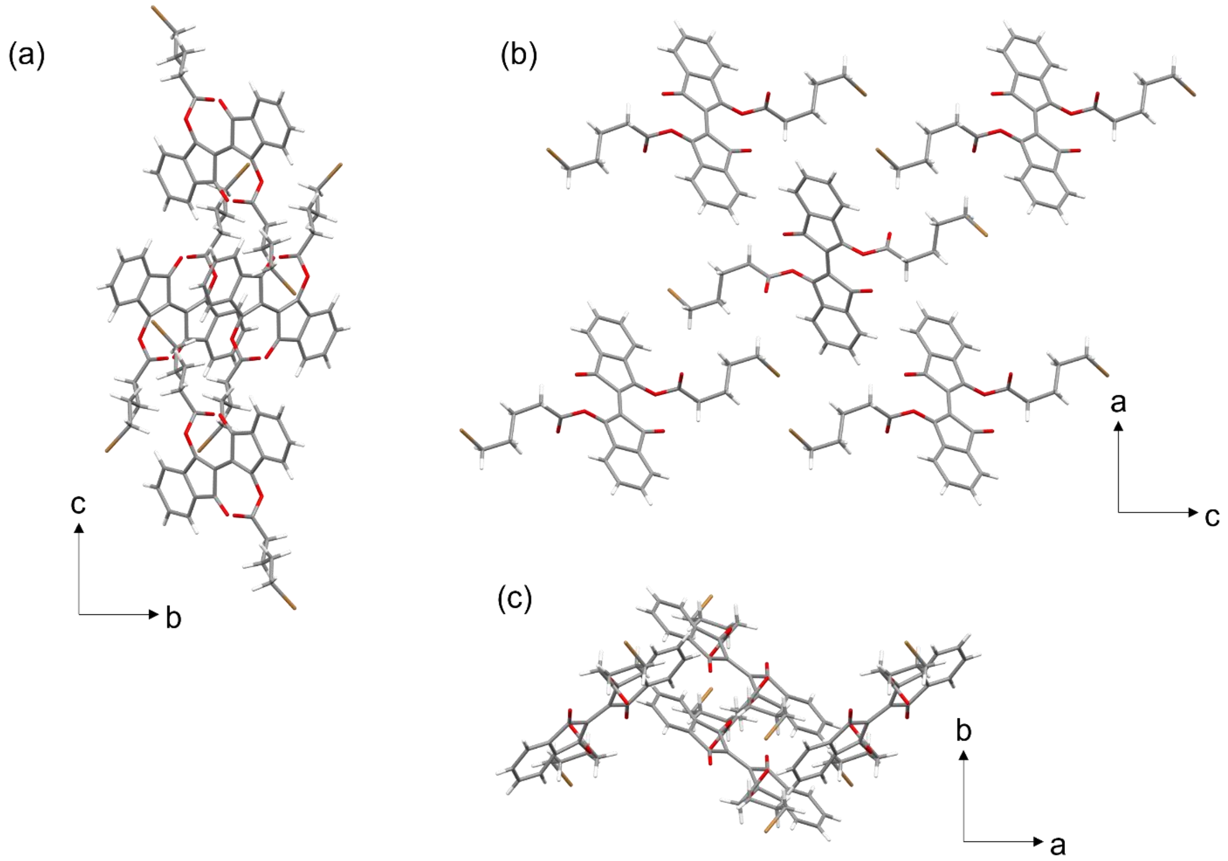

Figure S20. Single crystal X-ray diffraction results for **BIT-5-Br**. (a) View along a axis. (b) View along b axis. (c) View along c axis.

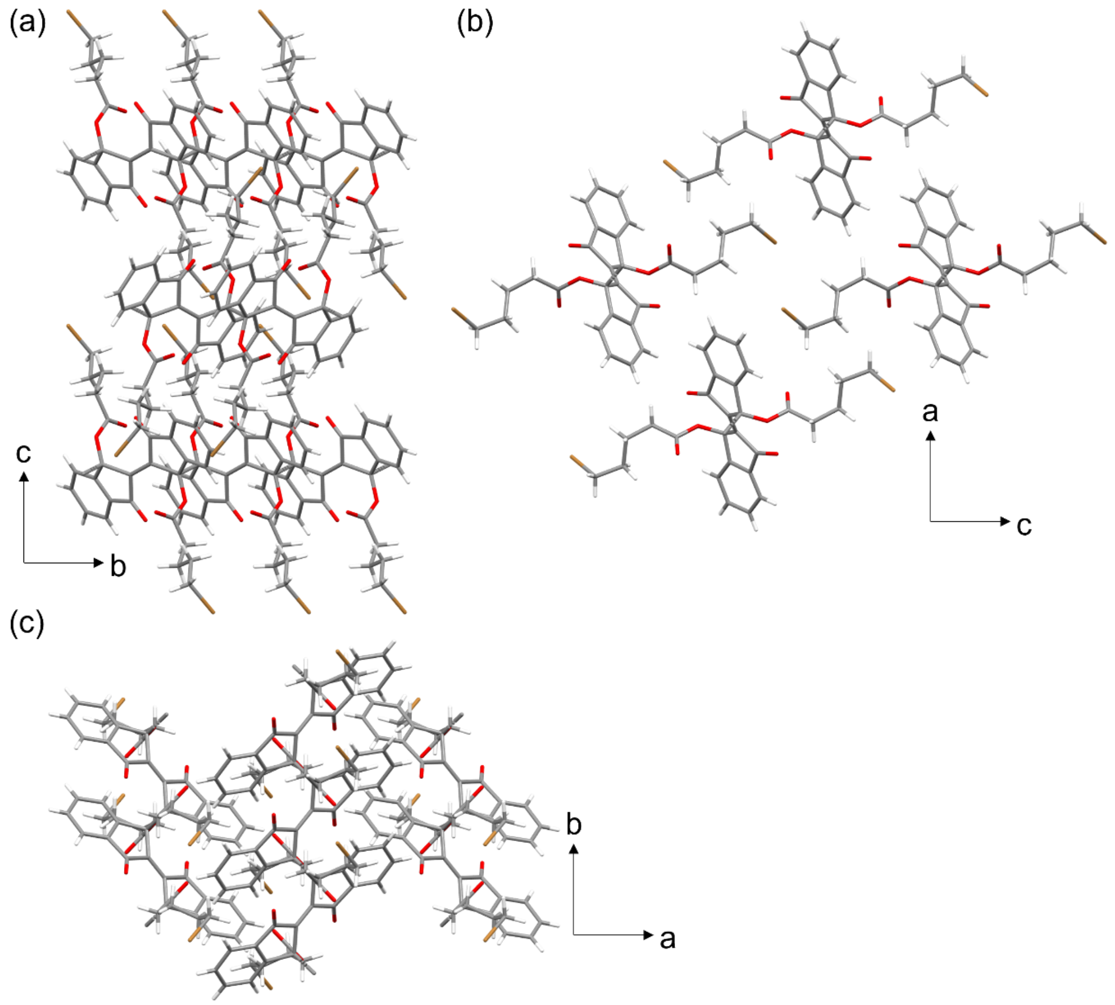

Figure S21. Single crystal X-ray diffraction results for **PBIT-5-Br**. (a) View along a axis. (b) View along b axis. (c) View along c axis.

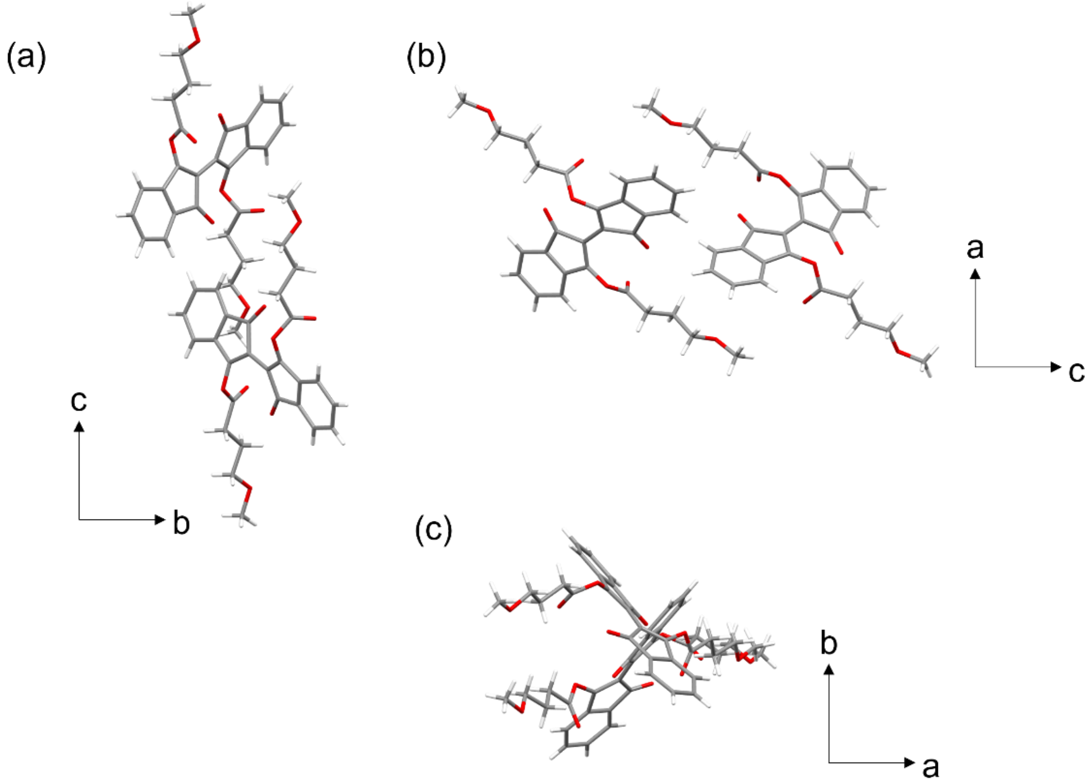

Figure S22. Single crystal X-ray diffraction results for **BIT-6-O**. (a) View along a axis. (b) View along b axis. (c) View along c axis. The polymer single crystal of BIT-6-O is missing because of the poor ambient stability of the monomer crystals.

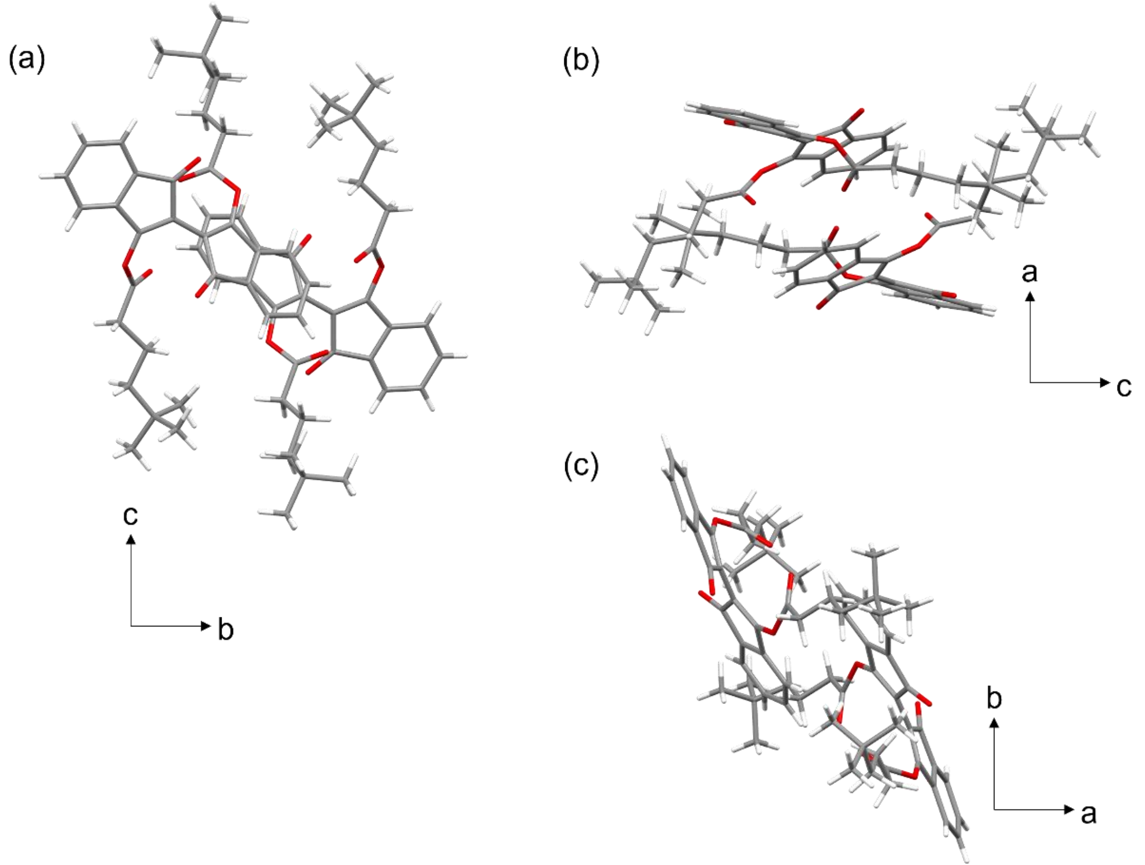

Figure S23. Single crystal X-ray diffraction results for **BIT-6-2Me**. (a) View along a axis. (b) View along b axis. (c) View along c axis.

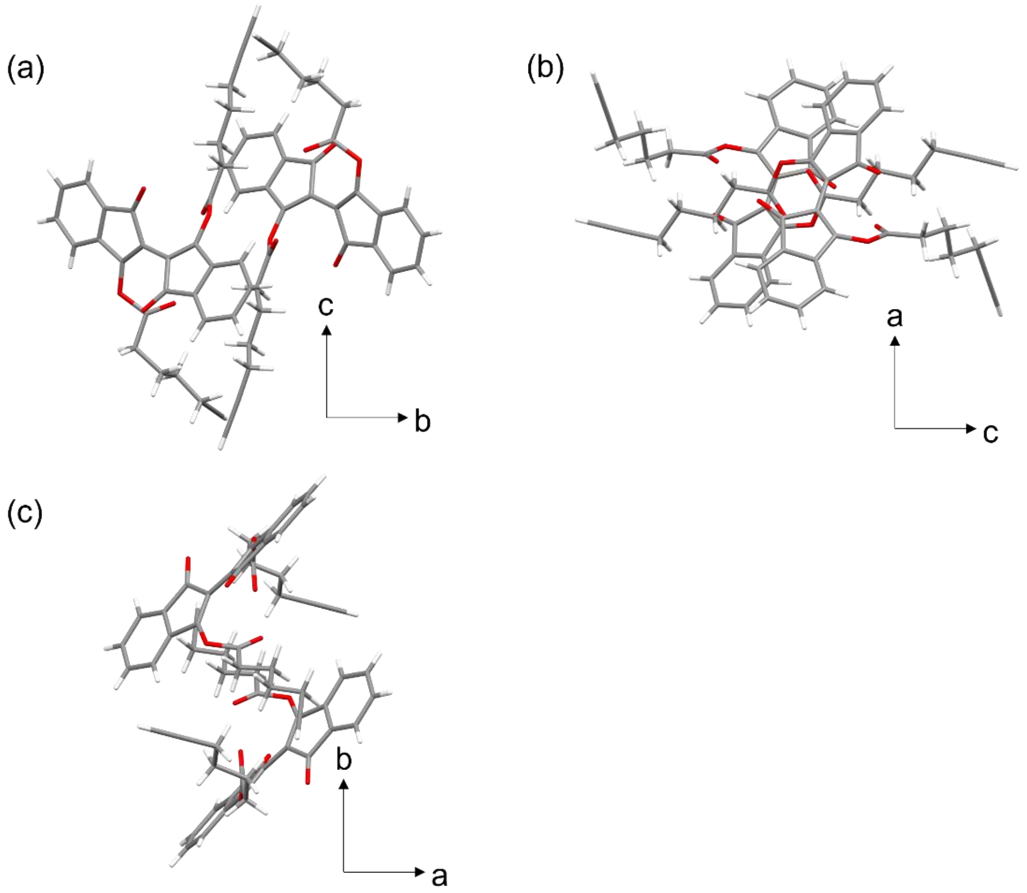

Figure S24. Single crystal X-ray diffraction results for **BIT-7T**. (a) View along a axis. (b) View along b axis. (c) View along c axis.

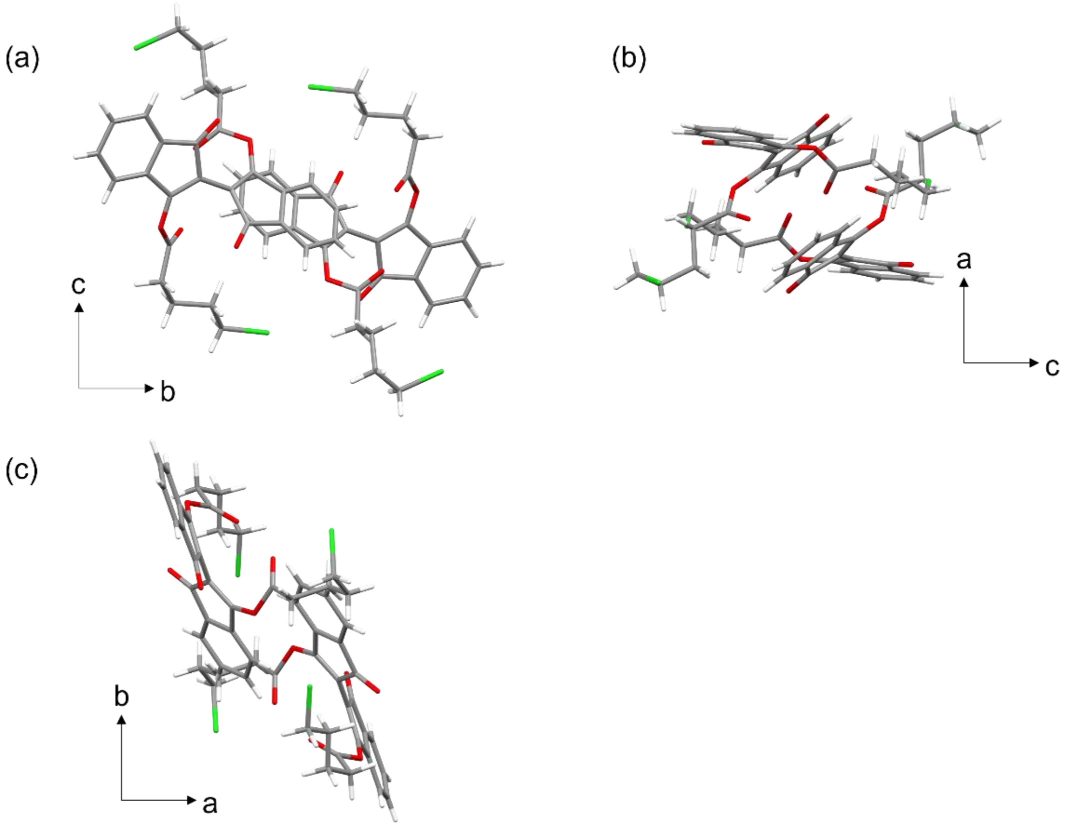

Figure S25. Single crystal X-ray diffraction results for **BIT-5-Cl**. (a) View along a axis. (b) View along b axis. (c) View along c axis.

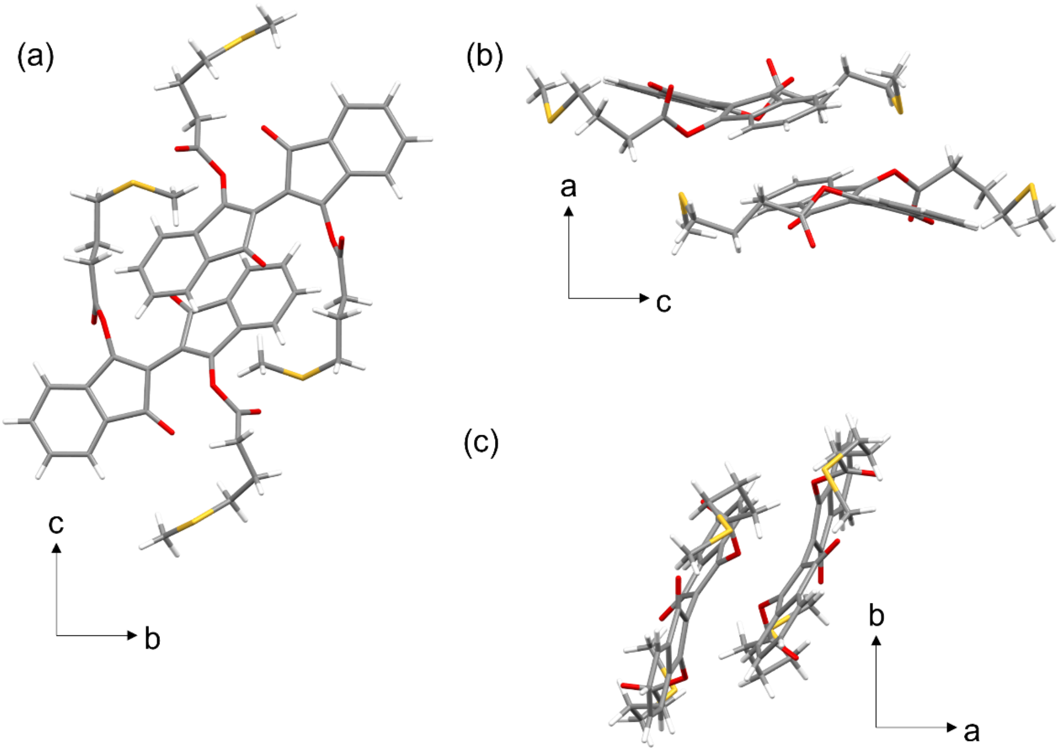

Figure S26. Single crystal X-ray diffraction results for **BIT-6-S**. (a) View along a axis. (b) View along b axis. (c) View along c axis.

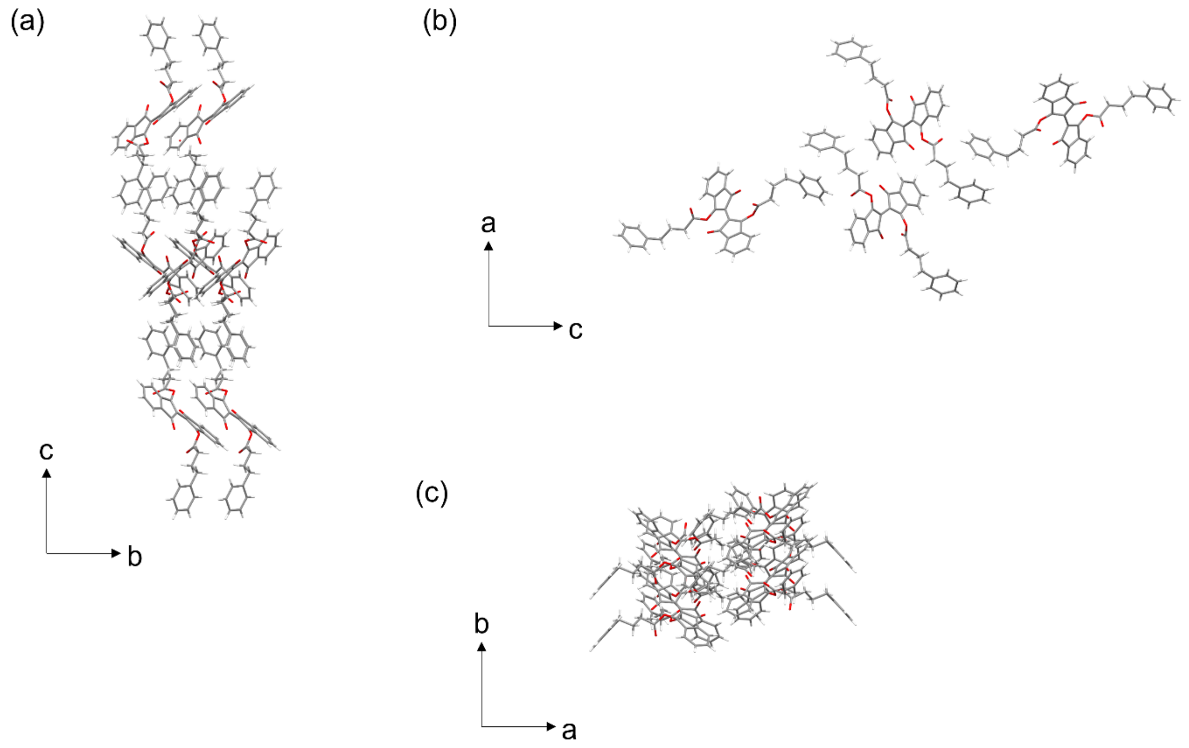

Figure S27. Single crystal X-ray diffraction results for **BIT-4-Ph**. (a) View along a axis. (b) View along b axis. (c) View along c axis.

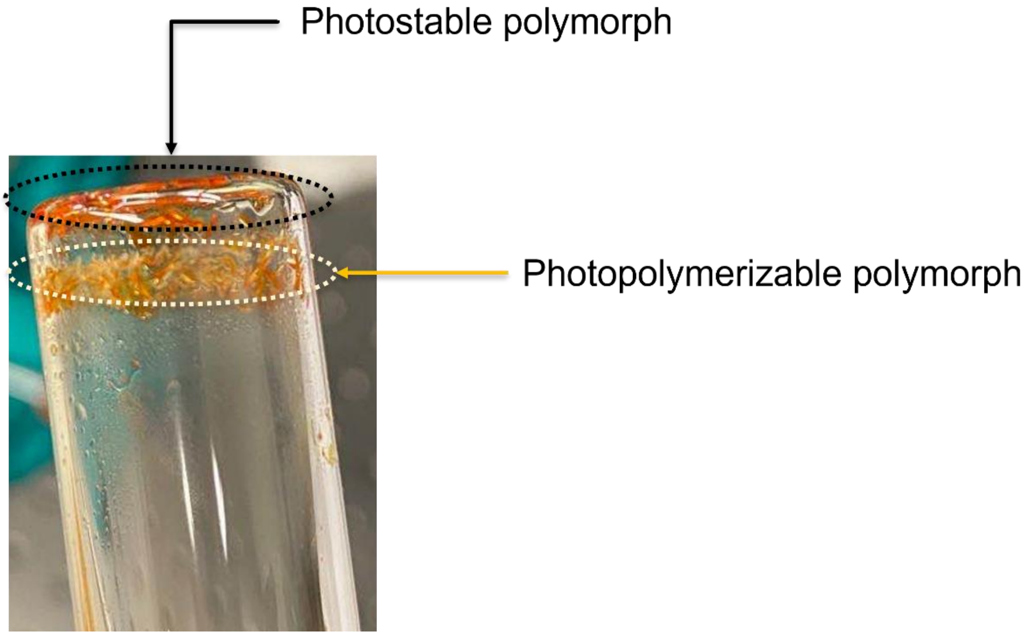

Figure S28. Images of two different polymorphs of BIT-5 crystals after shining visible light on both crystals overnight. Photostable polymorph is in the black dotted circle and photopolymerizable polymorph is in the yellow dotted circle. After shining visible light, photostable polymorph remains in orange but photopolymerizable polymorph turns into light yellow.

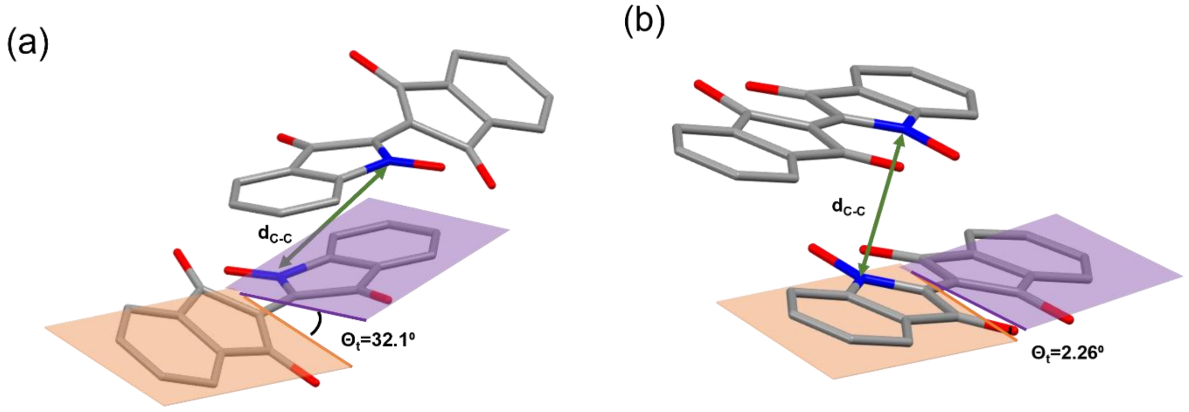

Figure S29. Crystal structure characterization of **BIT-7T** (a), and **BIT-6-Me** (b) monomers. Reactive site carbons are labeled in blue in monomers. All side chains and hydrogens are removed for clarity.

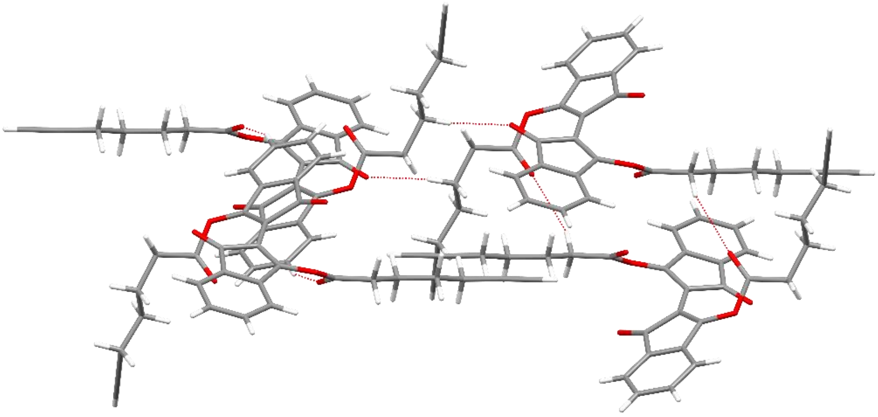

Figure S30. Intermolecular interaction analysis of **BIT-7T** crystal. Non-selective C−H···O hydrogen bonding (labeled in red dotted line) exisits among crystals.

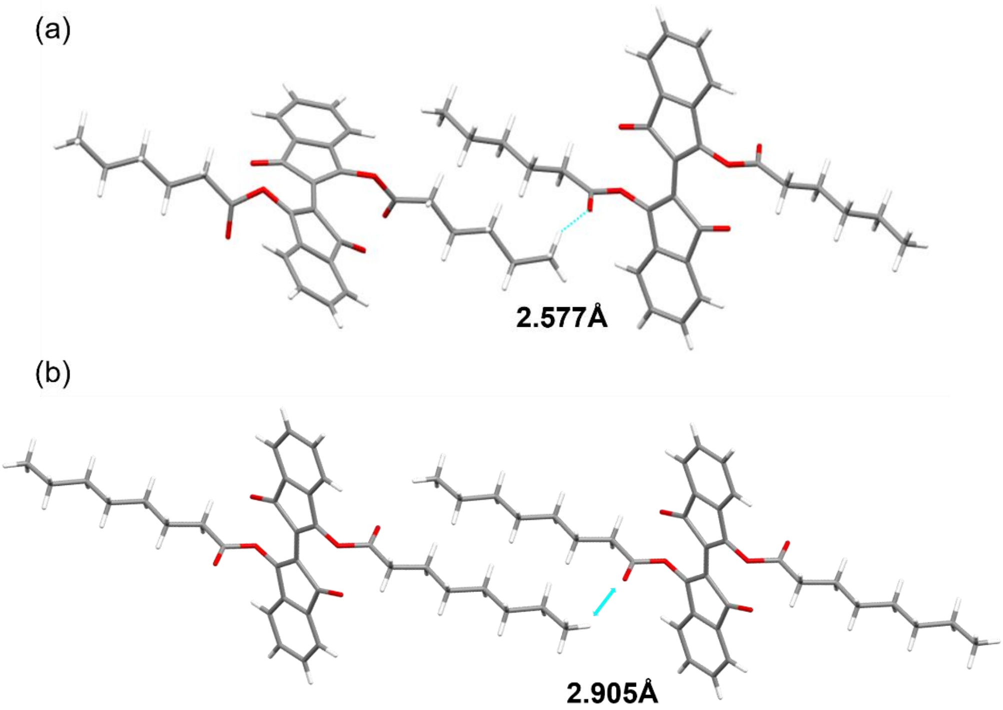

Figure S31. C−H···O Hydrogen bonding analysis among side chains on BIT-6 and PBIT-6. (a) Crystal structure of BIT-6. Hydrogen bonding between oxygen on the side chain carbonyl to the hydrogen on the end of another monomer's side chain is labeled in blue. (b) Crystal structure of PBIT-6. The weak hydrogen bonding on the end of the side chain becomes insignificant in polymer crystals.

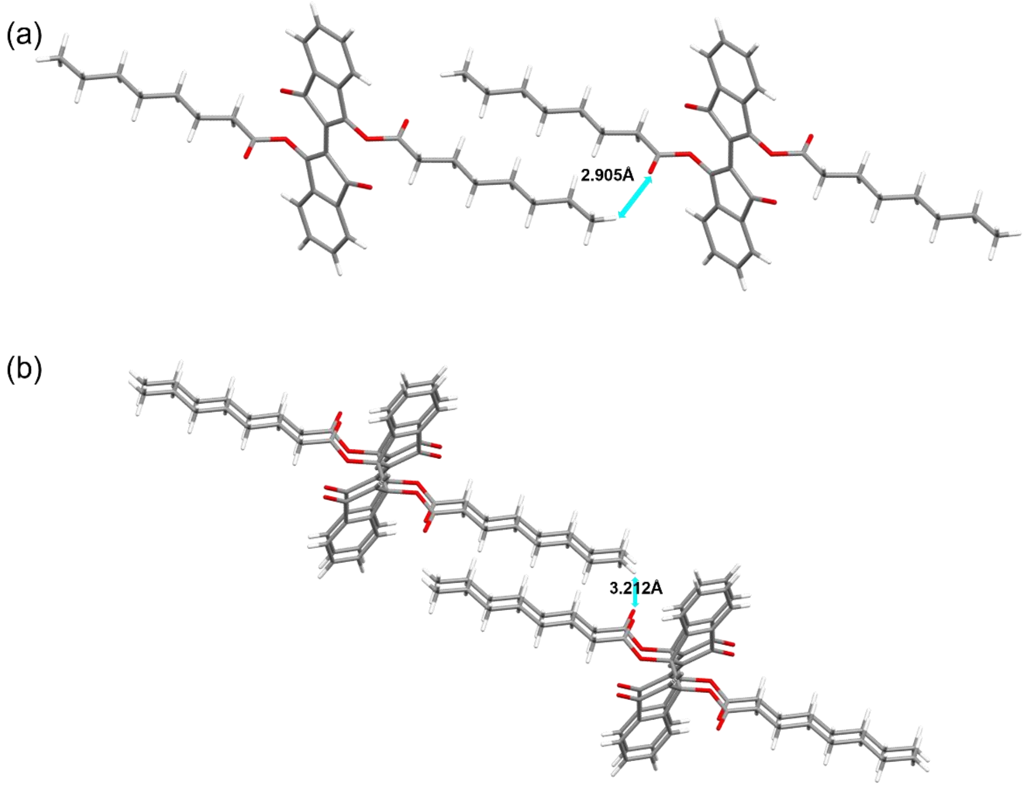

Figure S32. Single crystal analysis of **BIT-8** (a) and **PBIT-8** (b) with hydrogen bonding between siFe chains. In BIT-8, End hydrogens on side chains are 2.905Å away from the nearest oxygen, which is lager than the sum of their Van der Waals radii. In PBIT-8, this distance is further enlarged to 3.212Å.

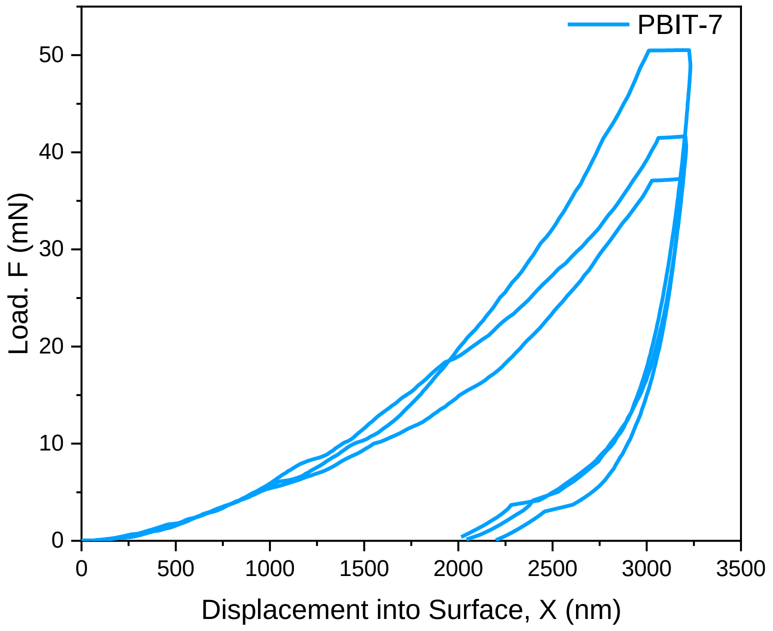

Figure S33. Load-displacement curves of **PBIT-7**.

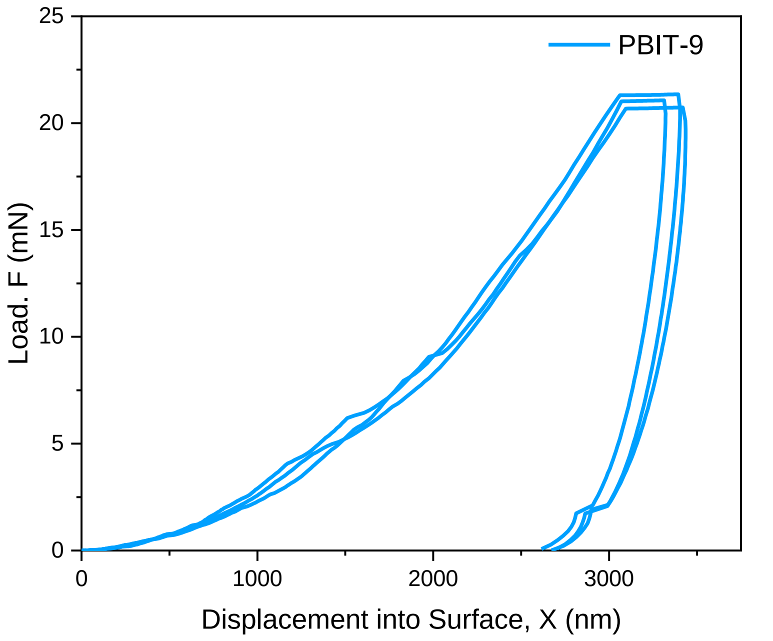

Figure S34. Load-displacement curves of **PBIT-9**.

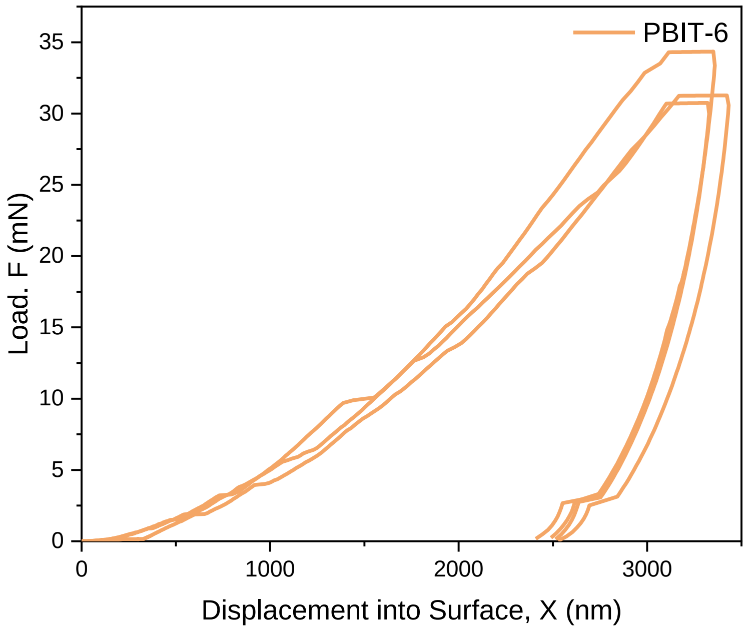

Figure S35. Load-displacement curves of **PBIT-6**.

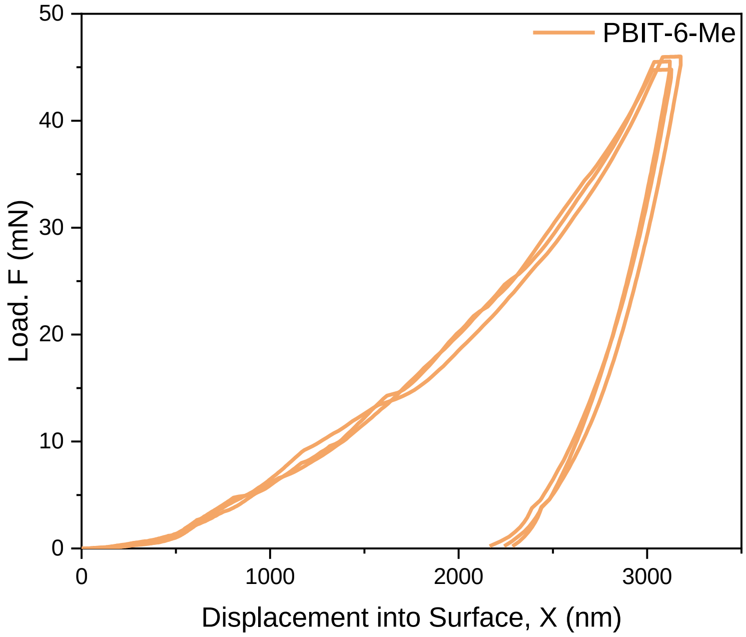

Figure S36. Load-displacement curves of **PBIT-6-Me**.

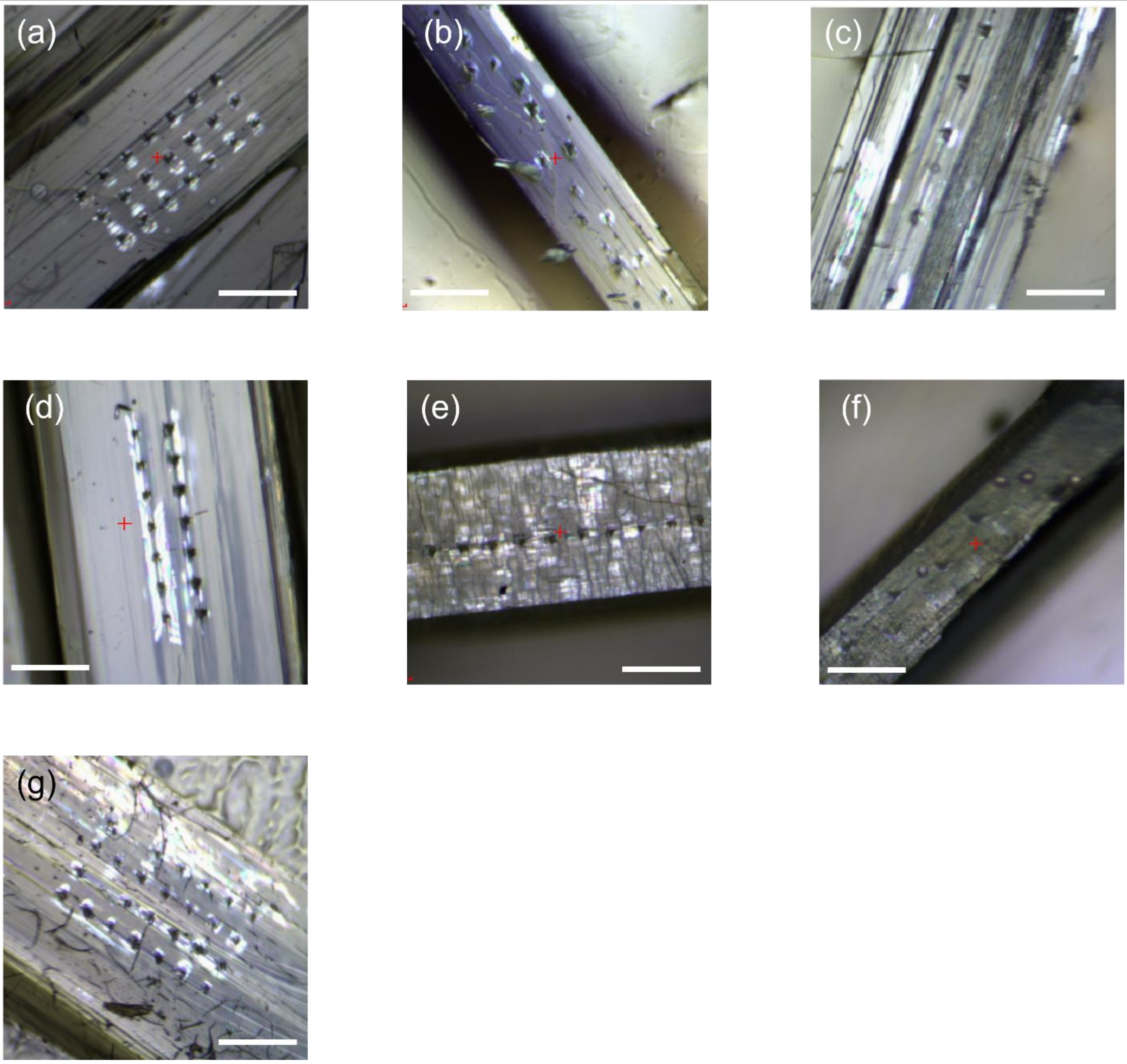

Figure S37. Snapshots of **PBIT-6** (a), **PBIT-7** (b), **PBIT-8** (c), **PBIT-9** (d), **PBIT-6-Me** (e), **PBIT-8D** (f), and **PBIT-5-Br** (g) polymer single crystals after nanoindentation experiments. Black triangular dots on the crystals are impressions of the indentation. Scale bar: 100 µm.

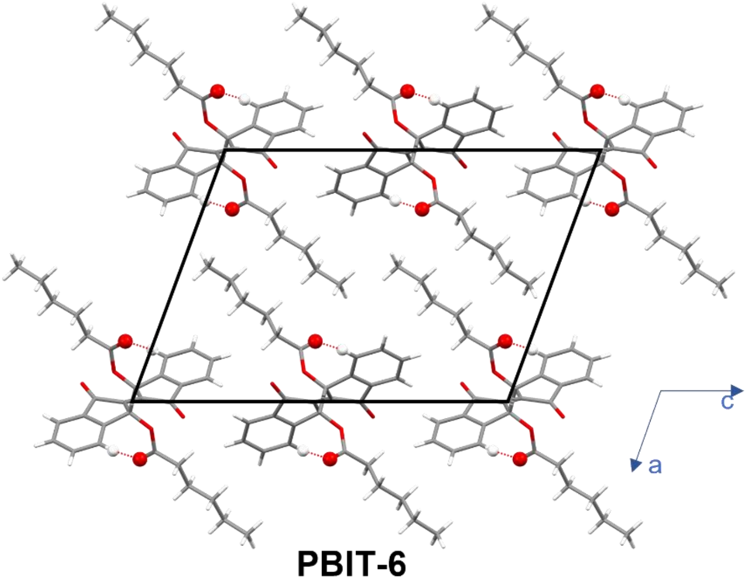

PBIT-6

Figure S38. Crystallography analysis of **PBIT-6** with side chains. No significant Interaction among polymer chains are recognized.

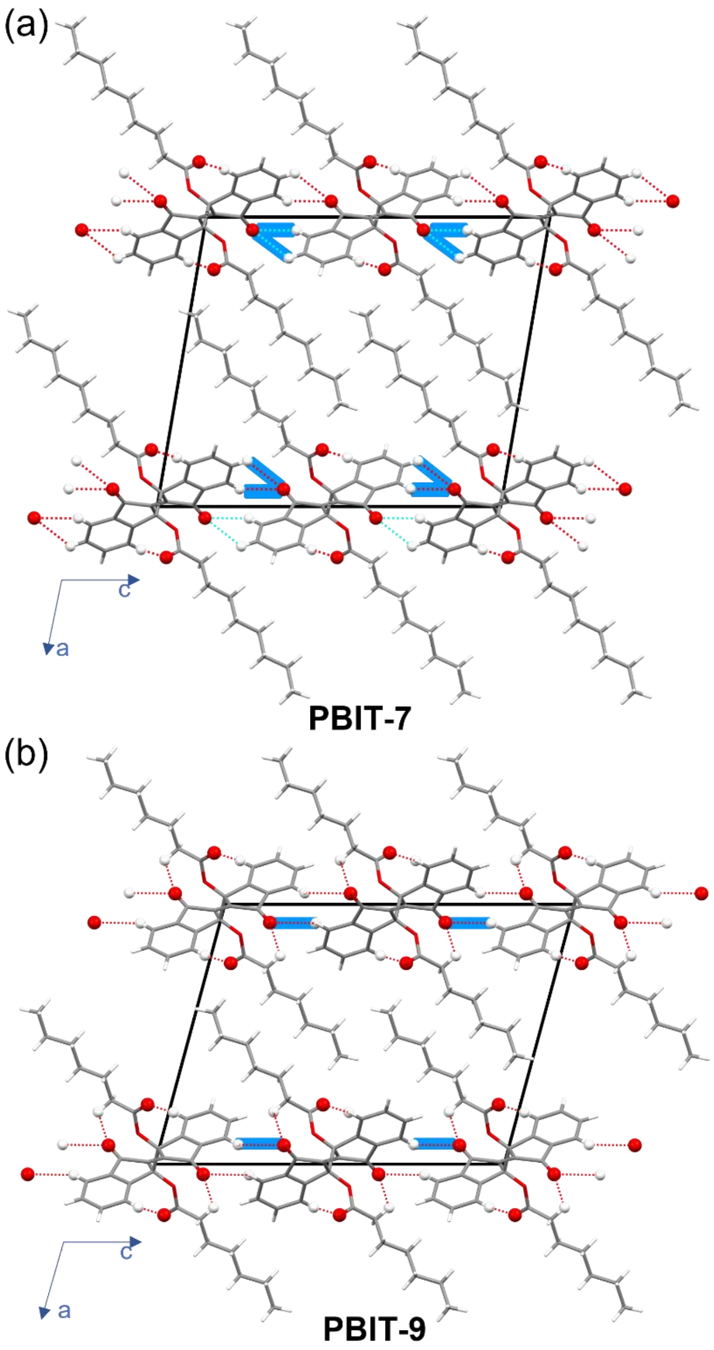

Figure S39. Crystallography analysis of PBIT single crystals with moderate elastic moduli. Hydrogen bonding among backbones is labeled in blue. (a) PBIT-7 single crystal structure with side chains. (b) PBIT-9 single crystal structure with side chains.

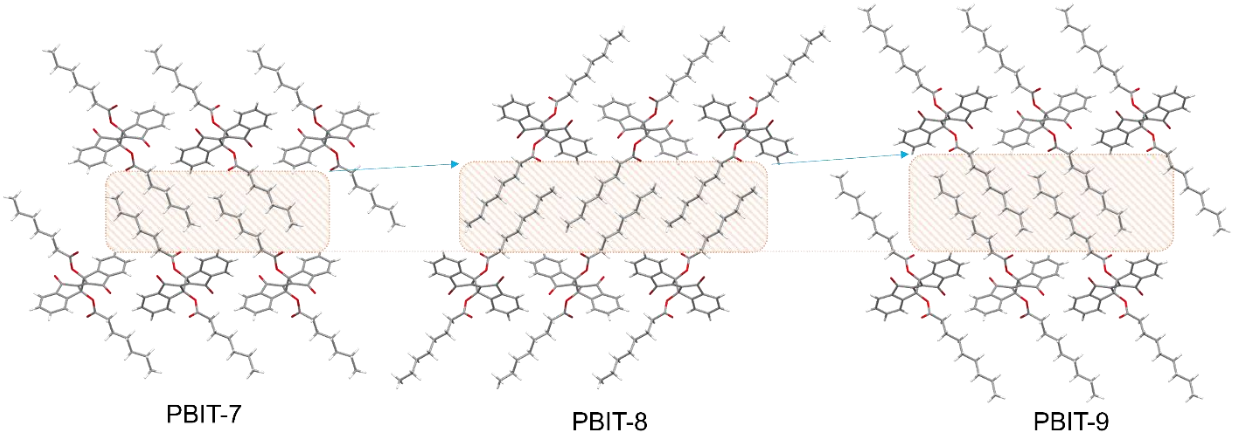

Figure S40. Comparison of “isolation regions” among **PBIT-7**, **PBIT-8**, and **PBIT-9** polymers single crystals. The “isolation region” is emphasized in rounded rectangle. As the side chain length increases, the isolation region expands.

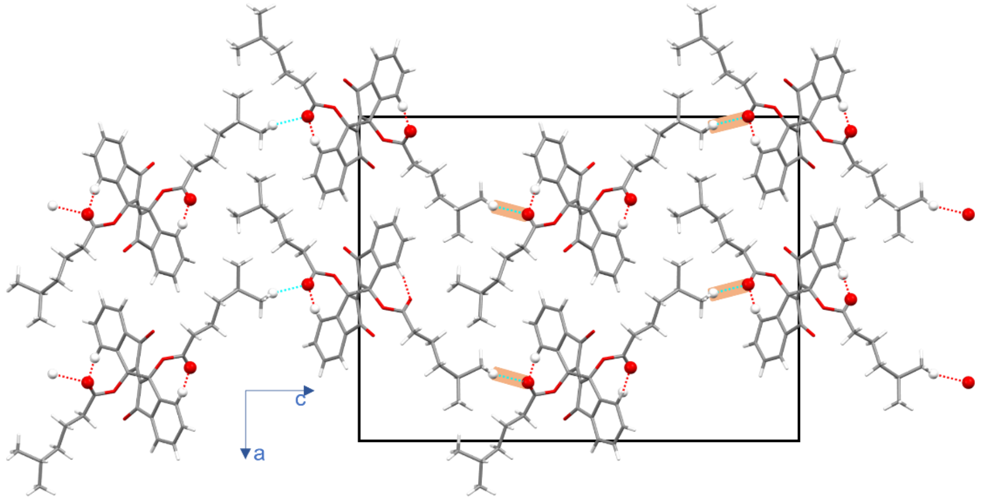

PBIT-6-Me

Figure S41. Crystallography analysis of PBIT-6-Me single crystals. Hydrogen bonding among side chains is labeled in beige.

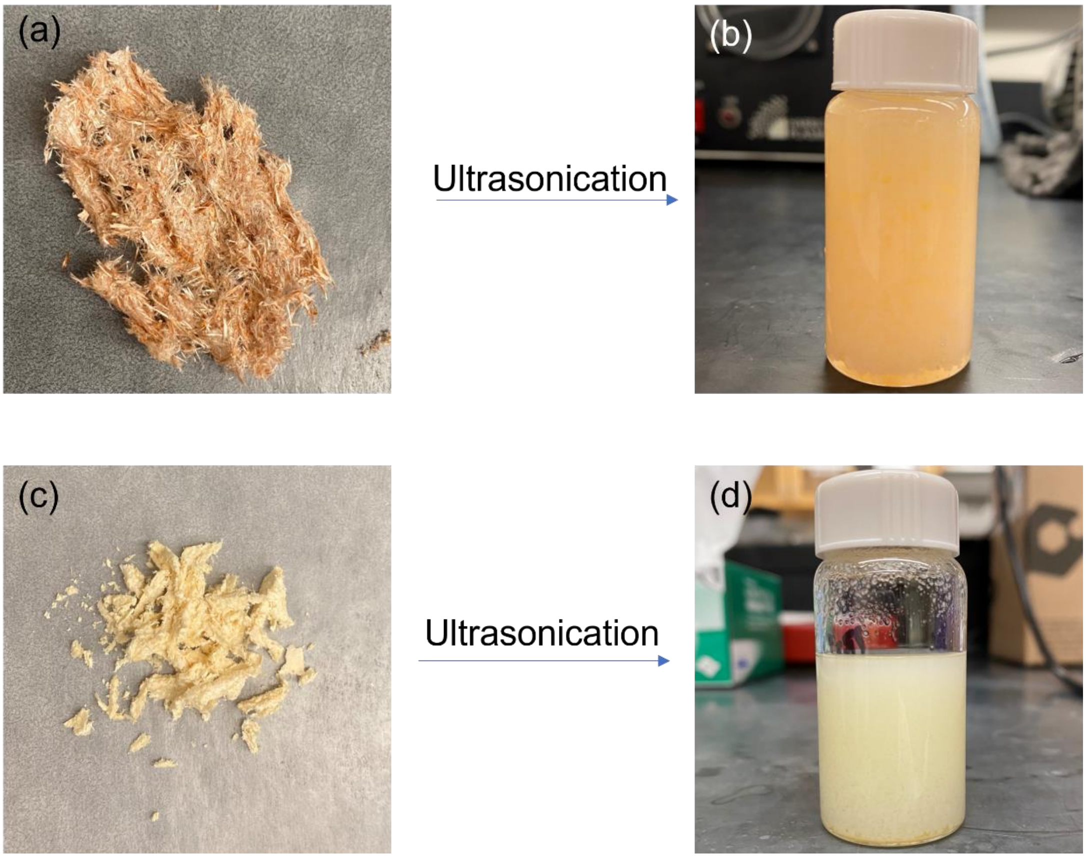

Figure S42. (a) PBIT-5-Br crystals before ultrasonication. (b) PBIT-5-Br suspended in chloroform after ultrasonication. (c) PBIT-6 crystals before ultrasonication. (d) PBIT-6 suspended in chloroform after ultrasonication.

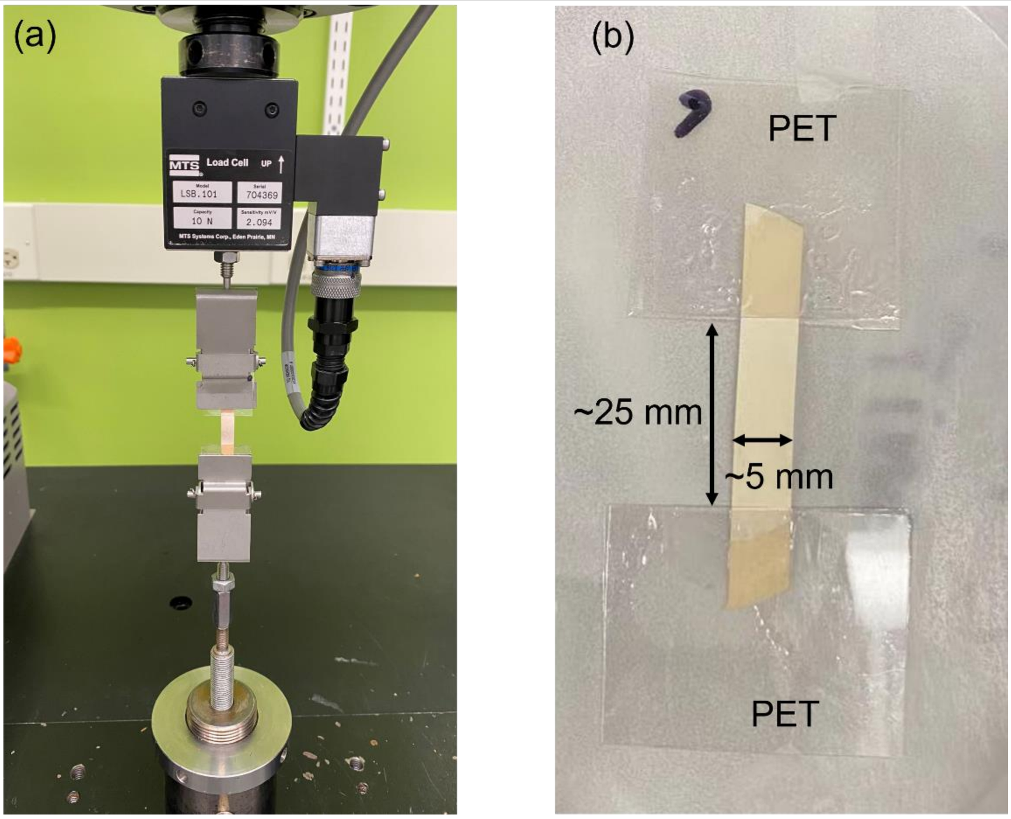

Figure S43. (a) Experimental setup of tensile tests. The polymer samples were fixed vertically on the tensile test machine and the 10 N load cell. The polymer sample was tested with a crosshead speed of 0.1 mm/s. (b) Images of PBIT-6 strip for tensile test.

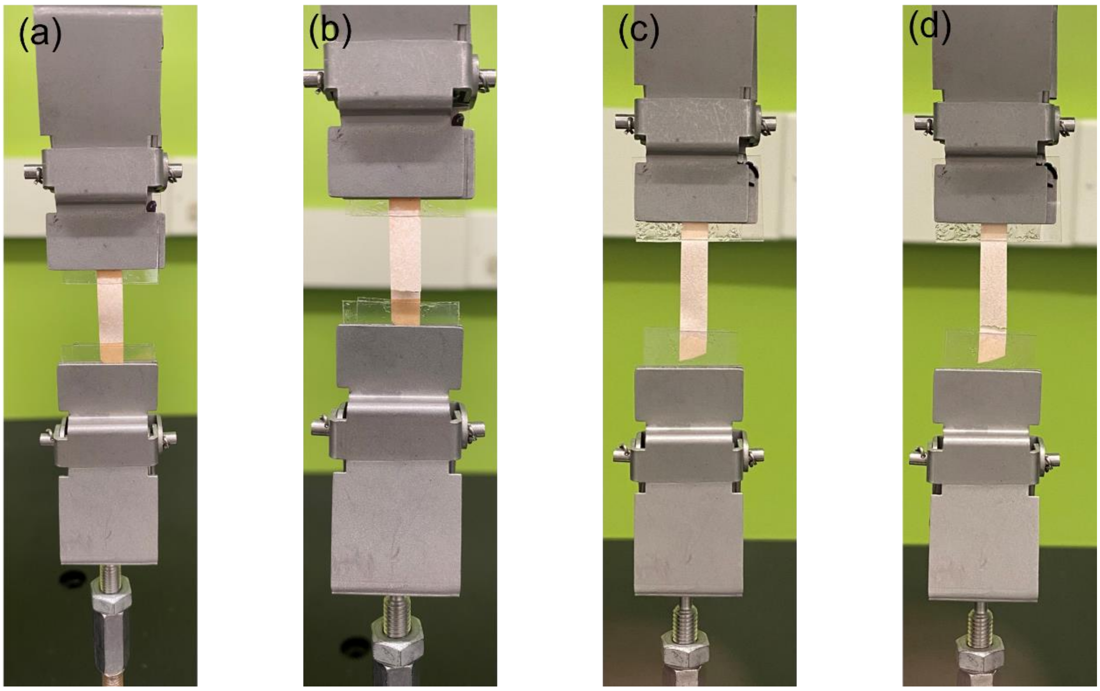

Figure S44. (a) PBIT-5-Br strip before tensile test. (b) PBIT-5-Br strip after tensile test. (c) PBIT-6 strip before tensile test. (b) PBIT-6 strip after tensile test.

Table S1. Crystallographic Data of BIT Monomers and Polymers

Crystal	BIT-6	PBIT-6	BIT-7	PBIT-7	BIT-8	PBIT-8	BIT-9	PBIT-9	BIT-6-Me	PBIT-6-Me
formula	$C_{30}H_{30}O_6$	$C_{30}H_{30}O_6$	$C_{32}H_{34}O_6$	$C_{32}H_{34}O_6$	$C_{34}H_{38}O_6$	$C_{34}H_{38}O_6$	$C_{36}H_{42}O_6$	$C_{36}H_{42}O_6$	$C_{32}H_{34}O_6$	$C_{32}H_{34}O_6$
color	orange	light yellow	orange	light yellow	orange	light yellow	orange	light yellow	orange	light yellow
crystal habit	needle	needle	needle	needle	needle	needle	needle	needle	needle	needle
crystal system	monoclinic	monoclinic	monoclinic	monoclinic	monoclinic	monoclinic	monoclinic	monoclinic	orthorhombic	orthorhombic
space group	$P2_1/n$	$P2_1/c$	$P2_1/c$	$P2_1/c$	$P2_1/n$	$P2_1/c$	$P2_1/c$	$P2_1/c$	$Pca\ 2_1$	$Pca\ 2_1$
a, Å	13.061(3)	13.6761(5)	13.9869(6)	14.313(5)	16.896(4)	16.4200(16)	15.998(7)	16.3802(12)	20.2049(9)	20.031(6)
b, Å	5.0983(12)	4.8676(2)	4.9373(3)	4.8632(17)	4.9242(10)	4.8651(4)	4.969(2)	4.8627(3)	4.9419(2)	4.8823(16)
c, Å	56.020(13)	19.5135(6)	19.6512(10)	19.271(7)	19.098(5)	19.2734(18)	19.449(7)	19.1505(13)	26.6465(10)	26.217(8)
α, deg	90	90	90	90	90	90	90	90	90	90
B, deg	91.418(10)	110.252(2)	100.022(2)	104.816(9)	114.109(11)	113.636(3)	93.64(2)	99.376(3)	90	90
γ, deg	90	90	90	90	90	90	90	90	90	90
Z	6	4	4	4	2	4	2	4	4	4
R factor	0.0494	0.0508	0.0439	0.0591	0.0381	0.0494	0.0434	0.0501	0.040	0.067

Table S1. Crystallographic Data of BIT Monomers and Polymers (continued)

Crystals	BIT-8D	PBIT-8D	BIT-5-Br	PBIT-5-Br	BIT-6-O	BIT-6-2Me	BIT-7T	BIT-5-Cl	BIT-6-S	BIT-5	BIT-4-Ph
formula	$C_{34}H_{34}O_6$	$C_{34}H_{34}O_6$	$C_{28}H_{24}Br_2O_6$	$C_{28}H_{24}Br_2O_6$	$C_{28}H_{26}O_8$	$C_{34}H_{38}O_6$	$C_{32}H_{26}O_6$	$C_{28}H_{24}Cl_2O_6$	$C_{28}H_{26}O_6S_2$	$C_{28}H_{26}O_6$	$C_{38}H_{30}O_6$
color	orange	light yellow	orange	light yellow	orange	orange	orange	orange	orange	orange	Orange
crystal habit	needle	needle	needle	needle	needle	plate	plate	plate	plate	plate	plate
crystal system	monoclinic	monoclinic	monoclinic	monoclinic	monoclinic	triclinic	triclinic	triclinic	triclinic	monoclinic	monoclinic
space group	$P2_1$	$P2_1/c$	$P2_1/n$	$P2_1/n$	$P2_1$	$P\bar{1}$	$P\bar{1}$	$P\bar{1}$	$P\bar{1}$	$P2_1$	$P2_1$
a, Å	13.912(3)	15.053(8)	15.410(2)	15.6444(12)	12.6130(5)	8.8623(4)	9.4106(9)	8.6220(6)	8.1893(4)	12.6988(13)	18.5047(9)
b, Å	5.1929(11)	4.856(2)	4.8363(5)	4.8408(4)	5.0456(2)	10.8849(4)	11.6491(11)	12.7088(10)	11.7702(7)	5.1534(5)	5.1944(3)
c, Å	19.343(4)	19.338(8)	17.275(2)	16.6523(11)	19.0508(7)	16.5121(7)	13.0853(13)	12.7994(10)	14.0284(8)	19.6329(18)	31.3477(16)
α, deg	90	90	90	90	90	89.1664(14)	74.774(6)	69.660(3)	104.719(2)	90	90
β, deg	96.316(7)	101.43(3)	107.242(7)	107.526(4)	101.273(2)	85.2804(14)	79.798(6)	73.298(3)	98.683(2)	108.283(5)	106.779(2)
γ, deg	90	90	90	90	90	76.4793(13)	69.730(6)	72.422(3)	102.996(2)	90	90
Z	2	4	2	4	2	4	2	2	2	2	4
R factor	0.0680	0.1447	0.0734	0.0577	0.0366	0.0421	0.0673	0.0476	0.0388	0.0408	0.0632

Table S2. PBIT crystal densities

Polymer	Crystal Density (g/cm^3)
PBIT-6	1.326
PBIT-7	1.318
PBIT-8	1.278
PBIT-9	1.259
PBIT-8D	1.291
PBIT-6-Me	1.333
PBIT-5-Br	**1.702**

3. References

[1] a) G. Kresse, J. Hafner, *Phys. Rev. B* **1993**, *47*, 558-561; b) G. Kresse, J. Furthmüller, *Comput. Mater. Sci.* **1996**, *6*, 15-50; c) G. Kresse, J. Furthmüller, *Phys. Rev. B* **1996**, *54*, 11169-11186.

[2] a) P. E. Blöchl, *Phys. Rev. B* **1994**, *50*, 17953-17979; b) G. Kresse, D. Joubert, *Phys. Rev. B* **1999**, *59*, 1758-1775.

[3] J. P. Perdew, K. Burke, M. Ernzerhof, *Phys. Rev. Lett.* **1996**, *77*, 3865-3868.

[4] A. Tkatchenko, M. Scheffler, *Phys. Rev. Lett.* **2009**, *102*, 073005.

[5] V. Wang, N. Xu, J. C. Liu, G. Tang, W. Geng, *Comput. Phys. Commun.* **2021**, *267*, 108033.

4. Author Contributions

L.D. conceived the idea and supervised the project. Z.W. carried out the materials synthesis, single crystal preparations, characterizations, and data analysis. X.W. and K.Z. carried out the nanoindentation tests and data analysis. B.S. and B.M.S. carried out the DFT calculations. X.L., Q.H. and J.J. helped. on the materials synthesis and reactions optimizations. M.Z. carried out X-ray diffraction measurements and data collections. K.W. helped design UV-vis absorption measurements. Z.W. and L.D. wrote the manuscript. All authors discussed the results and revised the manuscript.
